# Synthesis of Novel Arginine Building Blocks with Increased Lipophilicity Compatible with Solid-Phase Peptide Synthesis

**DOI:** 10.3390/molecules28237780

**Published:** 2023-11-25

**Authors:** Mladena Glavaš, Agata Gitlin-Domagalska, Natalia Ptaszyńska, Dominika Starego, Sylwia Freza, Dawid Dębowski, Aleksandra Helbik-Maciejewska, Anna Łęgowska, Chaim Gilon, Krzysztof Rolka

**Affiliations:** 1 Department of Molecular Biochemistry, Faculty of Chemistry, University of Gdansk, Wita Stwosza 63, 80-308 Gdansk, Poland; mglavas@irb.hr (M.G.); natalia.ptaszynska@ug.edu.pl (N.P.); dominika.starego@phdstud.ug.edu.pl (D.S.); dawid.debowski@ug.edu.pl (D.D.); aleksandra.maciejewska@phdstud.ug.edu.pl (A.H.-M.); anna.legowska@ug.edu.pl (A.Ł.); krzysztof.rolka@ug.edu.pl (K.R.); 2 Department of Organic Chemistry and Biochemistry, Ruđer Bošković Institute, Bijenička cesta 54, 10 000 Zagreb, Croatia; 3 Department of Theoretical Chemistry, Faculty of Chemistry, University of Gdansk, Wita Stwosza 63, 80-308 Gdansk, Poland; sylwia.freza@ug.edu.pl; 4 Department of Organic Chemistry, Institute of Chemistry, The Hebrew University, Jerusalem 91904, Israel; chaim.gilon@ug.edu.pl

**Keywords:** arginine building blocks, increased lipophilicity, SPPS, vasopressin, prodrugs

## Abstract

Arginine, due to the guanidine moiety, increases peptides’ hydrophilicity and enables interactions with charged molecules, but at the same time, its presence in a peptide chain might reduce its permeability through biological membranes. This might be resolved by temporary coverage of the peptide charge by lipophilic, enzyme-sensitive alkoxycarbonyl groups. Unfortunately, such a modification of a guanidine moiety has not been reported to date and turned out to be challenging. Here, we present a new, optimized strategy to obtain arginine building blocks with increased lipophilicity that were successfully utilized in the solid-phase peptide synthesis of novel arginine vasopressin prodrugs.

## 1. Introduction

Arginine (Arg, R) is the most basic natural amino acid [1,2,3] with the highest proton affinity (245.2 kcal/mol), while the second is histidine (231.2 kcal/mol) [4]. The p*K*_a_ value of the guanidine group located in the side chain of Arg, measured in water, is lower (~12.5) than that of guanidine itself (~13.5) [2,3]. The Y-delocalized guanidine moiety is stabilized by resonance. There are six potential hydrogen bond donors, and it is highly soluble in an aqueous system [1,3]. Therefore, it is responsible for electrostatic and hydrogen bond interactions with anionic and polar molecules, especially in ligand–receptor or substrate–enzyme interactions [1,2]. Furthermore, Arg residue present in peptide chains participates in cation-π interactions, which are made up of the attractive force between cationic species and π-bonded systems (aromatic ring, allylic group) [3,5]. Gallivan and Dougherty [6] found that cation-π interactions are common among the Protein Data Bank (PDB) structures, and on average one energetically significant cation-π interaction may be expected for every 77 residues in the protein. Furthermore, this study showed that Arg and Trp are the most likely to be involved in cation-π interactions, while over 70% of the Arg side chains were found in proximity to an aromatic side chain [3,6]. These observations underline the significance of cation-π interactions for protein structure, equally to salt bridges, hydrogen bonds, etc. Natural and synthetic guanidine-containing compounds have attracted great interest not only due to their involvement in various biological processes, but also because of their applications as catalysts [7], as enantioselective or substrate-specific oxoanion hosts [7], in the study of DNA-drug interactions [3,7] or in the synthesis of peptides and peptidomimetics with specific biological activity [7]. 

**Figure 1:**
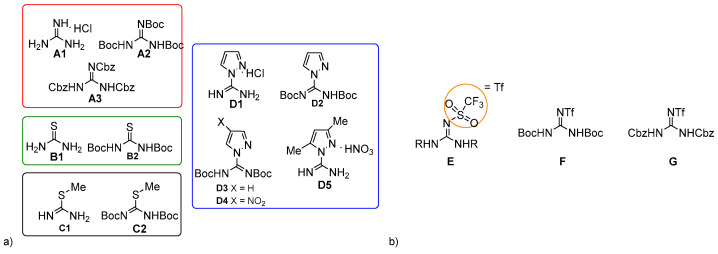
(**a**) The most prevalent guanidinylating reagents; (**b**) general structure of *N*-triflyl guanidines (**E**) and its derivatives *N*,*N*′-di-Boc-*N*″-triflylguanidine (**F**) and *N*,*N*′-di-Cbz-*N*″triflylguanidine (**G**).

The preparation of guanidine-containing compounds compatible with solid-phase peptide synthesis (SPPS), under mild conditions, with high yield and low cost is of great interest in peptide chemistry [2]. In general, their synthesis involves the treatment of an amine with an electrophilic amidine species. Various aliphatic, aromatic and heterocyclic compounds and sugars or amino acids, etc., can be used as an amine component. The most prevalent guanidinylating reagents are presented in Figure 1a: guanidine hydrochloride (**A1**) [1,2,8,9] thiourea (**B1**) [1,10] or *S*-methylisothiourea (**C1**) [1,11], pyrazole-1-carboxamidine hydrochloride (**D1**) [1,12,13,14] and their derivatives. The conversion of amines into guanidines with thiourea and *S*-methylthiourea requires initial activation with mercury [10,15,16,17,18] or copper salts [18], diisopropylcarbodiimide (DIC) [19], *N*-iodosuccinimide (NIS) [20], 1-(3-dimethylaminopropyl)-3-ethylcarbodiimide (EDC) [21] or 1-methyl-2-chloropyridinium iodide (Mukaiyama’s reagent) [10,17,20,21,22]. Amines can also be converted into guanidines by using *S*-methylthiourea preactivated by methyl iodide [23]. *N*-trifluoromethanesulfonyl (triflyl) guanidines (**E**, Figure 1b) were described for the first time in 1998 [2,8] and represent a novel class of functionalized guanidines efficient for guanidinylation of amines [24], amino acid derivatives and peptides in solution [8], as well as on the solid support [2,8]. Various approaches were taken to obtain *N*-triflyl guanidines, i.e., amination [25,26,27,28] or one-pot reactions [29]. Their remarkable reactivity comes from the high nucleofugality of the triflyl group [24]. *N*,*N*′-di-Boc-*N*″-triflylguanidine (Goodman reagent; **F**, Figure 1b) and *N*,*N*′-di-Cbz-*N*″triflylguanidine (**G**, Figure 1b) were the first guanidinylating reagents described [2]. To prove their potency, various amines, amino alcohols and amino ethers, as well as amino acids and peptides, were subjected to guanidinylation, resulting in at least 75% yield. For both reagents, the best results were obtained for unhindered, primary amines, with the yield exceeding 90%. What is more, **F** was demonstrated to be able to react with free amines formed in situ from azides, both primary and secondary, in carbohydrate scaffolds [22]. This discovery opened the possibility of obtaining guanidine derivatives from azides in a one-pot reaction. Witkowska et al. [1] compared guanidinylation of peptides on resin with various reagents using microwaves. In this study, the results obtained for compound **F** were not so appealing, as the introduction of microwaves led to a significant amount of side products. 

**Figure 2:**
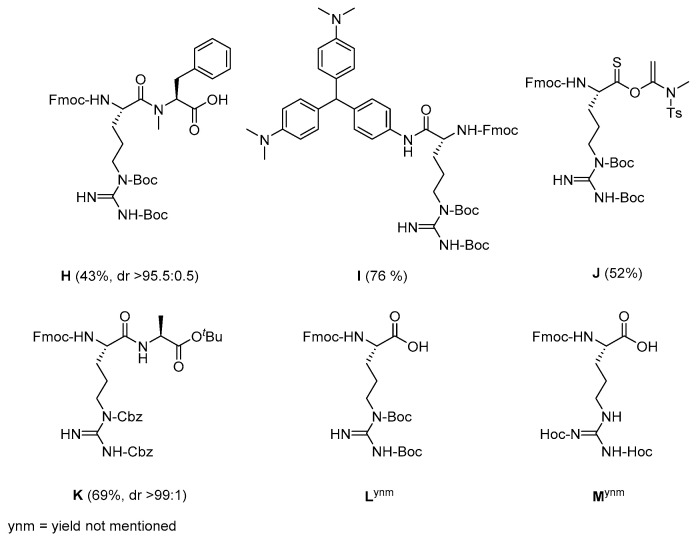
Arginine derivatives with masked side chain (Ts = tosyl group; Hoc = hexyloxycarbonyl).

The literature shows few examples of syntheses of arginine derivatives with side chains masked by two alkoxycarbonyl groups. To date, papers have focused mostly on two protecting groups, i.e., Boc and Cbz (**H**–**L**, Figure 2) [30,31,32,33,34], and their introduction is well described. In 2017, Weinmüller et al. [35] reported the synthesis of Arg with side chains masked with two hexyloxycarbonyl groups (**M**, Figure 2), which was further used in the synthesis of a peptide prodrug. However, the yield of the obtained Arg derivative was not reported. A similar approach was described by Schumacher-Klinger et al. [36], who also used the mentioned Arg derivative to obtain *N*-methylated hexapeptide prodrug. They demonstrated that such a modified peptide, with the Arg charge masked by two alkoxycarbonyl groups, is rapidly processed by serum esterases to the mother hexapeptide. This work laid a foundation of lipophilic prodrug charge masking (LPCM) strategy [36,37] that is a new approach to developing orally administered prodrugs of biologically relevant peptides.

Peptides, due to their key biological functions in living organisms and unique properties, seem to have excellent potential to be medications [38]. Currently, 6% of all Food and Drug Administration (FDA)-approved drugs are classified as peptides [39]. They are highly potent and specific to biological targets (enzymes, receptors) [40] and metabolized to non-toxic amino acids, and their synthesis, both in solution and on solid support, is well mastered. However, they are not free of certain drawbacks, such as low resistance to proteolysis, rapid clearance due to metabolic processing and often high hydrophilicity, which results in poor oral bioavailability and epithelial membrane permeability [40]. LPCM is an attitude that assumes an increase of peptide liphophilicity and, in effect, improved permeability. This strategy aims at the transformation of hydrophilic peptides into orally bioavailable prodrugs. In short, in this concept, a peptide charge is transiently masked by alkoxycarbonyl group(s), and a lipophilic peptide prodrug, with improved permeability through biological membranes, is obtained. After it reaches the blood circulation, the ubiquitous carboxyesterases cleave this temporary masking and the mother peptide drug is released [41,42,43,44]. It is important to underline that a moiety introduced onto the guanidino group of Arg must be recognized by carboxyesterases; thus, this strategy aims at the introduction of alkoxycarbonyl-masking groups.

Taking into consideration the literature data presented above, we decided to examine the applicability of the LPCM method to peptides that are commonly used in medicine as drugs, however, not delivered orally. Thus, we intended to synthesize series of peptides and their charge-masked prodrugs with various masking groups, in various positions. One of them is arginine vasopressin (AVP), which is a nonapeptide composed of a tripeptide tail and a six-membered ring with a disulfide bridge between cysteines at positions 1 and 6 [45]. It is found in mammals, binds to three G-protein-coupled receptors (V1a, V1b and V2), and depending on the binding site, plays different roles, such as vasoconstriction, glycogenolysis, mood, modulation of ACTH synthesis, water retention, stimulation of insulin synthesis, etc. In order to synthesize an AVP prodrug, an Arg derivative with a masked side chain charge was needed. Our first idea for the synthesis of such Fmoc-Arg derivatives was side chain chloroformate protection of Fmoc-Arg (Scheme S1), although it was not straightforward and resulted in obtaining two isomers of the product. The Arg transformation procedure described previously [35] was also not successful and we did not obtain the desired product. Unfortunately, the up-to-date literature delivers no more examples of alkoxycarbonyl group introduction to the Arg side chain, and as Arg derivatives with two alkyloxycarbonyl groups attached to the guanidinium moiety are not commercially available, we needed to develop a new method of their preparation. We decided to a different approach, namely guanidinylation of Orn, that is successfully utilized in chemistry [8,46]. Feichtinger et al. [2] applied guanidynylation of Orn to introduce Boc or Cbz groups with 82% and 85%, respectively. However, this method was not reported to obtain Arg derivatives bearing alkoxycarbonyl side chain protection. Our work presents the development and optimization of the synthesis of guanidinylating reagents with various lengths of alkyl chains (ethyl to dodecyl), their further application for guanidinylation of Orn to increase lipophilicity, and the synthesis of AVP analogues. 

## 2. Results and Discussion 

**Scheme 1:**
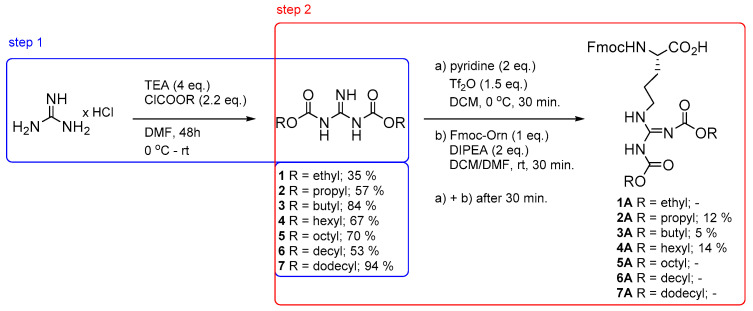
Synthetic pathway for the preparation of arginine building blocks with increased lipophilicity via guanidinylation of Fmoc-Orn.

Our initial idea to synthesize Arg derivatives **1A**–**7A** (Scheme 1) was the direct side chain protection of Fmoc-Arg with chloroformate (Scheme S1). The first step was the protection of the *α*-carboxyl group of Fmoc-Arg. We tried to introduce various protecting groups such as methoxy, benzyl, trityl or *tert*-butoxy; however, it turned out to be successful only in case of methoxy and benzyl groups. For the optimization of reaction conditions, we used Fmoc-Arg-OMe and the ethyl chloroformate (entries 1–8, Table S1. The best yield of 88% was achieved using DIPEA in DCM under reflux (entry 8, Table S1), but the purification using silica gel column chromatography resulted in two compounds (structures shown in Figure S1) with almost the same retention time. The structures of these compounds were confirmed via NMR (Figures S2–S5). Even though the final product was a mixture, we decided to continue to the next step, which was the deprotection of the α-carboxyl group. However, together with methoxy group removal, we observed cleavage of the Fmoc group (various conditions tested, data not presented in this work), crucial for SPPS. A new protection with Fmoc was unsuccessful (various conditions tested, data not presented in this work). In the case of the Fmoc-Arg-OBz, benzyl deprotection was also problematic, as the decomposition of the product was observed. Due to the problems with the protection/deprotection and detection of isomers **1C** and **1D** (Figure S1), we decided to abandon this approach.

Our next strategy, to obtain arginine derivatives with increased lipophilicity, was guanidinylation of Fmoc-Orn. The synthetic pathway consisted of two stages (Scheme 1). These were step 1, aimed at the optimization of guanidinylating reagent synthesis and step 2, which was a one-pot reaction assuming guanidinylation of Fmoc-Orn. Each step was optimized to find the best conditions for the most efficient reaction; details are given in the further parts of this paper.

The synthesis started with the preparation of guanidinylating reagents by using guanidine hydrochloride and different chloroformates (step 1, Scheme 1). Butyl chloroformate was used to optimize reaction conditions for step 1 (entries 1–8, Table 1). In the first three entries, triethylamine (TEA) as a base was applied and the reaction was performed in a mixture of acetonitrile/water in different ratios and under different temperatures. Additionally, butyl chloroformate was preactivated with 4-dimethylaminopyridine (DMAP; entry 2, Table 1). However, a low yield (entry 1, Table 1) or the lack of ta product (entries 2–3, Table 1) were observed. Introduction of NaOH instead of TEA as a base, keeping the same reaction conditions as in entry 2 (without activation of DMAP), also gave a negative result (entry 4, Table 1). The reaction with *N,N*-diisopropylethylamine (DIPEA, entry 5, Table 1) or sodium hydride (NaH, entry 6, Table 1) resulted in 44% and 70% yields, respectively. The best yield of 76% was achieved in an approach where the solvent was dimethylformamide (DMF) and TEA was used as a base (entry 7, Table 1). A reaction performed on a gram scale while applying the same conditions resulted in the yield increasing to 84% (entry 8, Table 1). Upscaled reactions with other chloroformates (entries 9–15, Table 1) also gave the desired products, i.e., disubstituted guanidines, in good-to-excellent yields of 35–94%. For comparison, Feichtinger et al. [8] reported the synthesis of similar guanidine derivatives bearing two Boc- or Cbz- groups with yields of 58% and 83%, respectively. The structures of compounds **1**–**7** were confirmed using NMR and MALDI-MS (Figures S6–S26).

The disubstituted guanidines (**1**–**7**, Scheme 1) were used in a one-pot reaction in step 2 (Scheme 1) for the guanidinylation of *N*^α^-Fmoc-Orn containing an unprotected *δ*-amino group. Step two, in general, involved the activation of disubstituted guanidines and then reaction with Fmoc-Orn. Two reaction mixtures were prepared. Mixture A was composed of guanidine derivative (**1**–**7**), a base, Tf_2_O and solvent in 0 °C, while mixture B included Fmoc-Orn, a base, and a solvent in the room temperature. After 30 min of stirring, both mixtures were combined. Optimization of reaction conditions for step 2 is presented in Table 2. Initially, compound **1** (Scheme 1, Table 2) was combined with pyridine (Py, 2 eq.) and Tf_2_O (1.5 eq.) at 0 °C in dichloromethane (DCM) or dioxane to make mixture A (entries 1–5, Table 2). Mixture B comprised Fmoc-Orn (1 eq.), DIPEA or TEA in DCM/DMF or in dioxane at room temperature in various ratios (entries 1–5, Table 2). All reactions were performed at room temperature for 24 h and monitored with HPLC, but the desired product **1A** was not obtained. Further, the optimization was continued for compound **3** (entries 6–17, Table 2). Reactions were performed under similar conditions as in the case of **1** with modifications of temperature and solvent mixtures and the introduction of an additional solvent, namely toluene (entry 15, Table 2). The highest yield, although of only 8%, was achieved using Tf_2_O (1.5 eq.), Py (2 eq.), DCM, Fmoc-Orn (1 eq.), DIPEA (2 eq.) and DCM/DMF on rt/48 h (entry 10, Table 2) or using the same reagents and solvents but elevated temperature (40 °C) (entry 13, Table 2). Utilization of the same reaction conditions but with a shorter reaction time (5 h) and at room temperature resulted in a lower yield of 5% (entry 16, Table 2). Considering the small difference in the yield, we decided to use a shorter reaction time and the reaction conditions from entry 16 (Table 2) for synthesis of further Arg derivatives. Reactions with compounds **2** and **4**–**7** (Scheme 1) were performed under conditions applied successfully for compound **3** (i.e., entry 16, Table 2). Products **2A** and **4A** (Scheme 1) were isolated in 12% and 14% yield, respectively, and the structures of **2A**–**4A** were confirmed using NMR and MALDI-MS (Figures S27–S35). 

Products **1A** and **5A**–**7A** were not detected. Such divergent results which depended on the specific guanidynylating reagent, namely the length of an alkyl in the alkoxycarbonyl group, attracted our attention. We supposed that the reason lay in the amine reactivity or in guanidinylating reagents, i.e., **1**–**7**. Thus, we carried out reactions of **1**, **3** and **5** with *iso*-butylamine (p*K*_a_ = 10.31; p*K*_a_ (ornithine NH_2_ side chain) = 10.76), applying the conditions presented in Scheme 1. The reaction was successful in the cases of **3** and **5** and the signals of the desired products, i.e., guanidinylated *iso*-butylamine analogues, were found in both cases (Figures S49 and S50), while for **1** the desired product was not obtained. Additionally, for compounds **1** and **2,** NMR and computational analyses were performed to check if the length of the alkyl chain in the masking group may influence their reactivity (Figures S51–S68). The theoretical calculations (performed using the Gaussian16 program package) were carried out using Becke’s Three-Parameter Hybrid Method with the LYP (Lee–Yang–Parr) correlation functional (B3LYP) and the 6-31 + G* basis set. Exploration of the potential energy surfaces of **1** and **2** led to several locally geometrically stable isomers that differed from one another through mutual orientation of the -NH groups of the gauanidine-like fragment and—COOR groups (where R = C_2_H_5_, C_3_H_7_) and thus the resulting H-bond network. The hydrogen bonds formed between the -NH and carboxyl groups seem to be crucial for the stability of the identified isomers whose relative energies (with respect to the global minimum) were found to be within 15 kcal/mol. Due to the fact that two types of isomers (containing either one or two hydrogen atoms of the gauanidine fragment involved in the formation of N-H∙∙∙O hydrogen bonds) were recognized for each system, the rearrangement processes between the representative higher-energy isomer (comprising one N-H∙∙∙O hydrogen bond) and the global minimum structure were investigated. As it turned out, the isomerization pathways for **1** and **2** are very similar and require overcoming two kinetic barriers whose heights were predicted to be 1 and 14 kcal/mol in both cases. Thus, the results of our theoretical calculations seem to be inconclusive with respect to the relative reactivity of these two compounds, and the determination of the unsuccessful reaction of **1** with Orn requires deeper analysis, which is out of the scope of this paper.

**Figure 3:**
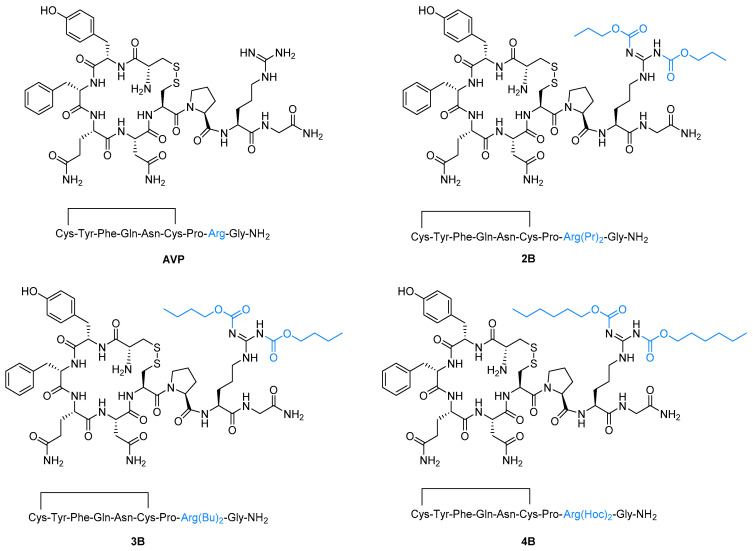
Structure of AVP and its analogues. Pr = propyloxycarbonyl; Bu = butyloxycarbonyl; Hoc = hexyloxycarbonyl.

Importantly, to prove the applicability of the obtained Arg derivatives in SPPS, we synthesized AVP prodrugs (**2B**–**4B**, Figure 3) using Fmoc chemistry procedures that are routinely used in our laboratory (Section 3.4 in Section Materials and Methods and Section S2 in Supplementary Materials. The Fmoc-protected building blocks **2A**–**4A** were introduced in place of Arg8. A disulfide bridge between Cys residues was formed in a methanolic solution using iodine. Pure peptides were obtained in a yield of 38–55% and were fully characterized (Figures S37–S48). It is important to underline that the purity of the crude prodrugs was comparable to that obtained for unmodified AVP. The permeability of the obtained prodrugs will be examined and compared to the mother peptide (AVP) and the results will be published as a separate paper.

## 3. Materials and Methods

### 3.1. General

All chemicals and solvents used were analytical- or HPLC-grade and commercially available. Reactions were monitored using thin-layer chromatography (TLC) on silica gel plates (Silica gel 60 F_254_, Merck, Darmstadt, Germany) and by reverse-phase high-performance liquid chromatography (RP-HPLC) on HPLC Pro Star System Varian (Mulgrave, Victoria, Australia) 330 with UV-Vis detector. The HPLC was performed on analytical column Aeris PEPTIDE XB-C18 3.6 μm, 100 × 4.6 mm (Phenomenex, Torrance, CA, USA). The linear gradient from 10% to 90% of phase B for 40 min, flow rate of 1 mL/min and detection at 226 nm were used. The solvent systems were 0.1% TFA in water (A) and 80% acetonitrile in A (B). The compounds were detected on TLC plates under UV light at 254 nm and with solution of potassium permanganate (solution of potassium permanganate, potassium carbonate, water and 10% sodium hydroxide). The products were purified via silica gel column chromatography on silica gel 60 (0.040–0.063 mm) (Milipore, Darmstadt, Germany). Determination and characterization of product structure was carried out via nuclear magnetic resonance (NMR), matrix-assisted laser desorption/ionization mass spectrometry (MALDI-MS) and/or electrospray ionization mass spectrometry (ESI-MS) and HPLC. The NMR spectra were recorded on a Bruker Avance III 500 MHz under the frequencies of 500 MHz (^1^H) and 126 MHz (^13^C) in CD_3_OD or CDCl_3_ in room temperature. The spectra were processed in the programs MestReNova version 0.2-5475, Mestrelab Research S,L; 2009 and TopSpin version 3.6.3. Chemical shifts (*δ*) are expressed according to deuterium solvent in ppm values, and coupling constants (*J*) are expressed in hertz (Hz). The ^1^H NMR spectra are shown as *δ* chemical shift/ppm (assignation, multiplicity, coupling constant, proton number). ^13^C NMR spectra are shown as *δ* chemical shift / ppm. The peaks are marked as s (singlet), t (triplet) or m (multiplet). The NMR spectra were analyzed according to one-dimensional (^1^H and ^13^C) and two-dimensional (Correlation Spectroscopy (COSY) and Heteronuclear Multiple-Quantum Correlation (HMQC)). MALDI-MS analysis was recorded on a Matrix-Assisted Laser Desorption/Ionization—time-of-flight (MALDI-TOF/TOF), Autoflex MAX spectrophotometer (Bruker, Billerica, MA, USA) on *α*-cyano-4-hydroxycinnamic acid (CCA) and/or 2,5-dihydroxybenzoic acid (DHB) matrix. ESI-MS was recorded on QTOF 5600+ (Sciex, Framingham, MA, USA) device. AVP analogues (**2B**–**4B**) were purified via PLC 2050 Gilson with Gilson Glider Prep. Software, Prep. Win. v. 5.1 (Middleton, USA equipped with a Grace Vydac C18 (218TP) HPLC column (22 × 250 mm, 10 μm, 300 Å, Resolution Systems). The solvent systems were 0.1% TFA in water (A) and 80% acetonitrile in A (B). Melting point (*m*_p_) was determined on Stuart melting point SMP3 apparatus, version 5.0. Compound names were generated by ChemDraw Professional (version 15.0.0.106) which follows IUPAC conventions.

### 3.2. General Procedure for the Synthesis of Dicarbamates ***1***–***7***

Guanidine hydrochloride (0.01 mol, 1 g) was dissolved in DMF (20 mL) and TEA (0.04 mol, 5.58 mL) was added. The reaction mixture was cooled to 0 °C and appropriate chloroformate (2.2 eq.) was added dropwise. The reaction mixture was stirred for 48 h at room temperature and solvent was removed under reduced pressure. pH was adjusted to 6 using 5M HCl, the product was extracted with ethyl acetate and the organic layer was washed with brine and dried over MgSO_4_. The solvent was removed under reduced pressure and the product was obtained via lyophilization. Characterization of the obtained compounds, including NMR and HRMS analyses, is given in Supplementary Materials. 

### 3.3. Synthesis of Arginine Derivatives ***2A***–***4A***

#### 3.3.1. (S,E)-N^2^-(((9H-fluoren-9-yl)methoxy)carbonyl)-Nω,Nω′-bis(propoxycarbonyl)-l-arginine (**2A**)

Compound **2** (2.16 mmol, 500 mg) was dissolved in DCM (23.5 mL) at 0 °C. Pyridine (Py; 4.32 mmol, 348 μL), and after 5 min, Tf_2_O (3.24 mmol, 544 μL), were added and the reaction mixture was stirred for 30 min. The second reaction mixture was Fmoc-Orn (2.16 mmol, 766 mg) dissolved in DCM/DMF (23.5 mL/11 mL) with the addition of DIPEA (4.32 mmol, 753 μL). The reaction mixture was stirred for 30 min at rt. The first solution was added to the second and the reaction mixture was stirred for 2 h at rt. The solvent was evaporated, the product was extracted with EtOAc/2M KHSO_4_ and the organic layer was washed with brine and dried over the MgSO_4_. The solvent was removed under reduced pressure. The product was purified with silica gel column chromatography in hexane/EtOAc/acetic acid (6:3:0.5) and was obtained after lyophilization as a white solid in 12% (0.271 mmol, 154 mg), *m*_p_ = 67–80; ^1^H NMR (CDCl_3_, 500 MHz): *δ* 8.72–8.43 (m, 1H), 7.77–7.74 (m, 2H), 7.62–7.54 (m, 2H), 7.41–7.36 (m, 2H), 7.32–7.28 (m, 2H), 5.94–5.68 (m, 2H), 4.56–4.35 (m, 3H), 4.22 (t, *J* = 6.9 Hz, 1H), 4.14–4.08 (m, 2H), 4.07–4.02 (m, 2H), 3.55–3.32 (m, 2H), 2.02–1.59 (m, 8H), 0.98–0.91 (m, 6H); ^13^C NMR (CDCl_3_, 126 MHz): *δ* 156.3, 155.8, 154.2, 144.0, 143.9, 141.4, 127.8, 127.2, 125.3, 120.1, 68.7, 67.8, 67.2, 53.5, 47.3, 40.9, 29.6, 25.2, 22.2, 21.9, 10.4, 10.3; MALDI-MS: calculated for C_29_H_36_N_4_O_8_ ([M + H]^+^) 569.258 and ([M + Na]^+^) 591.240; found 569.211 and 591.175.

#### 3.3.2. (S,E)-N^2^-(((9H-fluoren-9-yl)methoxy)carbonyl)-Nω,Nω′-bis(butoxycarbonyl)-l-arginine (**3A**)

Compound **3** (1.93 mmol, 500 mg) was dissolved in DCM (23.5 mL) at 0 °C. Pyridine (3.86 mmol, 310 μL), and after 5 min, Tf_2_O (2.89 mmol, 486 μL), were added and the reaction mixture was stirred for 30 min. The second reaction mixture was Fmoc-Orn (1.93 mmol, 684 mg) dissolved in DCM/DMF (18.5 mL/16 mL) with the addition of DIPEA (3.86 mmol, 672 μL). The reaction mixture was stirred for 30 min at rt. The first solution was added to the second solution and the reaction mixture was stirred for 2 h at rt. A new portion of compound **3** with Py (2 eq.) and Tf_2_O (1.5 eq.) were added and the reaction mixture was stirred for another 3 h. The solvent was evaporated, the product was extracted with EtOAc/2M KHSO_4_ and the organic layer was washed with brine and dried over MgSO_4_. The solvent was removed under reduced pressure. The product was purified on silica gel column chromatography in hexane/EtOAc/acetic acid (6:3:0.5) and was obtained after lyophilization as a white solid in 5% (0.204 mmol, 122 mg), *m*_p_ = 51–67; ^1^H NMR (CDCl_3_, 500 MHz): *δ* 11.69 (s, 1H), 8.45–8.28 (m, 1H), 7.77–7.73 (m, 2H), 7.62–7.53 (m, 2H), 7.41–7.36 (m, 2H), 7.32–7.27 (m, 2H), 5.73–5.60 (m, 1H), 5.13 (s, 1H), 4.55–4.35 (m, 3H), 4.23–4.19 (m, 1H), 4.16–4.10 (m, 2H), 4.09–4.04 (m, 2H), 3.50–3.31 (m, 2H), 2.05–1.88 (m, 1H), 1.78–1.59 (m, 7H), 1.42–1.34 (m, 4H), 0.94–0.89 (m, 6H); ^13^C NMR (CDCl_3_, 126 MHz): *δ* 163.9, 156.2, 154.3, 143.9, 141.4, 127.8, 127.2, 125.3, 120.1, 67.2, 66.6, 65.6, 53.6, 47.3, 40.6, 30.9, 30.6, 29.7, 25.3, 19.2, 19.0, 13.9, 13.7; MALDI-MS: calculated for C_31_H_40_N_4_O_8_ ([M + H]^+^) 597.288 and ([M + Na]^+^) 619.270; found 597.252 and 619.230.

#### 3.3.3. (S,E)-N^2^-(((9H-fluoren-9-yl)methoxy)carbonyl)-Nω,Nω′-bis((hexyloxy)carbonyl)-l-arginine (**4A**)

Compound **4** (1.59 mmol, 500 mg) was dissolved in DCM (23.5 mL) at 0 °C. Pyridine (3.18 mmol, 256 μL), and, after 5 min, Tf_2_O (2.39 mmol, 400μL), were added and the reaction mixture was stirred for 30 min. The second reaction mixture was Fmoc-Orn (1.59 mmol, 563 mg) dissolved in DCM/DMF (18.5 mL/16 mL) with the addition of DIPEA (3.18 mmol, 553 μL). The reaction mixture was stirred for 30 min at rt. The first solution was added to the second solution and the reaction mixture was stirred for 2 h at rt. A new portion of compound **4** with Py (2 eq.) and Tf_2_O (1.5 eq.) were added and the reaction mixture was stirred for 24 h. The solvent was evaporated, the product was extracted with EtOAc/2M KHSO4 and the organic layer was washed with brine and dried over the MgSO_4_. The solvent was removed under reduced pressure. The product was purified on silica gel column chromatography in hexane/EtOAc/acetic acid (6:3:0.5) and was obtained after lyophilization as a white solid in 14% (0.457 mmol, 298 mg), *m*_p_ = 74–77; ^1^H NMR (CDCl_3_, 500 MHz): *δ* 11.66 (s, 1H), 8.64–8.33 (m, 1H), 7.80–7.71 (m, 2H), 7.63–7.53 (m, 2H), 7.42–7.35 (m, 2H), 7.34–7.28 (m, 2H), 5.89–5.67 (m, 1H), 4.57–4.39 (m, 3H), 4.22 (t, *J* = 6.8 Hz, 1H), 4.15–4.11 (m, 2H), 4.09–4.04 (m, 2H), 3.56–3.35 (m, 2H), 1.98 (s, 1H); 1.79–1.52 (m, 7H), 1.41–1.26 (m, 12H), 0.91–0.84 (m, 6H); ^13^C NMR (CDCl_3_, 126 MHz): *δ* 174.7, 156.4, 155.9, 154.2, 143.9, 143.8, 141.6, 127.9, 127.2, 125.3, 120.1, 67.2, 66.2, 53.5, 47.3, 40.8, 31.6, 31.5, 29.6, 28.9, 28.6, 25.6, 25.5, 25.3, 22.7, 22.6, 14.2, 14.1; MALDI-MS: calculated for C_35_H_48_N_4_O_8_ ([M + H]^+^) 653.358; found 653.346.

### 3.4. General Procedure for the Synthesis of Arginine Vasopressin Analogues ***2B***–***4B***

AVP analogues were synthesized manually via solid-phase peptide synthesis (SPPS) applying Fmoc chemistry using Fmoc-RINK-MBHA resin (loading 0.646 mmol/g, GL Biochem, Shanghai, China). All amino acid derivatives and coupling reagents were purchased from GL Biochem, Shanghai, China. Peptide chain was elongated in sequential cycles of deprotection and coupling. Deprotection was performed with 20% piperidine in DMF. Protected amino acid derivatives (3 eq. in relation to the resin loading) were used during the first coupling in one of the following mixtures: Fmoc-AA/HBTU/HOBt/DIPEA, molar ratio 1:1:1:2 or Fmoc-AA/TBTU/HOBt/DIPEA, molar ratio 1:1:1:2. 

The coupling was repeated if needed with 1.5 eq of the appropriate amino acid derivative. It is important to underline that coupling of **2A**–**4A** followed the standard procedure, was completed after three cycles of coupling and each cycle lasted 90 min. Couplings were followed by Kaiser [47] and/or chloranil tests [48] to confirm reaction completion. After the attachment of *N*-terminal Fmoc-Cys(Trt) and removal of the Fmoc-protecting group, the peptide was cleaved from the resin simultaneously with the side chain deprotection in a one-step procedure. Therefore, the dried peptidyl resin was suspended in the mixture of TFA/H_2_O/PhOH/TIPS (88:5:5:2) and stirred for 3 h at room temperature [49]. Subsequently, the disulfide bridge was formed using a 0.1 M methanolic iodine solution [50]. Characterization of the obtained compounds, including NMR, HPLC and MS analyses, is given in Supplementary Material.

## 4. Conclusions

In this paper, we described the synthesis of new guanidinylating compounds **1**–**7**, which have alkyloxycarbonyl extensions with various lengths (ethyl–dodecyl), in good-to-excellent yields of 35%–94%, respectively. Utilization of the obtained compounds was examined in the reaction with the side-chain amine of Fmoc-Orn for the preparation of new arginine building blocks (i.e., **1A**–**7A**). Compounds **2A**–**4A**, which possess propyl, butyl or hexyl extensions, were isolated in the yields of 5–14%, while the products **1A** and **5A**–**7A**, i.e., with ethyl, octyl, decyl or dodecyl extensions, were not detected. Their absence may be explained by several facts, such as the influence of alkyl chains in **1**–**7** on the structure reorganization and thus reactivity of compounds, poor reactivity of amine and/or guanidinylating compounds, or the existence of several conformational isomers which preclude or completely abolish the reaction with Orn. We are aware of the low yield obtained here for **2A**–**4A** even despite multiple struggles to optimize the reaction conditions. Importantly, the synthesis of such Fmoc-Arg derivatives with alkoxycarbonyl side-chain-protecting groups by applying different approaches was previously reported for only the hexyloxycarbonyl group. Unfortunately, no details regarding the yield and analyses, such as NMR or HPLC, were available, and thus we are not able to relate and compare the yields obtained in our work. Notably, the viability of the obtained Arg derivatives for SPPS was confirmed, as **2A**–**4A**, with propyl, butyl or hexyl extensions, were successfully utilized for the syntheses of arginine vasopressin prodrugs with increased lipophilicity, where Arg side chain charge is masked with two alkoxycarbonyl groups (**2B**–**4B**). 

## Figures and Tables

**Table 1:** Optimization of reaction conditions for step 1.

Entry	m (Guanidine Hydrochloride)/g	Base/eq.	Solvent/mL	t/°C	Chloroformate/2.2 eq.	*ƞ*/%
1	0.25	TEA/4	ACN/H_2_O 3/1	0→rt	butyl	13
2 ^a^	0.1	TEA/4	ACN/H_2_O 1.2/1.2	0→rt	butyl	-
3	0.1	TEA/4	ACN/H_2_O 1.2/1.2	40	butyl	-
4	0.1	1M NaOH	ACN/H_2_O 1.2/1.2	0→rt	butyl	-
5	0.1	DIPEA/4	ACN/H_2_O 1.2/1.2	0→rt	butyl	44
6	0.1	NaH/4	ACN/H_2_O 1.2/1.2	0→rt	butyl	70
7	0.1	TEA/4	DMF/2	0→rt	butyl	76
8	1	TEA/4	DMF/20	0→rt	butyl	84
9	2	TEA/4	DMF/40	0→rt	ethyl	23
10	1	TEA/4	DMF/80	0→rt	ethyl	35
11	1	TEA/4	DMF/20	0→rt	propyl	57
12	1	TEA/4	DMF/20	0→rt	hexyl	67
13	1	TEA/4	DMF/20	0→rt	octyl	70
14	1	TEA/4	DMF/20	0→rt	decyl	53
15	1	TEA/4	DMF/20	0→rt	dodecyl	94

^a^ Butyl chloroformate was activated with DMAP in ACN.

**Table 2:** Optimization of reaction conditions for step 2, Scheme 1.

	Mixture A				Mixture B				
Entry [Compound (1–7)/mmol] ^a^	Tf_2_O/eq.	Base/eq.	Solvent/mL	t/°C	Fmoc-Orn/eq.	Base/eq.	Solvent/mL	t/°C; t/h	*ƞ*/%
1 [**1**/2.461] ^b^	1.5	Py/2	DCM/23.5	0	1	DIPEA/2	DCM/DMF/23.5/11	rt; 24	-
2 [**1**/0.492] ^b^	1.5	Py/2	DCM/4.7	0	1	DIPEA/2	DCM/DMF/4.7/2.2	rt; 24	-
3 [**1**/1.058] ^c^	1.5	Py/2	DCM/10	0	1	DIPEA/2	DCM/DMF/10/4.7	rt; 24	-
4 [**1**/0.246] ^b^	1.5	Py/2	DCM/2.4	0	1	DIPEA/2	DCM/DMF/2.4/1.1	rt; 24	-
5 [**1**/1.826]	1.5	Py/1.2	dioxane/7	0	0.2	TEA/1.2	dioxane/5	rt; 24	-
6 [**3**/0.157]	1.5	Py/2	DCM/1.4	rt	1	DIPEA/1.5	DCM/DMF/1.4/0.7	rt; 24	-
7 [**3**/0.104] ^d^	1	NaH/2	H_2_O/ACN/0.1/0.1	−45	-	-	-	rt; 24	-
8 [**3**/0.096]	1.5	Py/2	DCM/1.3	0	1	DIPEA/1.5	DCM/DMF/1.3/0.5	rt; 3	-
9 [**3**/0.096]	1.5	Py/2	DCM/1.3	0	1	DIPEA/1.5	DCM/DMF/1.3/0.5	40; 2	-
10 [**3**/0.386] ^c,e^	1.5	Py/2	DCM/4.7	0	1	DIPEA/2	DCM/DMF/4.7/2.2	rt/; 8	8
11 [**3**/0.386] ^c,e^	1.5	Py/2	DCM/4.7	−78	1	DIPEA/2	DCM/DMF/4.7/2.2	0→rt; 96	3
12 [**3**/0.386] ^b,e^	1.5	Py/2	DCM/4.7	0	1	DIPEA/2	DCM/DMF/4.7/2.2	40; 24	6
13 [**3**/0.386] ^b,e^	1.5	Py/2	DCM/4.7	0	1	DIPEA/2	DCM/DMF/3.7/3.2	40; 24	8
14 [**3**/0.386] ^b,e^	1.5	Py/2	dioxane/4.7	0	1	DIPEA/2	dioxane/DMF/4.7/2.2	65; 24	-
15 [**3**/0.386] ^a,c^	1.5	Py/2	toluene/4.7	0	1	DIPEA/2	toluene/DMF/4.7/1	90; 5	1
16 [**3**/1.928] ^b,e^	1.5	Py/2	DCM/23.5	0	1	DIPEA/2	DCM/DMF/18.5/16	rt; 5	5
17 [**3**/1.928] ^b,e^	1.5	Py/2	DCM/23.5	0	1	DIPEA/2	DCM/DMF/18.5/16	rt; 5	-
18 [**4**/0.317]	1.5	Py/2	DCM/4.5	0	1	DIPEA/2	DCM/DMF/4.5/2.1	rt; 24	4
19 [**4**/0.793] ^b^	1.5	Py/2	DCM/12	0	1	DIPEA/2	DCM/DMF/12/5.4	rt; 24	4
20 [**4**/1.585] ^b,e^	1.5	Py/2	DCM/23.5	0	1	DIPEA/2	DCM/DMF/23.5/11	rt; 24	14
21 [**4**/0.317] ^d,f^		TEA/2.2					DMF/0.50	rt; 24	-
22 [**4**/0.317] ^d,f^		TEA/2.2					DCM/3.5	rt; 24	-
23 [**4**/0.159] ^d,f^		DIPEA/2.2					DCM/DMF0.3/0.1	rt; 24	-
24 [**5**/0.269] ^e^	1.5	Py/2	DCM/4.7	0	1	DIPEA/2	DCM/DMF/4.7/3.2	rt; 24	-
25 [**5**/0.269] ^e^	1	NaH/3.2	DCM/3	0	-	-	-	-	-
26 [**6**/0.234] ^e^	1	Py/2	DCM/5	0	1	DIPEA/2	DCM/DMF/5/3	rt; 24	-
27 [**7**/0.207] ^e^	1.5	Py/2	DCM/10	0	1	DIPEA/2	DCM/DMF/10/4	rt; 24	-

^a^ Reaction mixtures were stirred at room temperature; ^b^ new portion of reaction mixture A was added after 2 h of stirring; ^c^ new portion of reaction mixture A was added after 2 h and 4 h of stirring; ^d^ reaction was performed only with mixture A in order to examine the very low reaction temperature of formation TfO-Gu(Bu)_2_. ^e^ In the reaction, fresh Tf_2_O (new bottle) was used; ^f^ Mukayama reagent was used.

## Data Availability

Data is contained within the article or supplementary material.

## Supplementary Materials

The following Supplementary Materials can be downloaded at: https://www.mdpi.com/article/10.3390/molecules28237780/s1, which provide the analysis of prepared compounds, and copies of ^1^H and ^13^C NMR spectra.
