# Supplementary material for: Synthesis of Novel Arginine Building Blocks with Increased Lipophilicity Compatible with Solid-Phase Peptide Synthesis
Source: Molecules. 2023 Nov 25;28(23):7780. doi: 10.3390/molecules28237780 (PMC10708530; doi:10.3390/molecules28237780)
Supplement: Supplementary file 1 [file molecules-28-07780-s001.zip › molecules-2709069-supplementary.pdf]

Supplementary Materials

for

Synthesis of Novel Arginine Building Blocks with Increased Lipophilicity Compatible with Solid-Phase Peptide Synthesis

Mladena Glavaš[1,2], Agata Gitlin-Domagalska[1*], Natalia Ptaszyńska[1], Dominika Starego[1], Sylwia Freza[3], Dawid Dębowski[1], Aleksandra Maciejewska[1], Anna Łęgowska[1], Chaim Gilon[4], and Krzysztof Rolka[1]

[1] Department of Molecular Biochemistry, Faculty of Chemistry, University of Gdansk, Wita Stwosza 63, 80-308 Gdansk, Poland; mglavas@irb.hr (M.G.); natalia.ptaszynska@ug.edu.pl (N.P.); dominika.starego@phdstud.ug.edu.pl (D.S.); dawid.debowski@ug.edu.pl (D.D.); aleksandra.maciejewska@phdstud.ug.edu.pl (A.H.-M.); anna.legowska@ug.edu.pl (A.Ł.); krzysztof.rolka@ug.edu.pl (K.R.)

[2] Department of Organic Chemistry and Biochemistry, Ruđer Bošković Institute, Bijenička cesta 54, 10 000 Zagreb, Croatia; mglavas@irb.hr (M.G.)

[3] Department of Theoretical Chemistry, Faculty of Chemistry, University of Gdansk, Wita Stwosza 63, 80-308 Gdansk, Poland; sylwia.freza@ug.edu.pl (S.F.)

[4] Department of Organic Chemistry, Institute of Chemistry, The Hebrew University, Jerusalem 91904, Israel; chaimgilon@gmail.com (C.G.)

* Correspondence: agata.domagalska@ug.edu.pl

Content

1. Characterization of dicarbamates 1 - 7 1

1.1. Diethyl(iminomethylene)dicarbamate (1) 1

1.2. Dipropyl(iminomethylene)dicarbamate (2) 2

1.3. Dibutyl(iminomethylene)dicarbamate (3) 2

1.4. Dihexyl(iminomethylene)dicarbamate (4) 2

1.5. Dioctyl(iminomethylene)dicarbamate (5) 2

1.6. Didecyl(iminomethylene)dicarbamate (6) 2

1.7. Didodecyl(iminomethylene)dicarbamate (7) 2

2. Characterization of AVP-derivatives 2B – 4B 2

2.1. AVP(Pr)$_2$ (2B) 2

2.2. AVP(Bu)$_2$ (3B) 3

2.3 AVP(Hoc)$_2$ (4B) 3

3. Snythesis of Arg-derivatives 1A – 7A using of Fmoc-Arg-OMe as a starting compound 3

4. NMR, MALDI-MS, ESI-MS and HPLC spectra 5

4.1. Reaction of 3 and 5 with iso-butylamine 35

4.2. NMR comparison of compounds 1 and 2 37

1. Characterization of dicarbamates **1** - **7**

*1.1. Diethyl(iminomethylene)dicarbamate (**1**)*

35 % (3.53 mmol, 0.718 g), white solid, m_P = 85 – 108; ^{1}H NMR (CD_3OD, 500 MHz): δ 4.19 – 4.15 (m, 4H), 1.30 – 1.27 (m, 6H); ^{13}C NMR (CD_3OD, 126 MHz): δ 160.7, 62.8, 14.6; MALDI-MS: calculated for $C_7H_{13}N_3O_4$ ([M + H]$^+$) 204.098 and ([M + Na]$^+$) 226.080; found 204.175 and 226.152.

*1.2. Dipropyl(iminomethylene)dicarbamate (**2**)*

57 % (5.71 mmol, 1.32 g), white solid, m_P = 83 – 90; ^{1}H NMR (CD_3OD, 500 MHz): δ 4.10 – 4.07 (m, 4H), 1.72 – 1.65 (m, 4H), 0.97 – 0.96 (m, 6H); ^{13}C NMR (CD_3OD, 126 MHz): δ 160.8, 68.4, 23.2, 10.6; MALDI-MS: calculated for $C_9H_{17}N_3O_4$ ([M + H]$^+$) 232.128 and ([M + Na]$^+$) 254.110; found 232.374 and 254.331.

*1.3. Dibutyl(iminomethylene)dicarbamate (**3**)*

84 % (8.37 mmol, 2.17 g), pale yellow solid, m_P = 67 – 90; ^{1}H NMR (CD_3OD, 500 MHz): δ 4.32 – 4.29 (m, 4H), 1.74 – 1.69 (m, 4H), 1.48 – 1.41 (m, 4H), 0.99 – 0.96 (m, 6H); ^{13}C NMR (CD_3OD, 126 MHz): δ 155.9, 154.6, 68.9, 31.5, 19.8, 13.9; MALDI-MS: calculated for $C_{11}H_{21}N_3O_4$ ([M + H]$^+$) 260.158; found 260.008.

*1.4. Dihexyl(iminomethylene)dicarbamate (**4**)*

67 % (6.66 mmol, 2.10 g), pale yellow solid, m_P = 62 – 101; ^{1}H NMR (CD_3OD, 500 MHz): δ 4.27 – 4.24 (m, 2H), 4.13 – 4.11 (m, 2H), 1.74 – 1.63 (m, 4H), 1.43 – 1.33 (m, 12H), 0.94 – 0.90 (m, 6H); ^{13}C NMR (CD_3OD, 126 MHz): δ 68.4, 66.9, 32.6, 32.5, 29.9, 29.6, 26.6, 26.5, 23.6, 14.3; MALDI-MS: calculated for $C_{15}H_{29}N_3O_4$ ([M + H]$^+$) 316.228 and ([M + Na]$^+$) 338.210; found 316.170 and 338.149.

*1.5. Dioctyl(iminomethylene)dicarbamate (**5**)*

70 % (6.99 mmol, 2.60 g), white solid, m_P = 62 – 73; ^{1}H NMR ($CDCl_3$, 500 MHz): δ 8.49 (s, 1H), 8.28 (s, 1H), 4.24 – 4.15 (m, 4H), 1.71 – 1.64 (m, 4H), 1.38 – 1.24 (m, 20H), 0.90 – 0.85 (m, 6H); ^{13}C NMR ($CDCl_3$, 126 MHz): δ 157.5, 156.6, 153.2, 67.9, 67.3, 31.9, 29.3, 29.2, 28.7, 28.5, 25.8, 25.6, 22.8, 14.2; MALDI-MS: calculated for $C_{19}H_{37}N_3O_4$ ([M + H]$^+$) 372.228; found 372.279.

*1.6. Didecyl(iminomethylene)dicarbamate (**6**)*

53 % (5.31 mmol, 2.27 g), white solid, m_P = 44 – 62; ^{1}H NMR ($CDCl_3$, 500 MHz): δ 8.60 (s, 1H), 8.30 (s, 1H), 4.25 – 4.09 and 3.65 – 3.62 (m, 4H), 1.70 – 1.54 (m, 4H), 1.35 – 1.23 (m, 28H), 0.90 – 0.85 (m, 6H); ^{13}C NMR ($CDCl_3$, 126 MHz): δ 158.2, 156.5, 153.4, 67.9, 66.6, 63.2, 32.9, 32.0, 29.7, 29.4, 29.3, 28.8, 28.6, 25.9, 25.6, 22.8, 14.2; MALDI-MS: calculated for $C_{23}H_{45}N_3O_4$ ([M + H]$^+$) 428.348; found 428.355.

*1.7. Didodecyl(iminomethylene)dicarbamate (**7**)*

94 % (9.45 mmol, 4.57 g), white solid, m_P = 69 – 92; ^{1}H NMR ($CDCl_3$, 500 MHz): δ 9.02 – 8.01 (m, 1H), 4.25 – 4.09 and 3.65 – 3.61 (m, 4H), 1.70 – 1.53 (m, 4H), 1.36 – 1.24 (m, 36H), 0.90 – 0.85 (m, 6H); ^{13}C NMR ($CDCl_3$, 126 MHz): δ 157.9, 68.2, 66.8, 63.2, 32.9, 32.1, 29.8, 29.7, 29.6, 29.5, 29.4, 28.8, 28.7, 25.9, 22.8, 14.2; MALDI-MS: calculated for $C_{27}H_{53}N_3O_4$ ([M + H]$^+$) 484.408; found 484.388.

2. Characterization of AVP-derivatives **2B** – **4B**

*2.1. AVP(Pr)$_2$ (**2B**)*

23 % (0.075 mmol, 94 mg), white solid; ^{1}H NMR (CD_3OD, 500 MHz): δ 7.25 – 7.14 (m, 3H), 7.10 – 6.98 (m, 4H), 6.74 – 6.65 (m, 2H), 5.95 – 4.89 (m, 1H), 4.59 (t, *J* = 7.1 Hz, 1H), 4.39 – 4.33 (m, 1H), 4.28 – 4.19 (m, 2H), 4.18 – 4.12 (m, 2H), 4.10 – 4.01 (m, 3H), 3.97 – 3.92 (m, 1H), 3.91 – 3.82 (m, 1H), 3.82 – 3.71 (m, 2H), 3.70 – 3.63 (m, 1H), 3.49 – 3.37 (m, 3H), 3.23 – 3.10 (m, 4H), 3.07 – 2.95 (m, 3H), 2.92 – 2.86 (m, 1H), 2.82 – 2.73 (m, 2H), 2.29 – 2.12 (m, 4H), 2.10 – 1.88 (m, 5H), 1.79 – 1.62 (m, 7H), 0.98 – 0.89 (m, 6H); ^{13}C NMR (CD_3OD, 126 MHz): δ 178.2, 174.9, 174.3, 173.6, 170.6, 154.7, 131.7, 130.3, 129.7, 128.6, 127.8, 116.4, 69.8, 69.1, 62.3, 58.9, 56.3, 54.7, 54.0, 52.5, 51.4, 43.3, 42.1, 41.0, 38.6, 37.1, 32.6, 30.4, 29.1, 26.9, 26.4, 26.2, 23.2, 22.9, 10.7, 10.5; ESI-MS: calculated for $C_{54}H_{77}N_{15}O_{16}S_2$ [M+H$^+$] 1256.5194; found 1256.7073; HPLC 15.97 min, ClogP = 0.225.

*2.2. AVP(Bu)₂ (**3B**)*

22 % (0.036 mmol, 46.7 mg), white solid; ^{1}H NMR (CD_3OD, 500 MHz): *δ* 8.68 – 8.56 (m, 0.5H), 8.21 – 8.17 (m, 0.5H), 7.29 – 7.24 (m, 2H), 7.22 – 7.18 (m, 1H), 7.13 – 7.04 (m, 4H), 6.76 – 6.71 (m, 2H), 4.99 – 4.94 (m, 1H), 4.65 – 4.60 (m, 1H), 4.43 – 4.38 (m, 1H), 4.31 – 4.20 (m, 4H), 4.20 – 4.14 (m, 2H), 4.11 – 4.07 (m, 1H), 4.04 – 3.97 (m, 1H), 3.94 – 3.88 (m, 1H), 3.86 – 3.77 (m, 2H), 3.75 – 3.69 (m, 1H), 2.32 – 2.16 (m, 4H), 2.13 – 2.04 (m, 2H), 2.03 – 1.91 (m, 3H), 1.84 – 1.73 (m, 2H), 1.72 – 1.64 (m, 5H), 1.47 – 1.39 (m, 4H), 0.99 – 0.94 (m, 6H); ^{13}C NMR (CD_3OD, 126 MHz): *δ* 178.2, 174.8, 170.6, 157.6, 138.6, 131.7, 130.3, 129.7, 128.6, 127.8, 116.4, 68.3, 67.6, 62.3, 56.4, 54.7, 53.9, 43.2, 42.9, 42.3, 38.6, 37.1, 32.6, 31.9, 31.6, 30.4, 29.0, 26.9, 26.3, 26.2, 20.1, 19.9, 14.1; ESI-MS: calculated for $C_{56}H_{81}N_{15}O_{16}S_2$ [M+H$^+$] 1284.5507; found 1284.7415; HPLC 19.98 min. (method 10 – 90 of phase B/40 min); ClogP = 1.283.

*2.3 AVP(Hoc)₂ (**4B**)*

30 % (0.097 mmol, 130 mg), white solid; ^{1}H NMR (CD_3OD, 500 MHz): *δ* 7.28 – 7.24 (m, 2H), 7.22 – 7.18 (m, 1H), 7.14 – 7.09 (m, 2H), 7.07 – 7.04 (m, 2H), 6.76 – 6.71 (m, 2H), 4.98 – 4.93 (m, 1H), 4.63 (t, *J* = 7.3 Hz, 1H), 4.43 – 4.38 (m, 1H), 4.29 – 4.20 (m, 4H), 4.14 – 4.07 (m, 3H), 4.01 – 3.96 (m, 1H), 3.85 – 3.76 (m, 2H), 3.74 – 3.68 (m, 1H), 3.47 – 3.42 (m, 2H), 3.26 – 3.17 (m, 3H), 3.11 – 2.99 (m, 3H), 2.96 – 2.90 (m, 1H), 2.85 – 2.79 (m, 2H), 2.31 – 2.16 (m, 4H), 2.13 – 1.92 (m, 5H), 1.81 – 1.64 (m, 7H), 1.43 – 1.32 (M, 12H), 0.94 – 0.89 (m, 6H); ^{13}C NMR (CD_3OD, 126 MHz): *δ* 178.2, 174.8, 167.7, 157.6, 154.8, 138.6, 131.7, 130.3, 129.7, 128.6, 127.8, 116.4, 68.3, 67.4, 62.3, 58.9, 56.3, 54.8, 54.0, 52.5, 43.3, 42.9, 41.9, 38.6, 37.1, 32.7, 32.6, 30.4, 29.9, 29.6, 29.1, 26.9, 26.7, 26.5, 26.2, 23.6, 14.3; ESI-MS: calculated for $C_{60}H_{89}N_{15}O_{16}S_2$ [M+H$^+$] 1340.6133; found 1340.8142; HPLC 25.13 min. (method 10 – 90 of phase B/40 min); ClogP = 3.399.

3. Snythesis of Arg-derivatives **1A – 7A** using of Fmoc-Arg-OMe as a starting compound

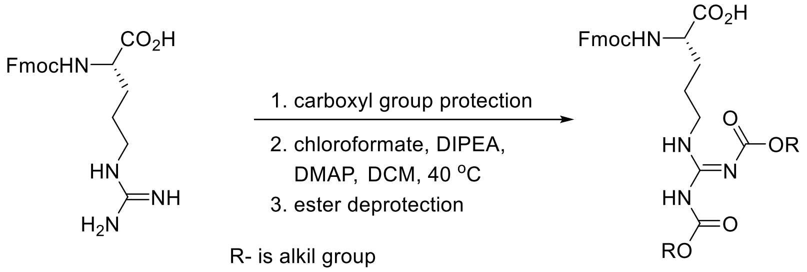

1A - 7A

Scheme S1. Synthesis of Arg-derivatives **1A – 7A** through three steps, starting from Fmoc-Arg

Table S1. Optimization of reaction conditions for the side chain chloroformate protection.

Entry	n (FmocArgOMe)/mmol	base/eq.	chloroformate/eq.	solvent/mL	t/°C; t/h	η/%[e]

1	0.085[a]	DIPEA/6.5	ethyl/6.5	DCM/1.4	rt; 28	49
2	0.073[b]	DIPEA/4.5	ethyl/4.5	DCM/1.2	rt; 72	36
3	0.085[c]	DIPEA/4.5	ethyl/4.5	DMF/1.4	rt; 72	-
4	0.085[c,d]	DIPEA/4.5	ethyl/4.5	DCM/1.4	rt; 24	< 10
5	0.080	DIPEA/6.5	ethyl/6.5	DCM/1.3	40; 24	70
6	0.244[c,d]	DIPEA/6.5	ethyl/6.5	DCM/4	40; 24	68
7	0.244[c,d]	DIPEA/6.5	ethyl/6.5	DCM/4	40; 48	44
8	2.44[c,d]	DIPEA/6.5	ethyl/6.5	DCM/40	40; 24	88

[a]The reaction started with 2.5 eq. of base and chloroformate. After 2h of stirring was added 2.5 eq., additionally and after 24h of stirring 1.5 eq.; [b]After every 1h of stirring was added 1.5 eq. of base and chloroformate; [c]The reaction started with 2.5 eq. of base and chloroformate. After 2h of stirring was added 2.5 eq., additionally and after 24h of stirring 1.5 eq.; [d]Catalytic amount of DMAP was used; [e]The yield is calculated according to both isomers.

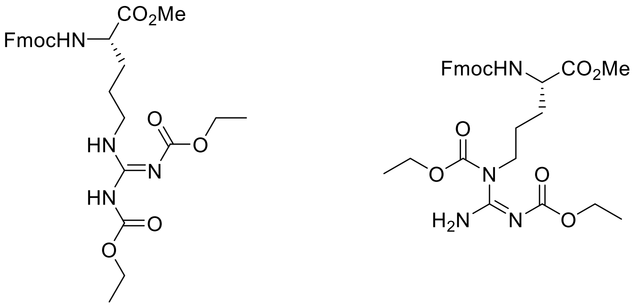

1C **1D**

Figure S1. Structure of compounds obtained in the reaction of Fmoc-Arg-OMe and ethyl chloroformate.

4. NMR, MALDI-MS, ESI-MS and HPLC spectra

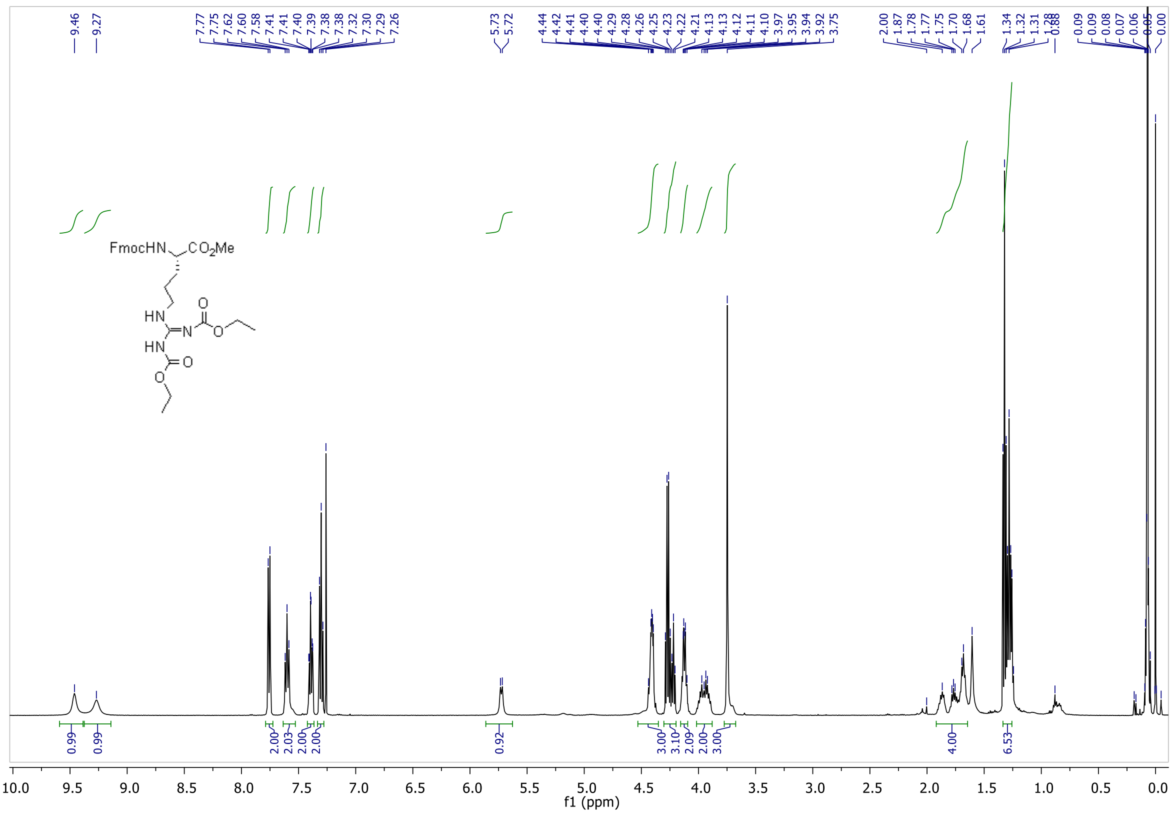

^{1}H NMR ($CDCl_3$, 500 MHz): δ 9.46 (s, 1H), 9.27 (s, 1H), 7.79 – 7.72 (m, 2H), 7.64 – 7.53 (m, 2H), 7.42 – 7.36 (m, 2H), 7.33 – 7.28 (m, 2H), 5.73 (d, *J* = 8.1 Hz, 1H), 4.53 – 4.35 (m, 3H), 4.30 – 4.20 (m, 3H), 4.16 – 4.09 (m, 2H), 4.02 – 3.88 (m, 2H), 3.75 (s, 3H), 1.92 – 1.65 (m, 4H), 1.34 – 1.24 (m, 6H).

Figure S2. ^{1}H NMR spectrum of **1C**.

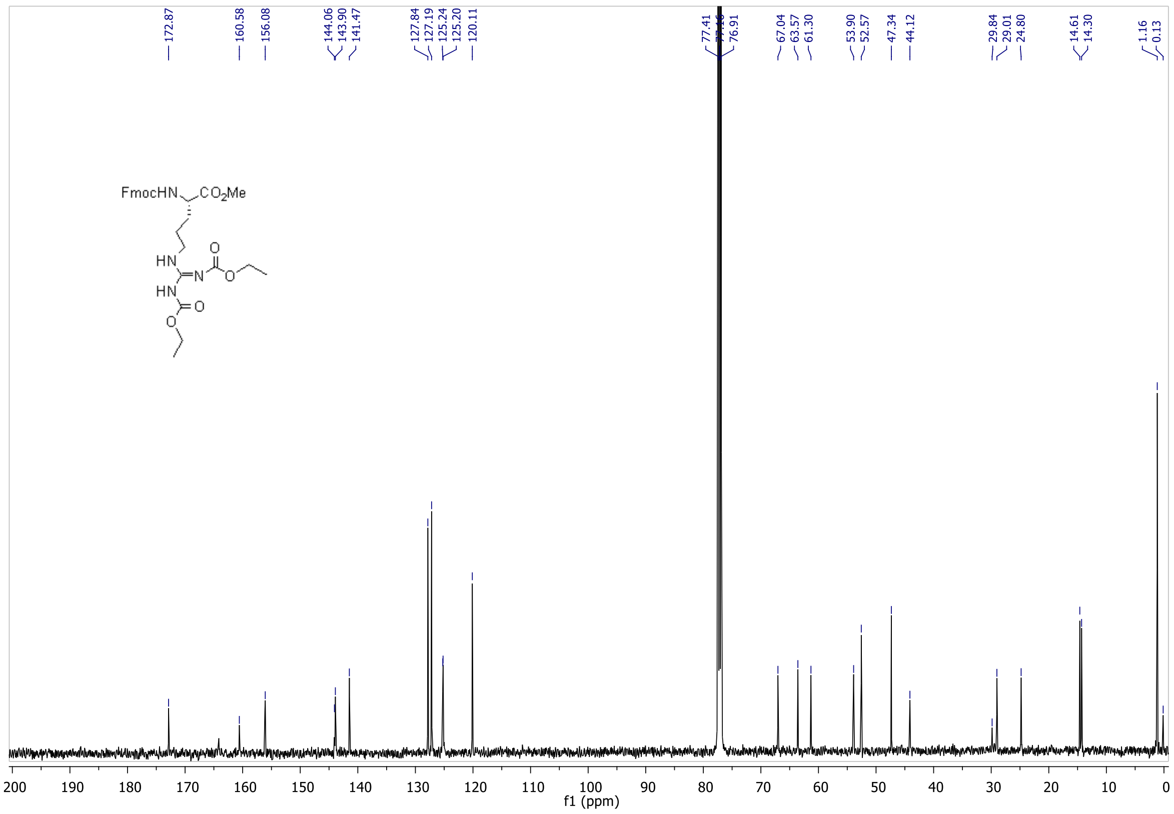

^{13}C NMR ($CDCl_3$, 126 MHz): *δ* 160.6, 156.1, 144.1, 143.9, 141.5, 127.8, 127.2, 125.2, 120.1, 63.6, 61.3, 53.9, 52.6, 47.3, 44.1, 29.0, 24.8, 14.6, 14.3.

Figure S3. ^{13}C NMR spectrum of **1C.**

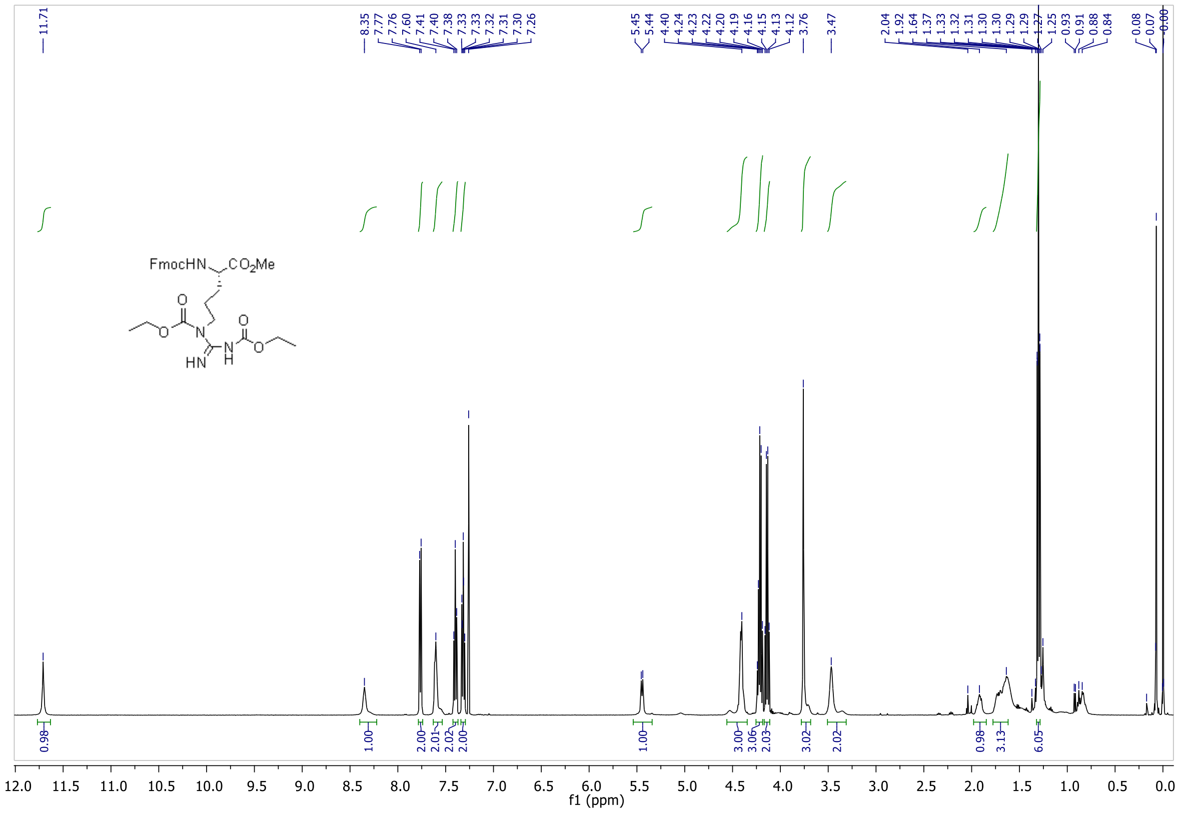

^{1}H NMR ($CDCl_3$, 500 MHz): δ 11.71 (s, 1H), 8.53 (s, 1H), 7.79 – 7.74 (m, 2H), 7.63 – 7.54 (m, 2H), 7.42 – 7.37 (m, 2H), 7.34 – 7.29 (m, 2H), 5.45 (d, J = 8.1 Hz, 1H), 4.56 – 4.35 (m, 3H), 4.25 – 4.18 (m, 3H), 4.17 – 4.11 (m, 2H), 3.76 (s, 3H), 3.51 – 3.31 (m, 2H), 1.98 – 1.85 (m, 1H), 1.78 – 1.62 (m, 3H), 1.32 – 1.28 (m, 6H).

Figure S4. ^{1}H NMR spectrum of **1D**.

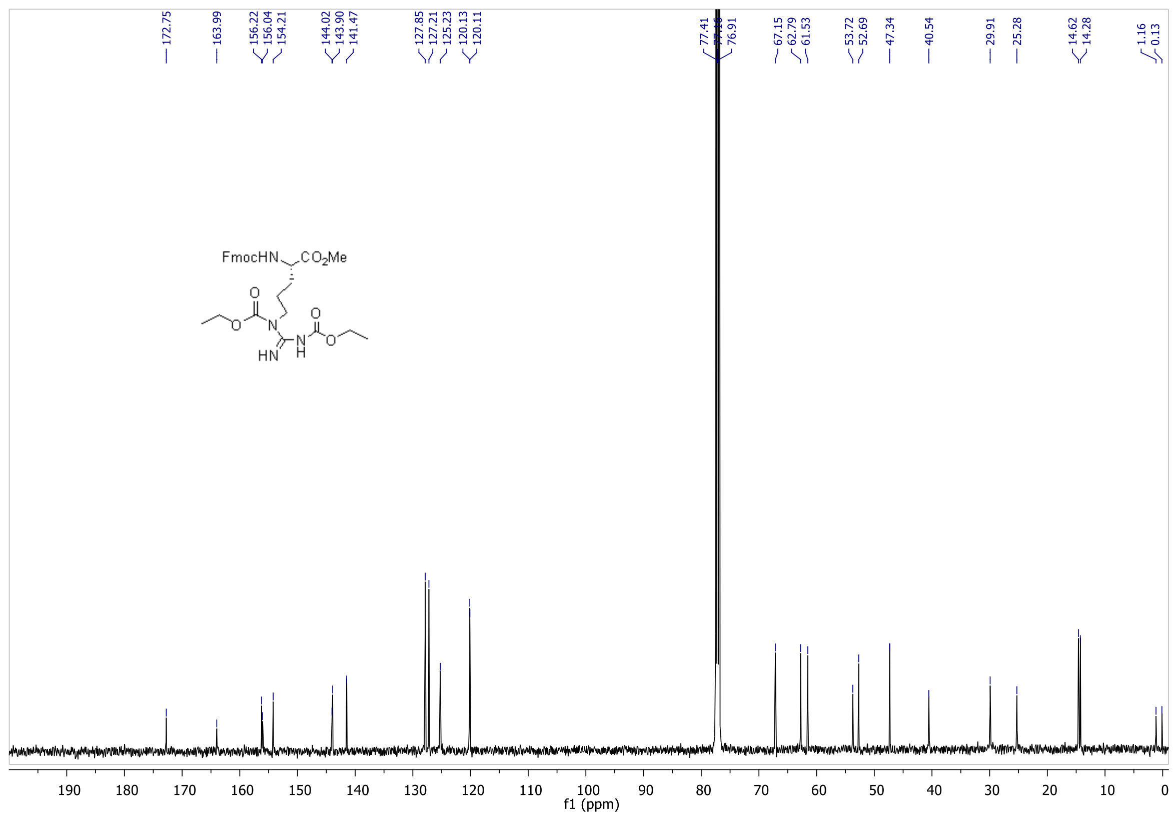

^{13}C NMR ($CDCl_3$, 126 MHz): δ 172.8, 163.9, 156.2, 156.0, 154.2, 144.0, 143.9, 141.5, 127.9, 127.2, 125.2, 120.1, 67.2, 62.8, 61.5, 53.7, 52.7, 47.3, 40.5, 29.9, 25.3, 14.6, 14.3.

Figure S5. ^{13}C NMR spectrum of **1D.**

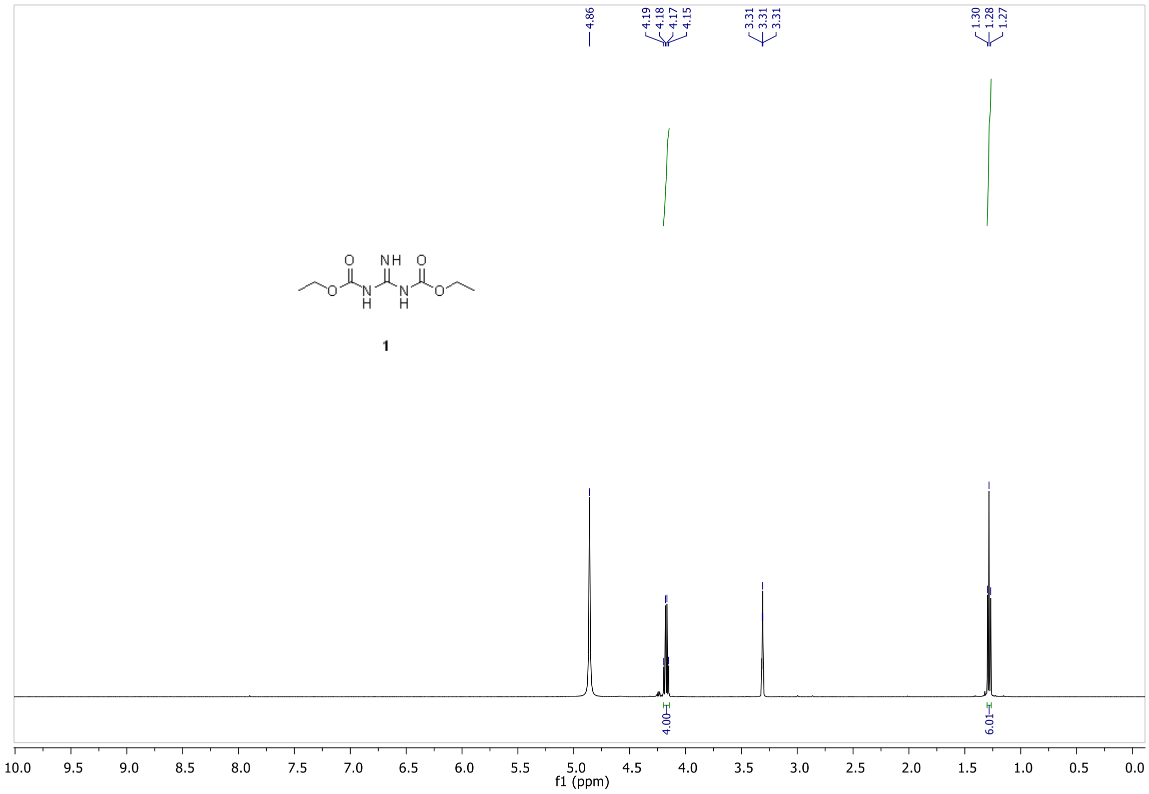

Figure S6. ^{1}H NMR spectrum of **1**.

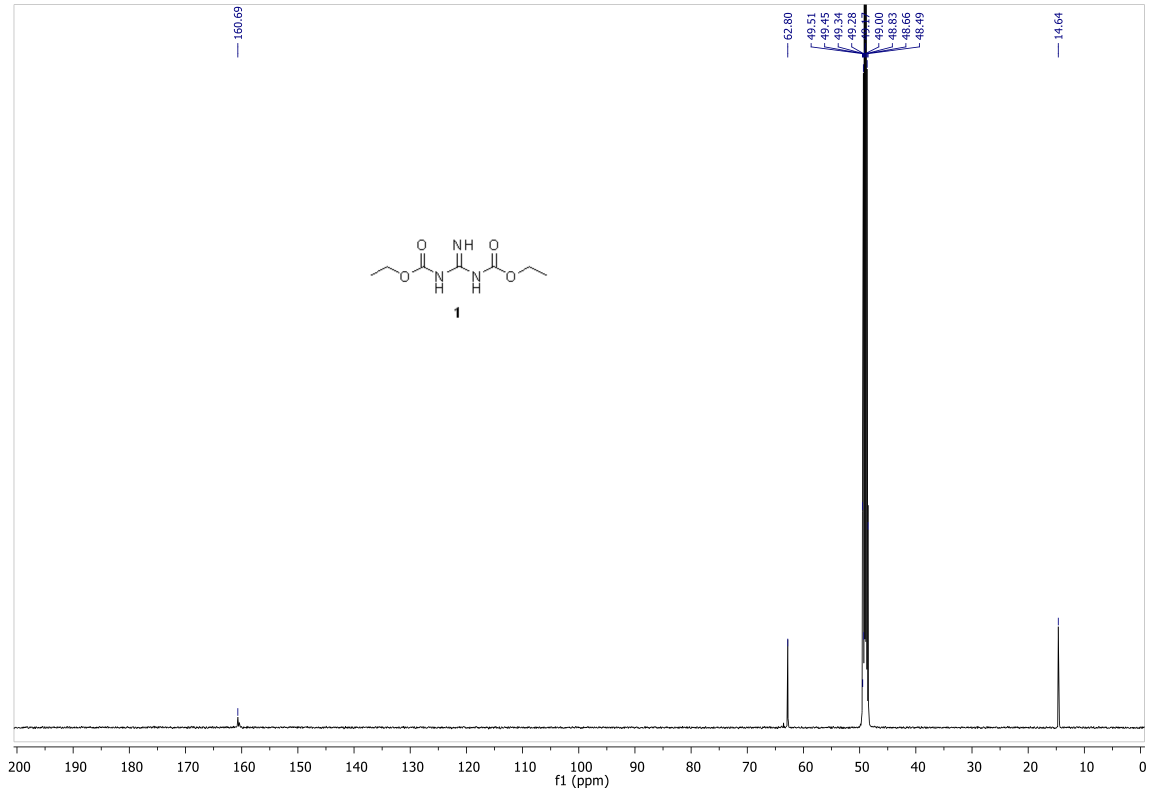

Figure S7. ^{13}C NMR spectrum of **1**.

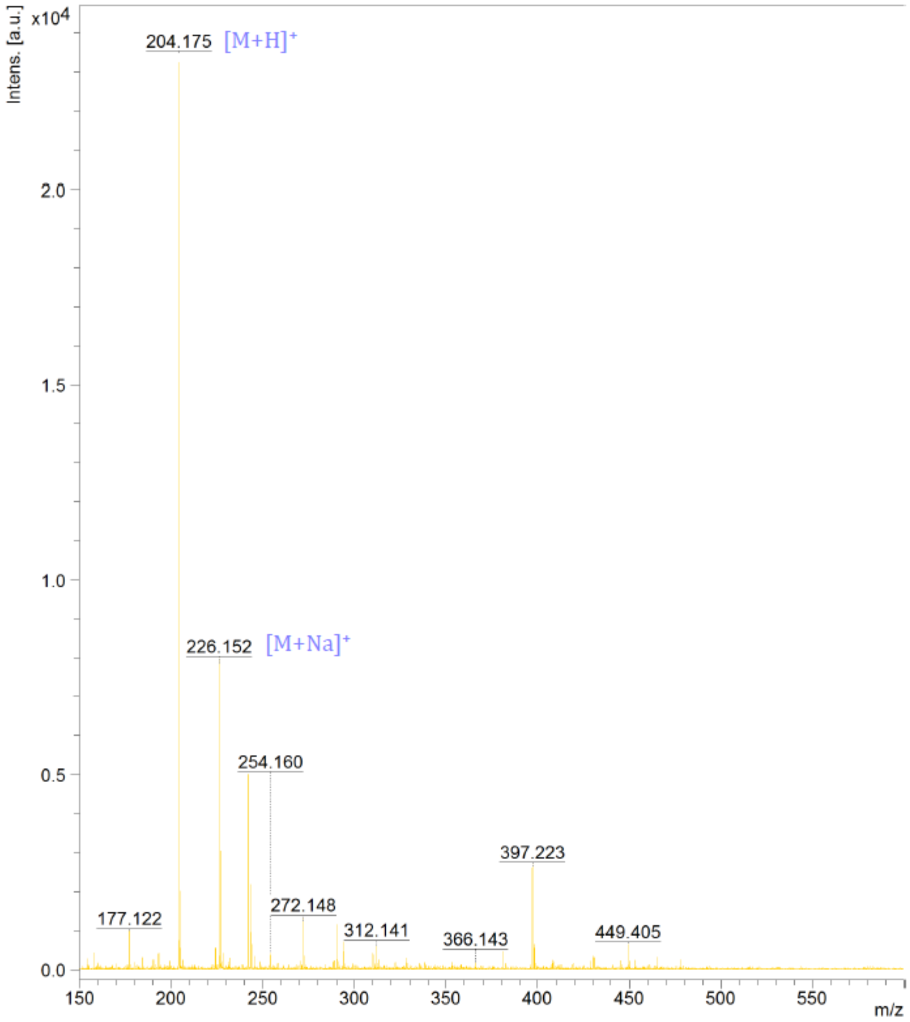

Figure S8. MALDI-MS spectrum of **1**.

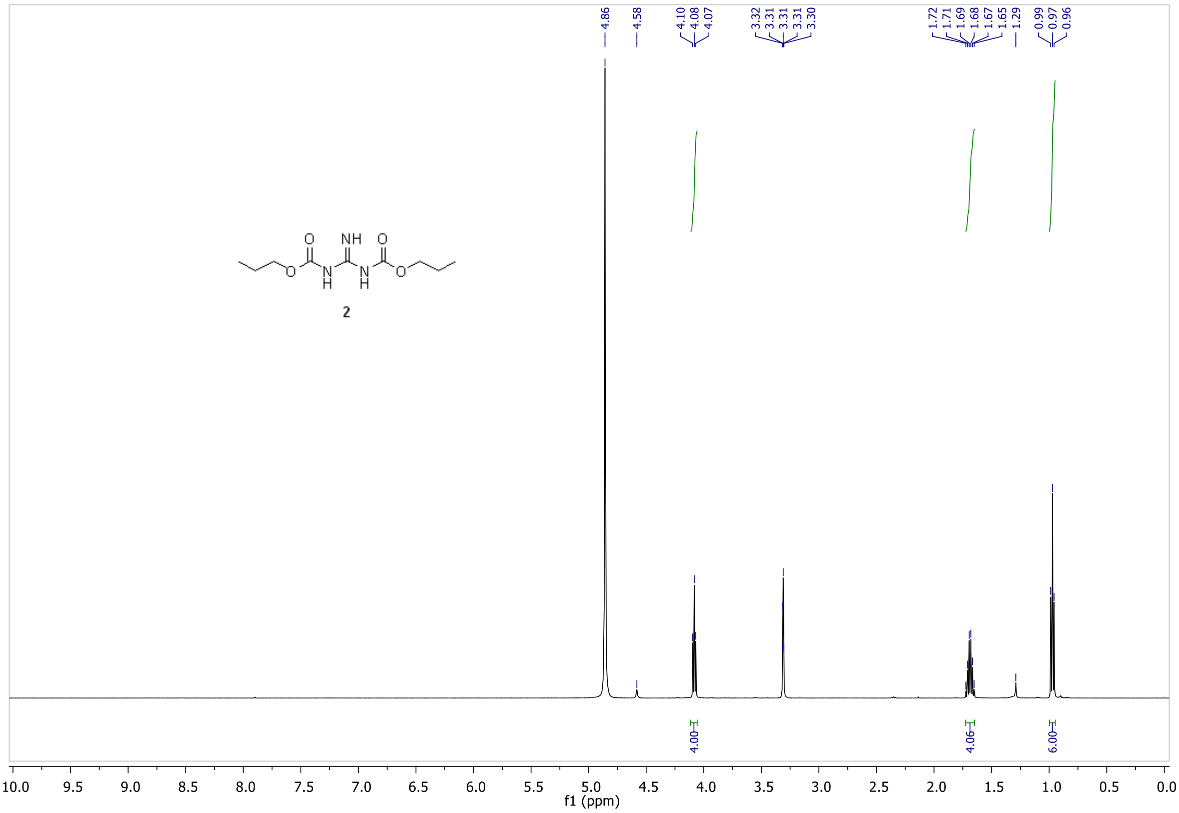

Figure S9. ^{1}H NMR spectrum of **2**.

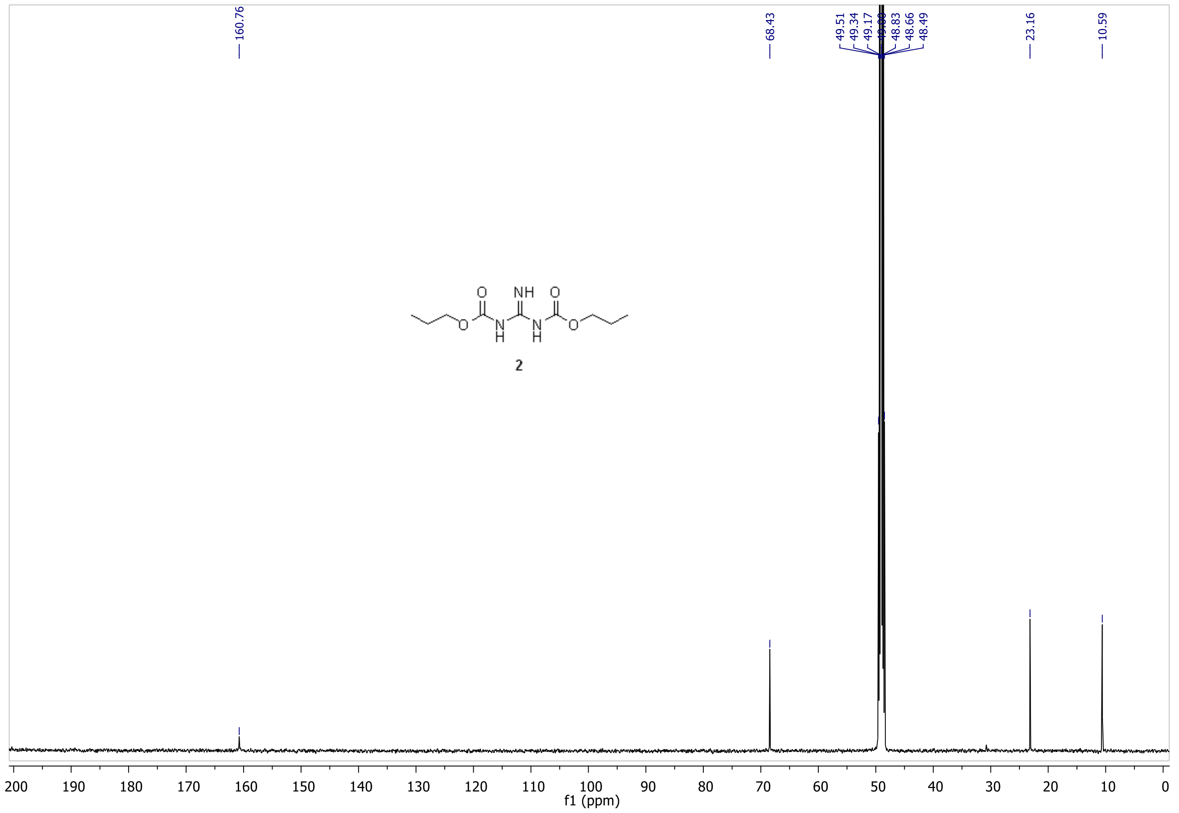

Figure S10. ^{13}C NMR spectrum of **2**.

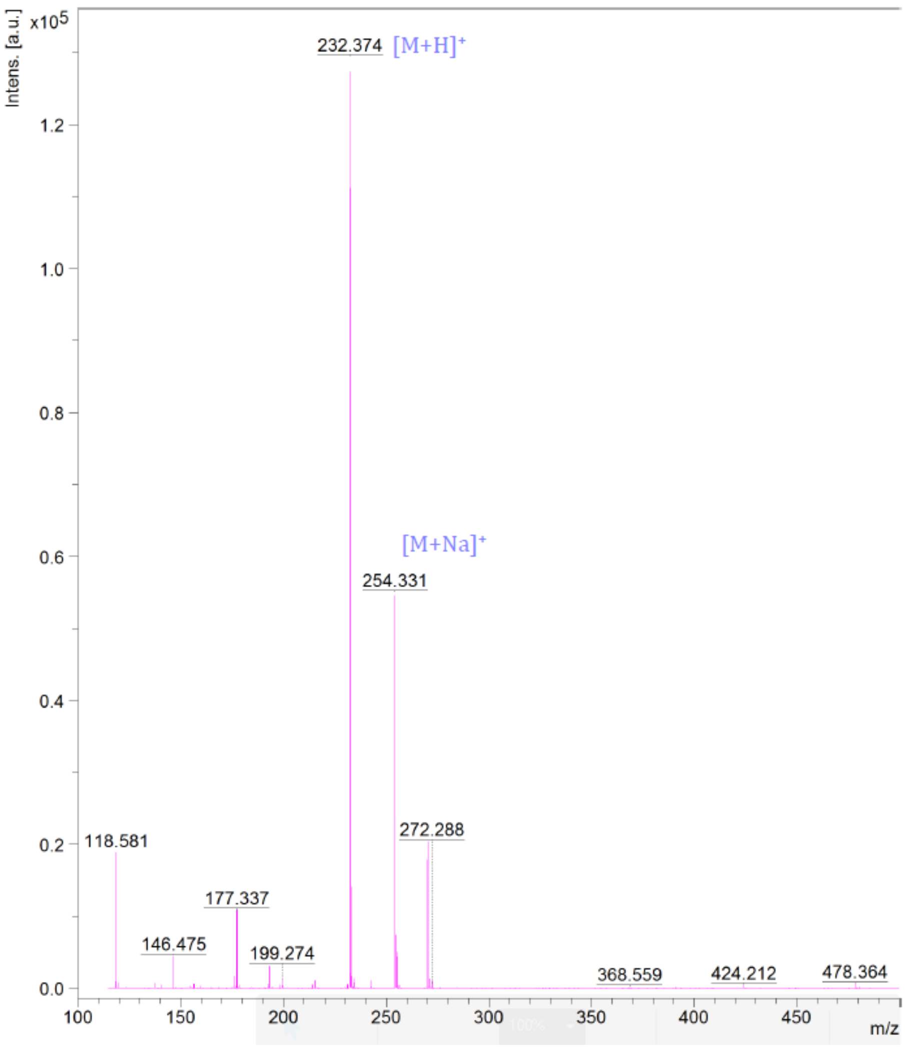

Figure S11. MALDI-MS spectrum of **2**.

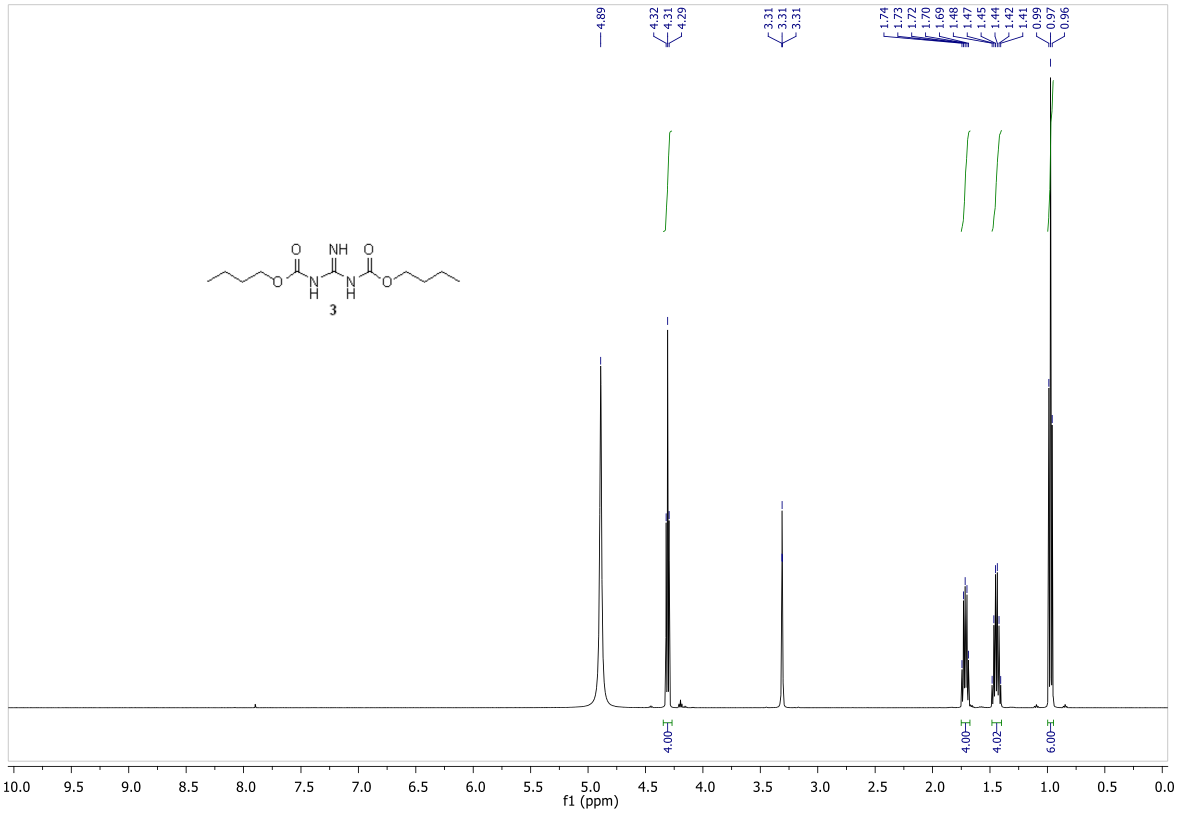

Figure S12. ^{1}H NMR spectrum of **3**.

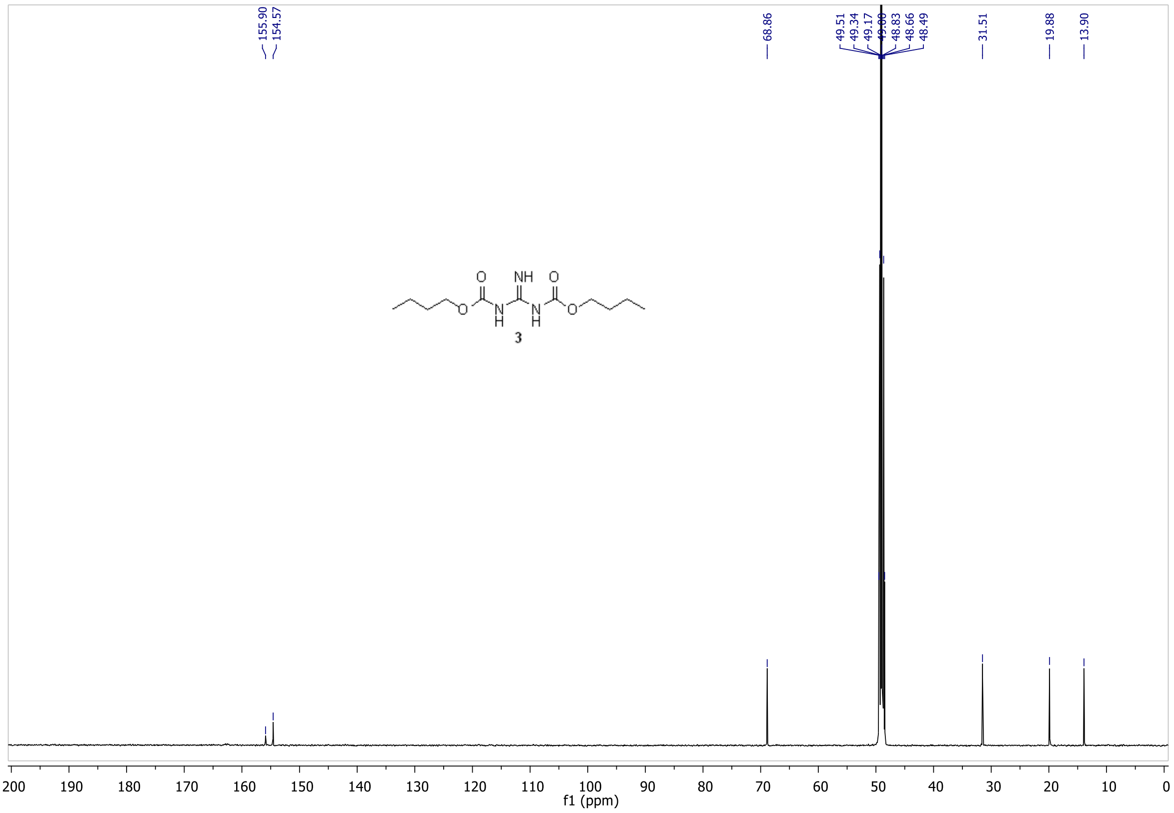

Figure S13. ^{13}C NMR spectrum of **3**.

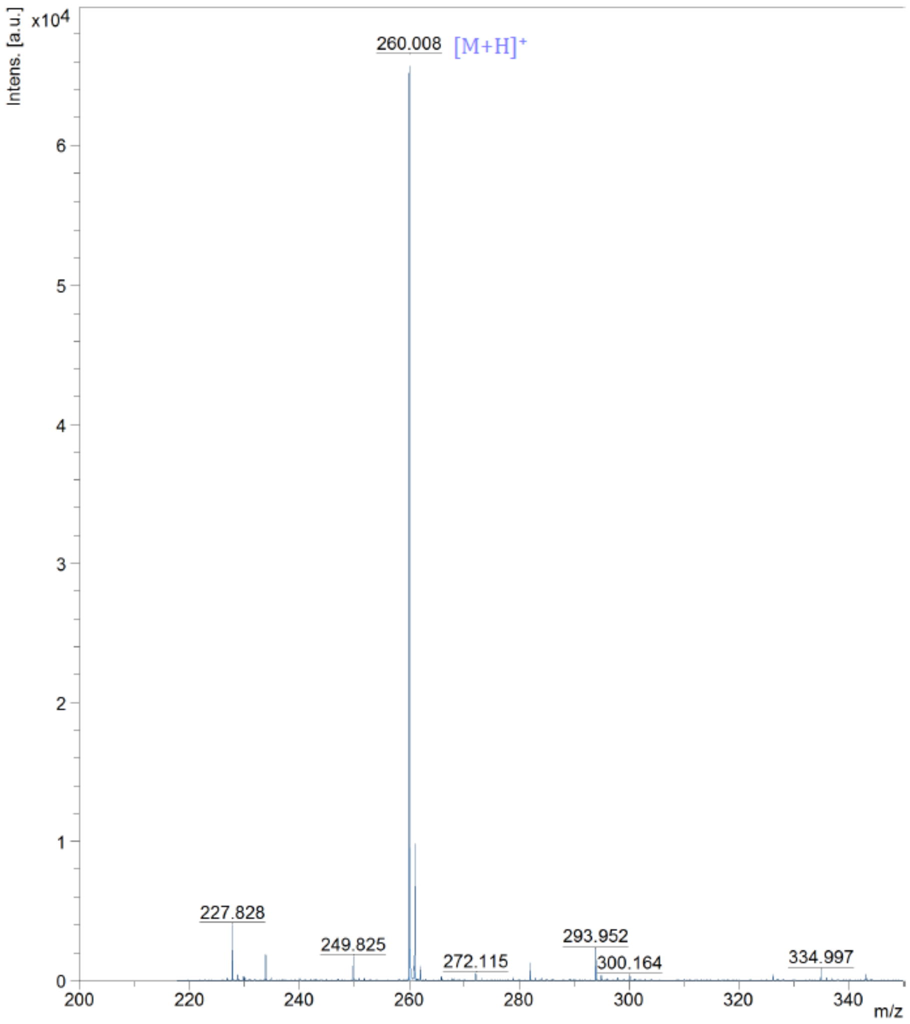

Figure S14. MALDI-MS spectrum of **3**.

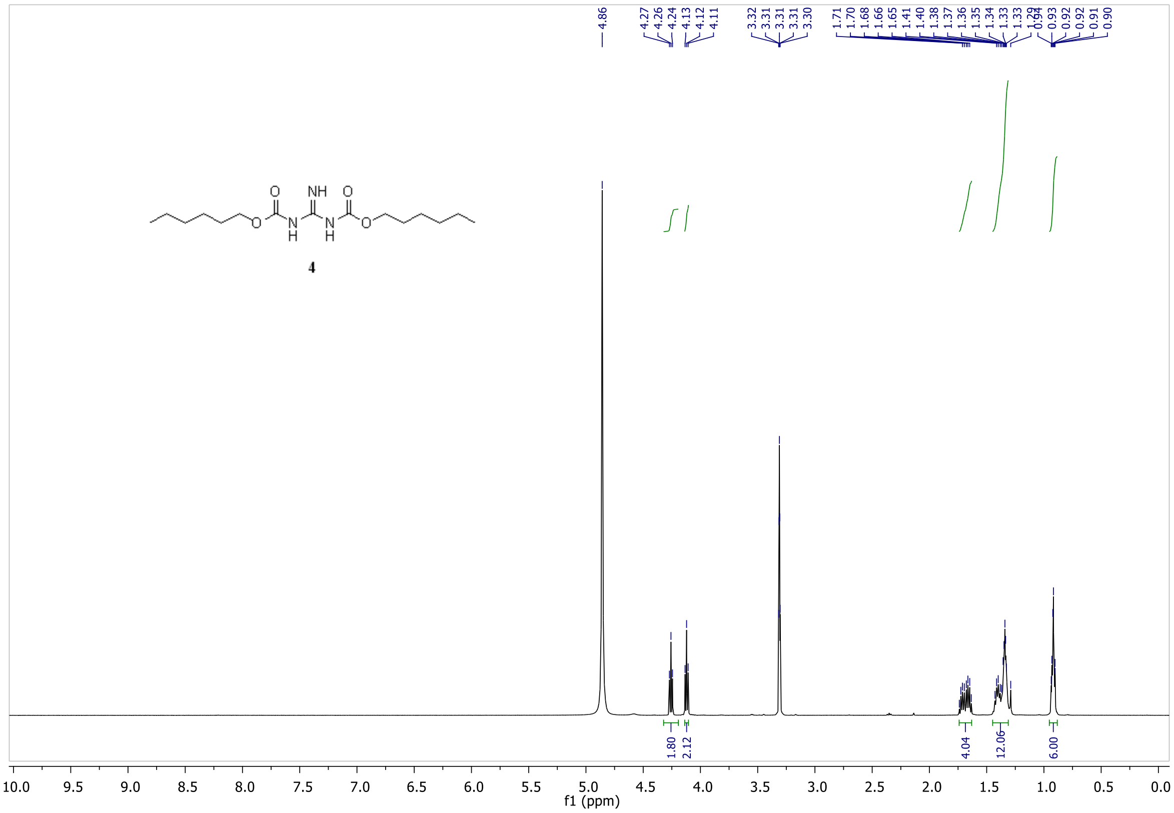

Figure S15. ^{1}H NMR spectrum of **4**.

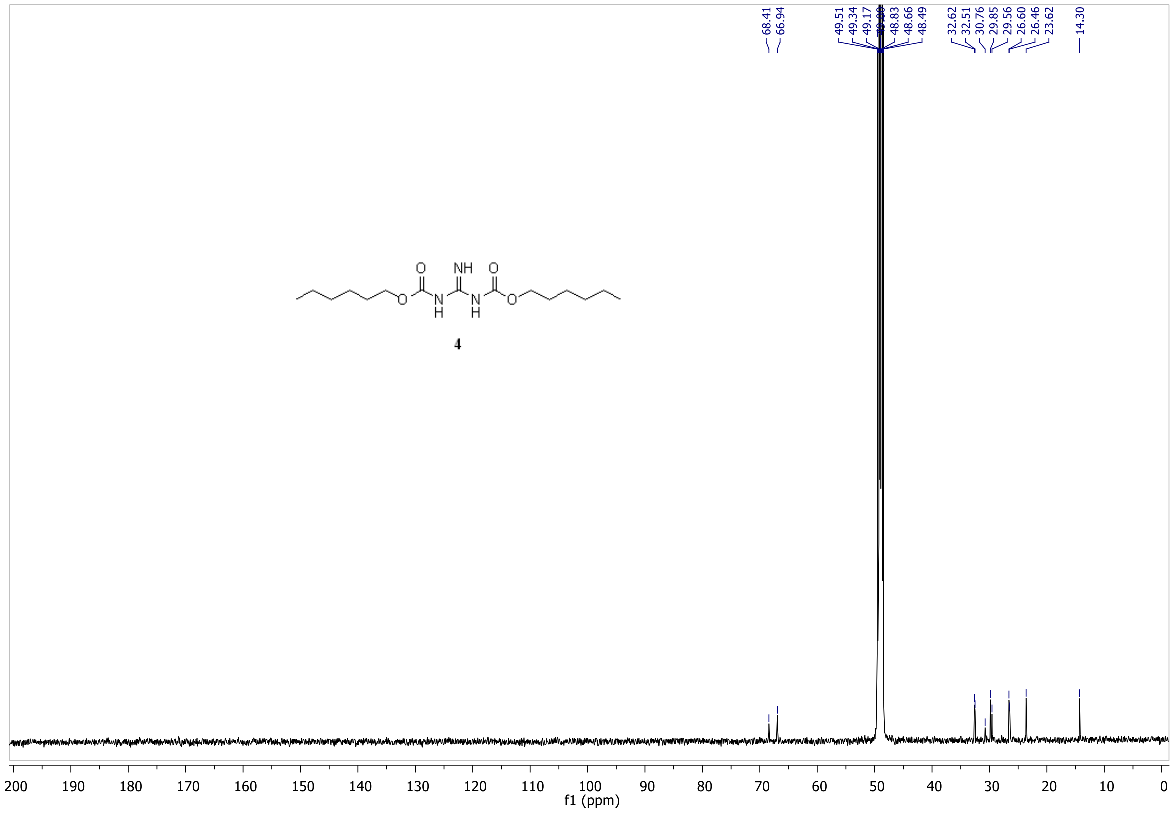

Figure S16. ^{13}C NMR spectrum of **4**.

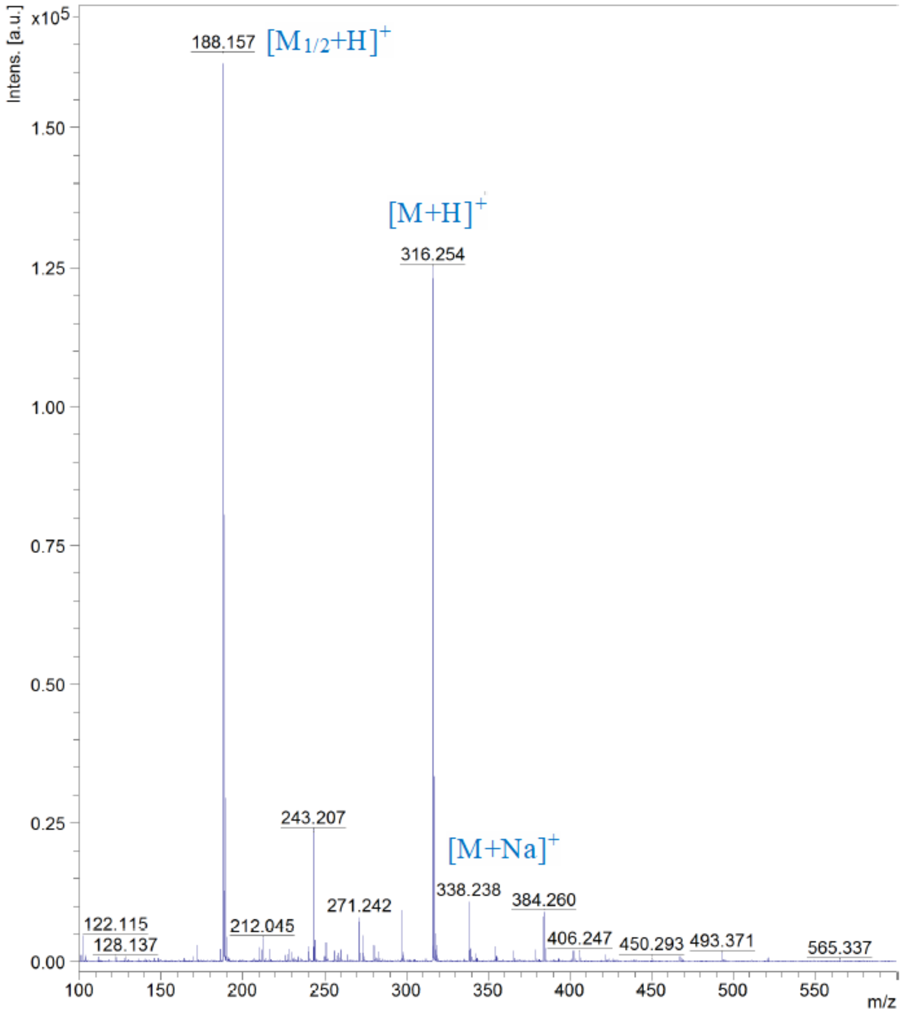

Figure S17. MALDI-MS spectrum of **4**.

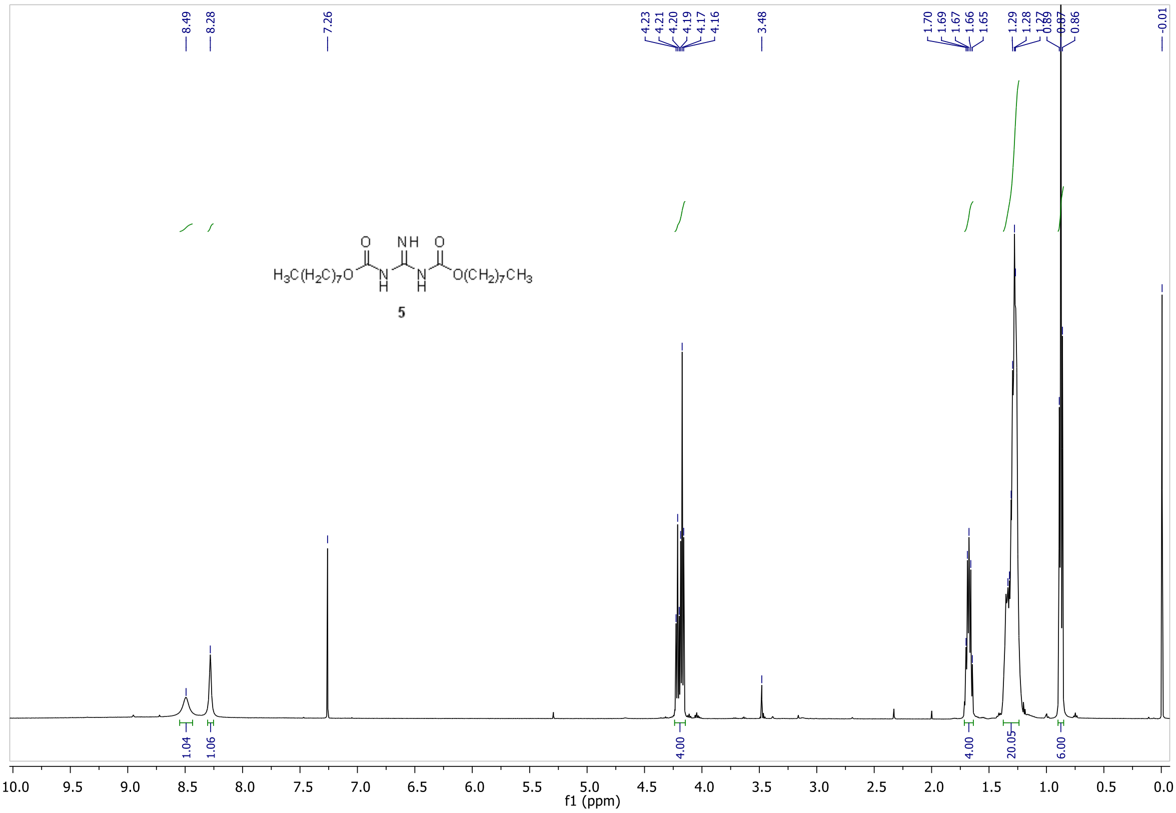

Figure S18. ^{1}H NMR spectrum of **5**.

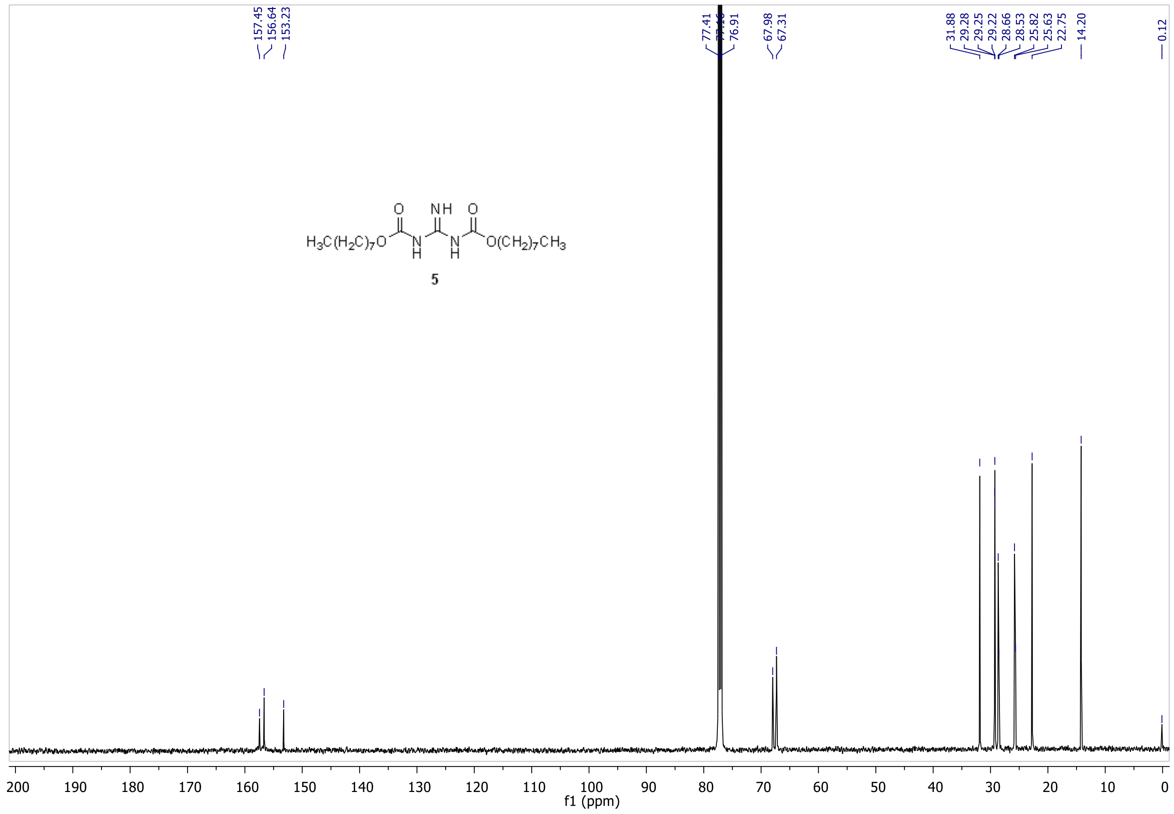

Figure S19. ^{13}C NMR spectrum of **5**.

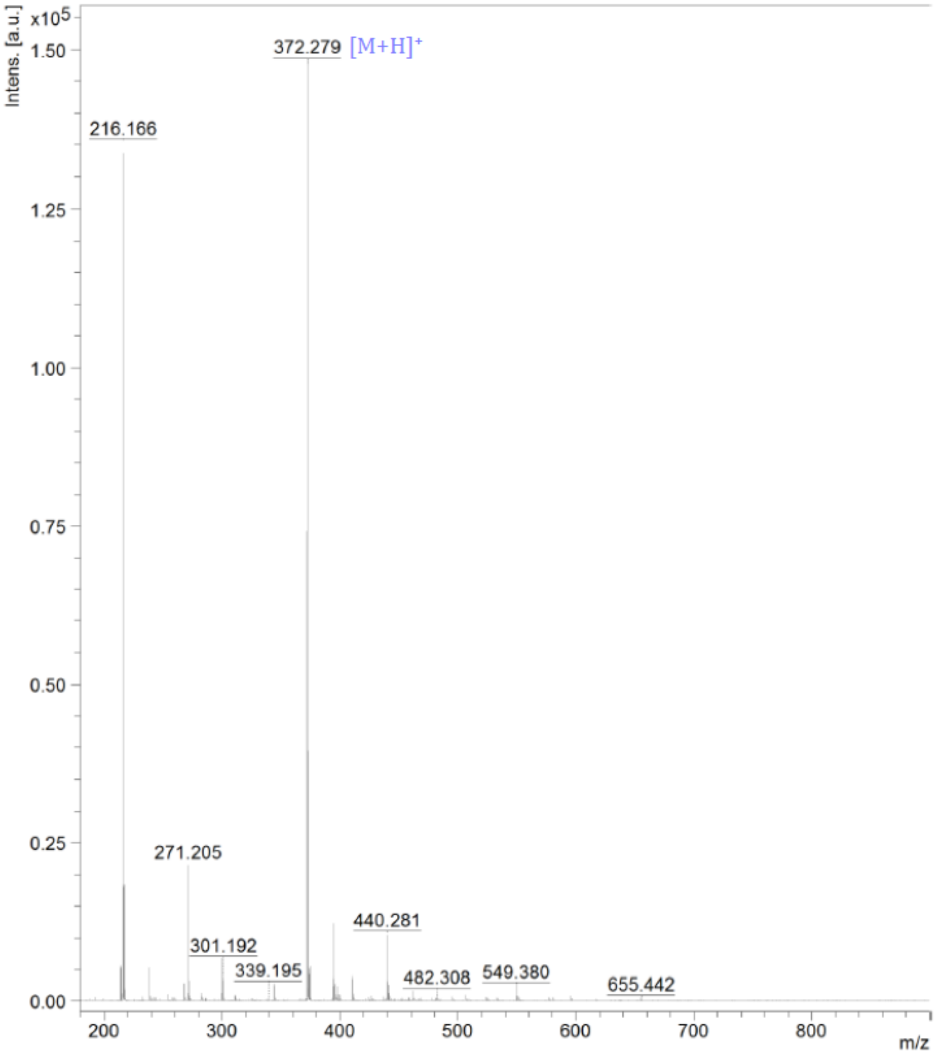

Figure S20. MALDI-MS spectrum of **5**.

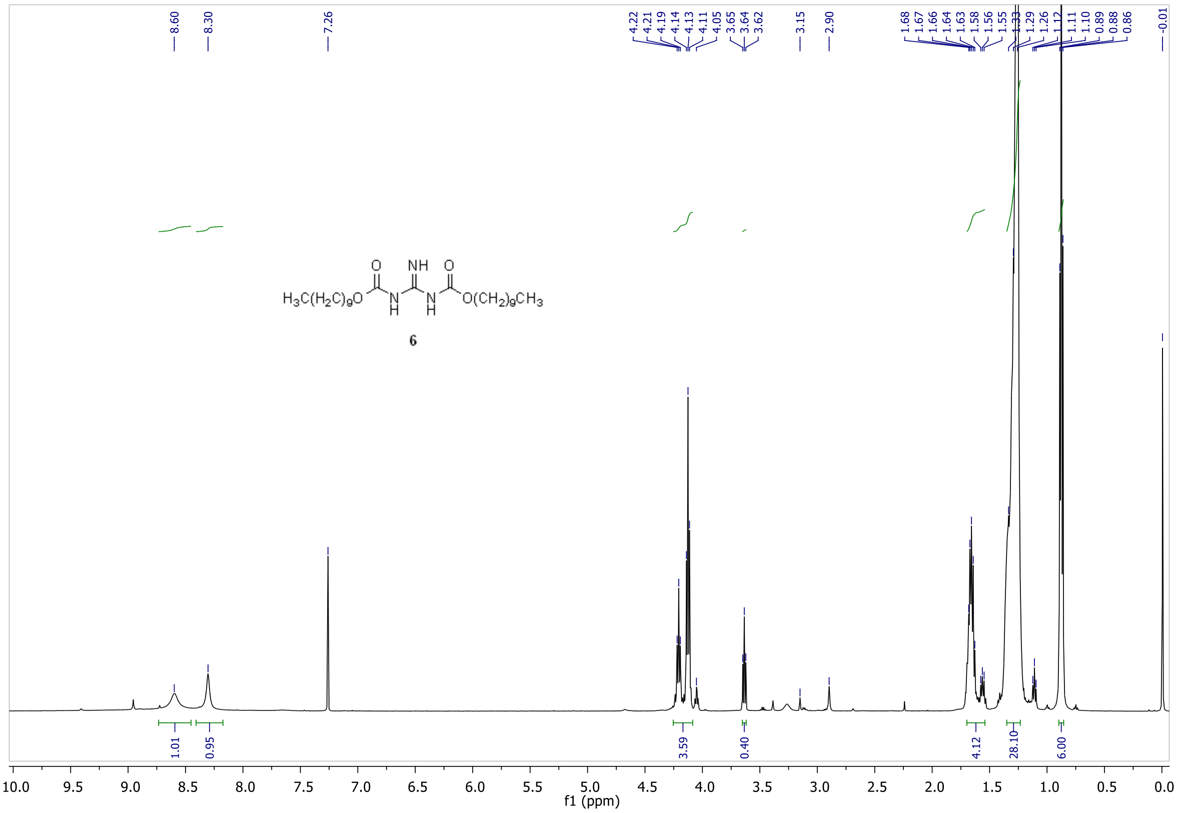

Figure S21. ^{1}H NMR spectrum of **6**.

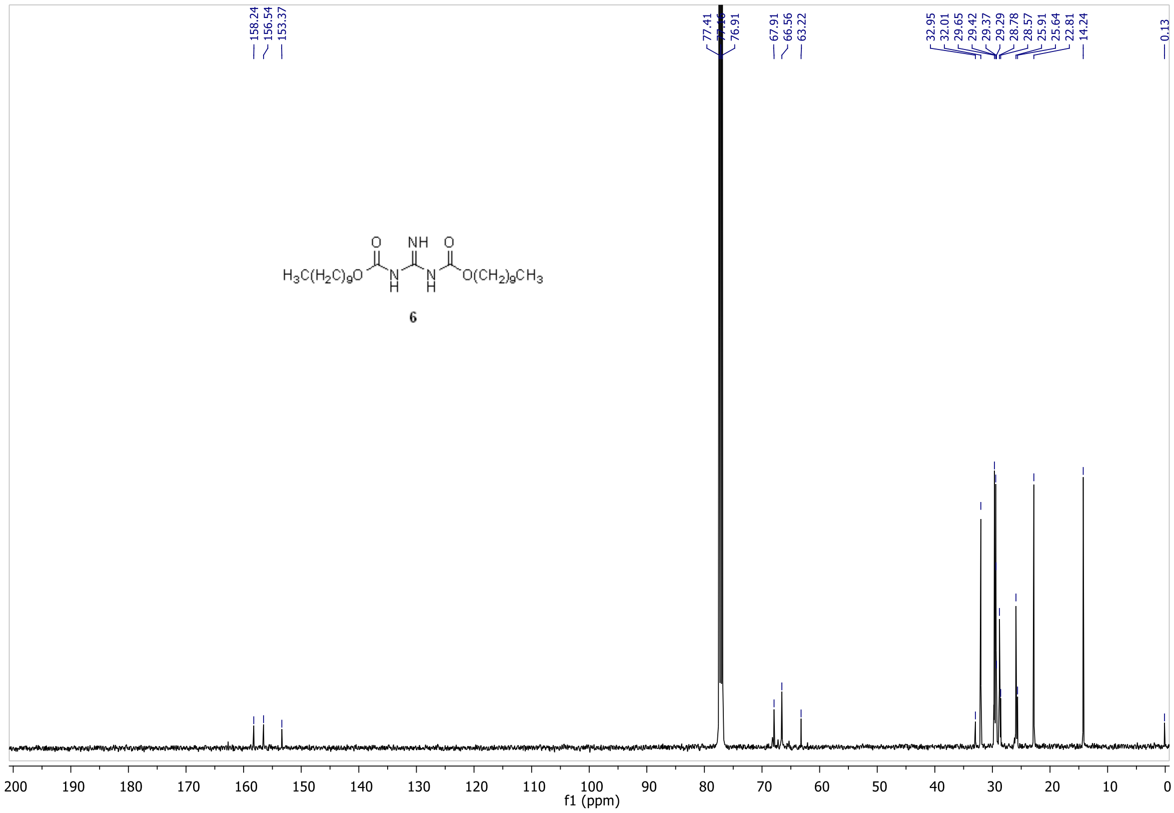

Figure S22. ^{13}C NMR spectrum of **6**.

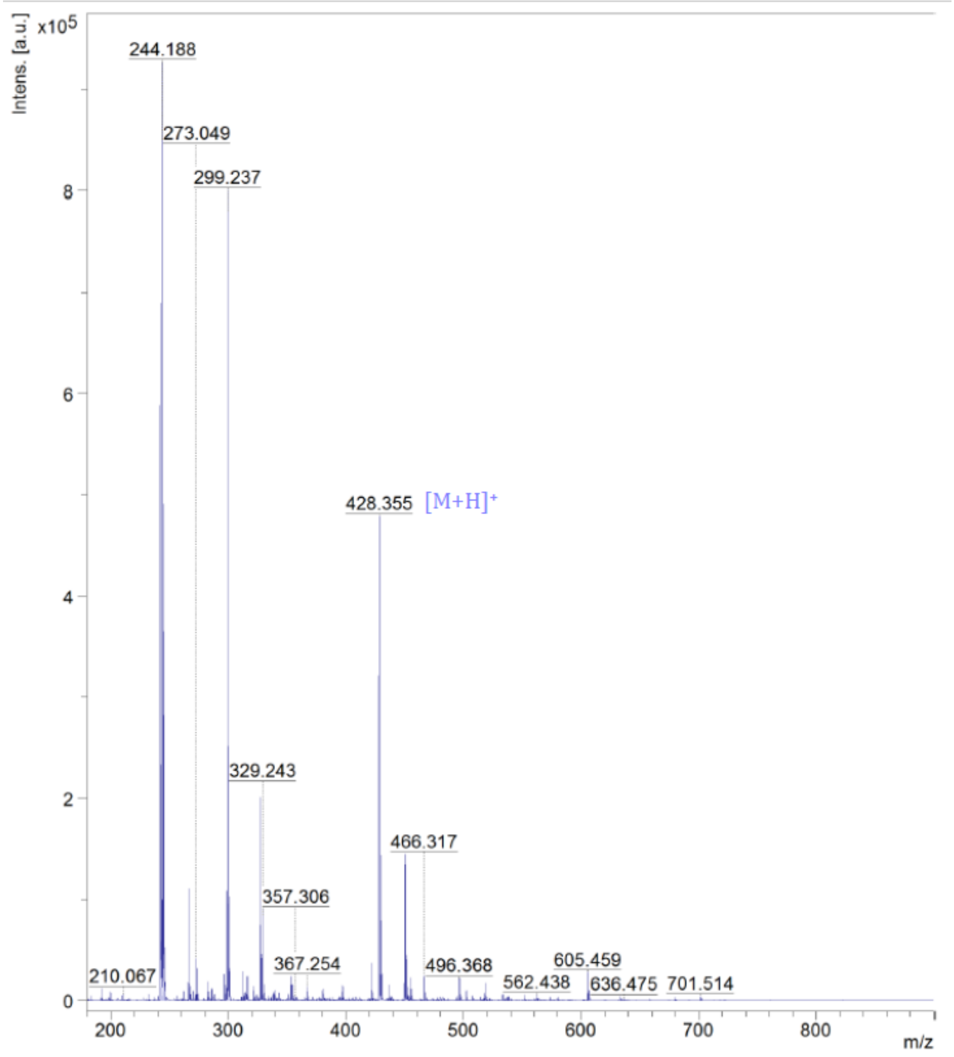

Figure S23. MALDI-MS spectrum of **6**.

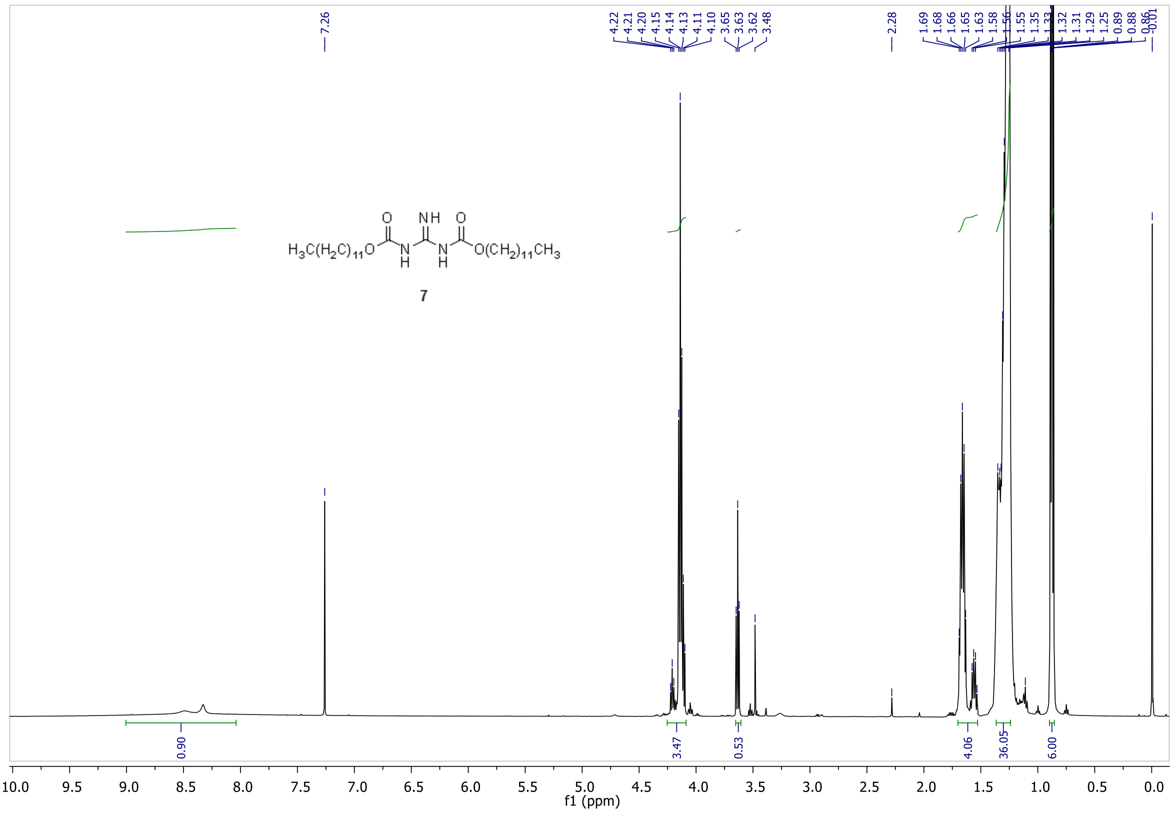

Figure S24. ^{1}H NMR spectrum of **7**.

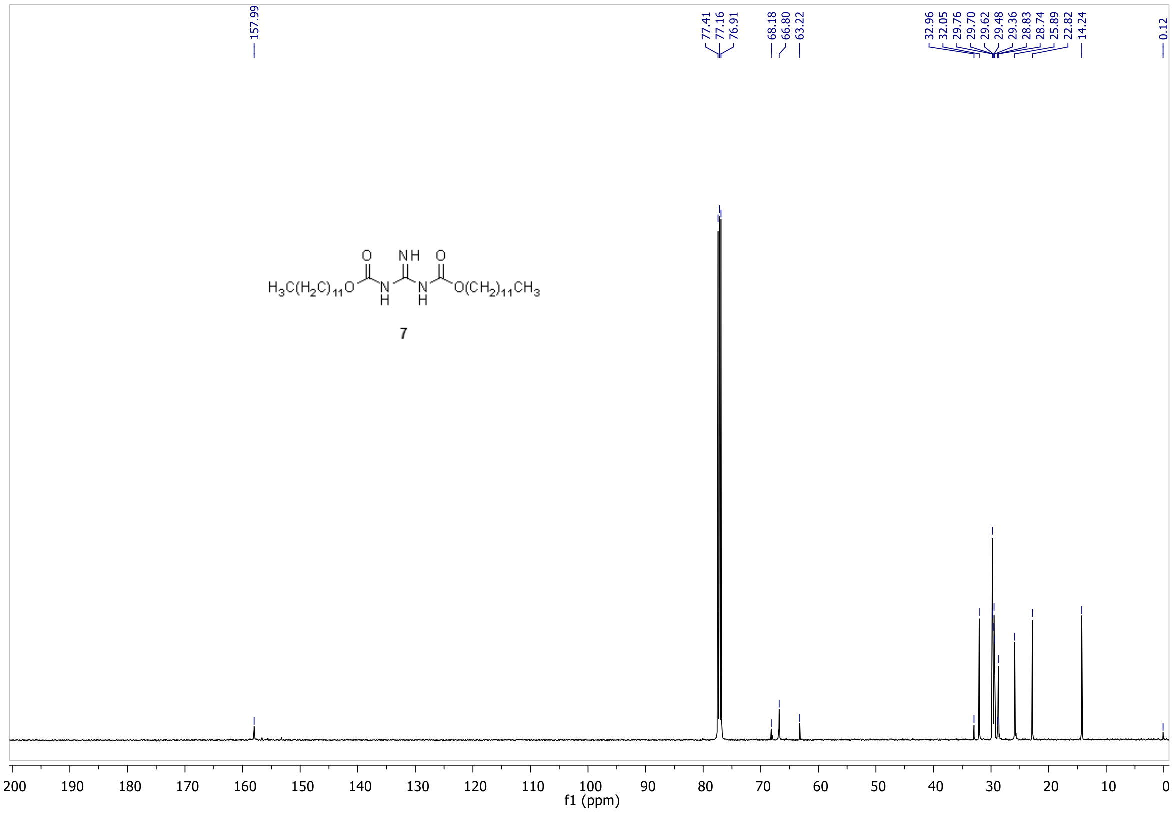

Figure S25. ^{13}C NMR spectrum of **7**.

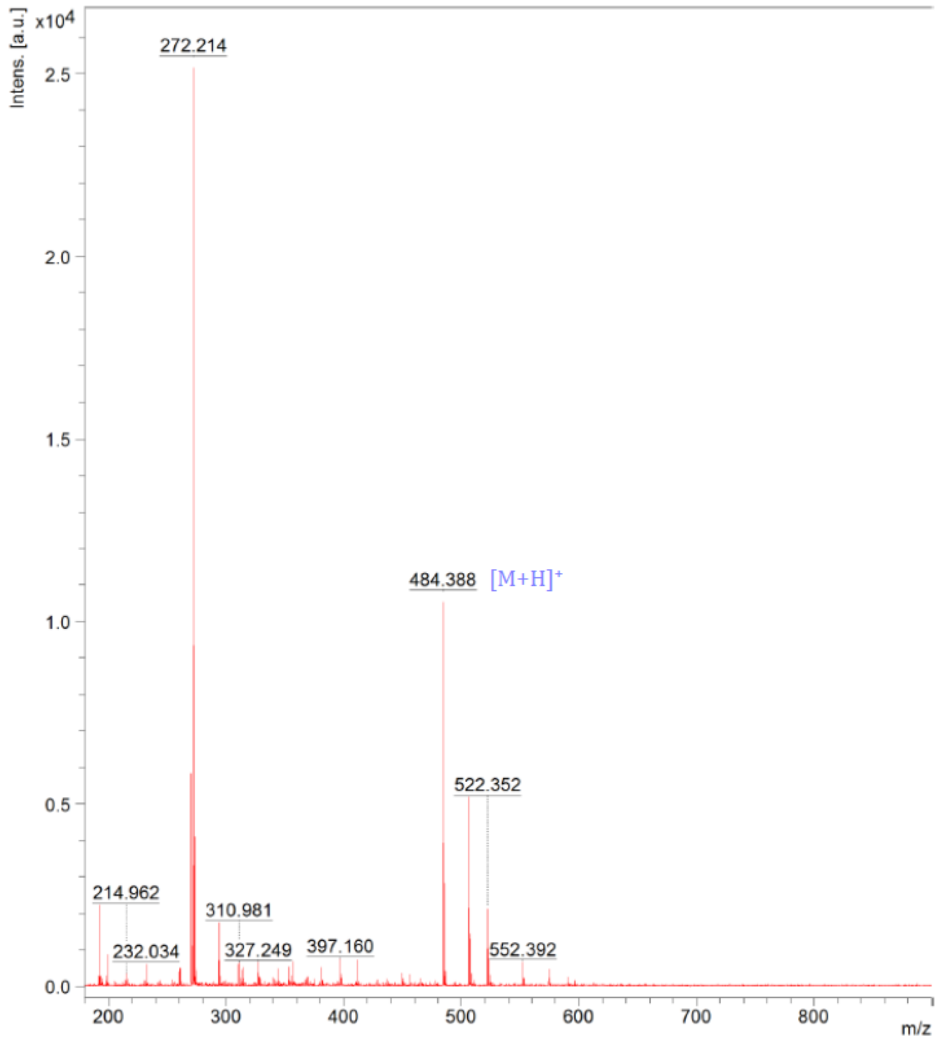

Figure S26. MALDI-MS spectrum of **7**.

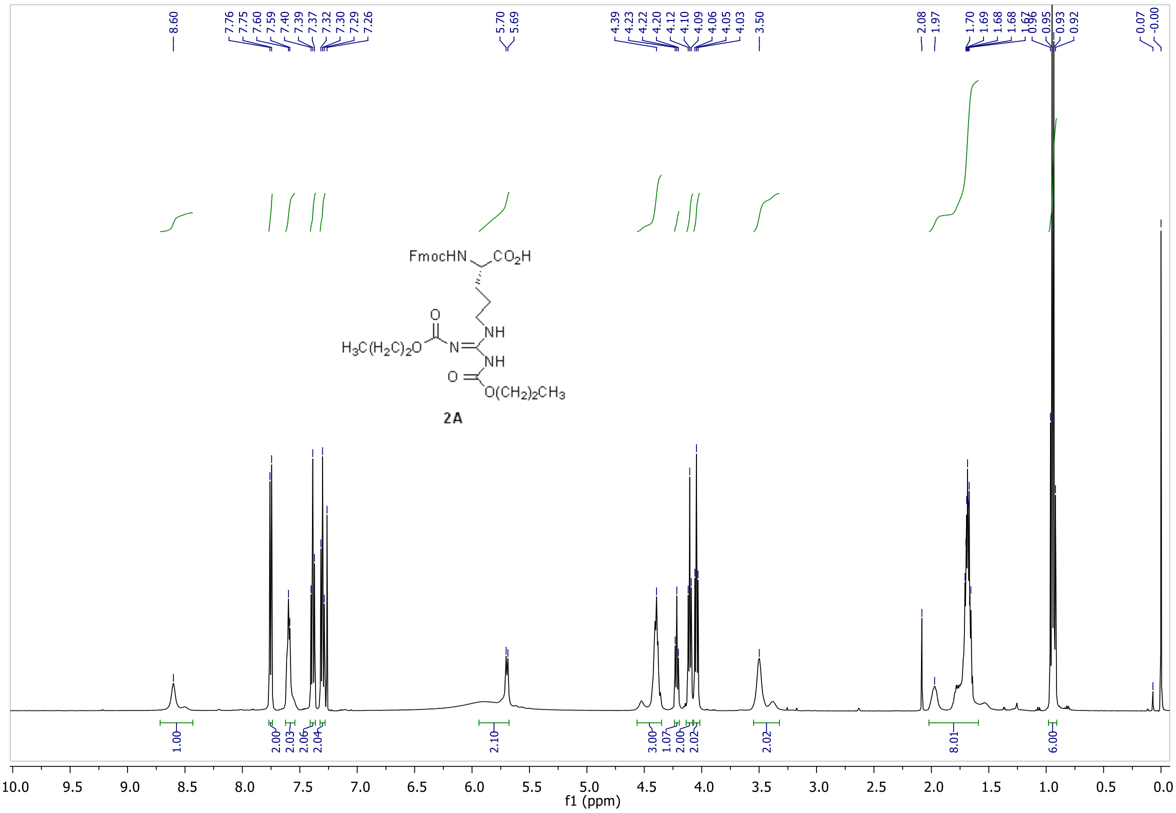

Figure S27. ^{1}H NMR spectrum of **2A**.

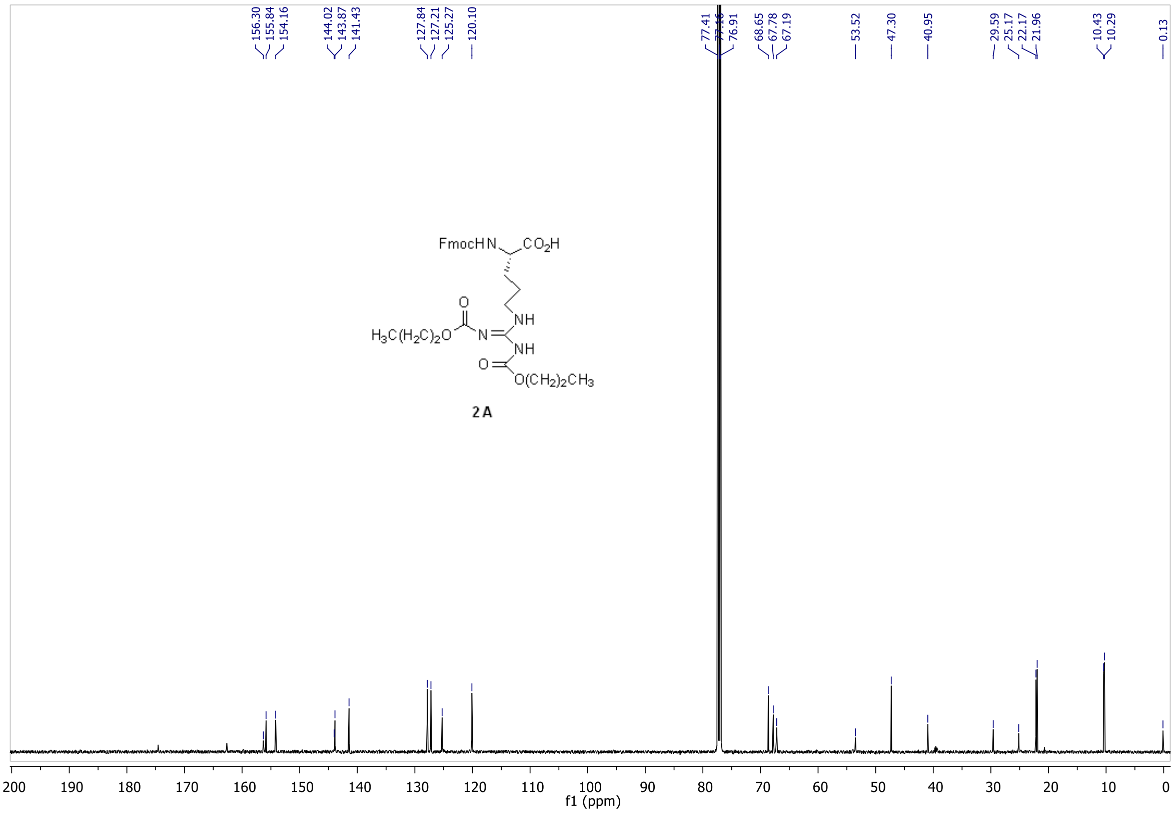

Figure S28. ^{13}C NMR spectrum of **2A**.

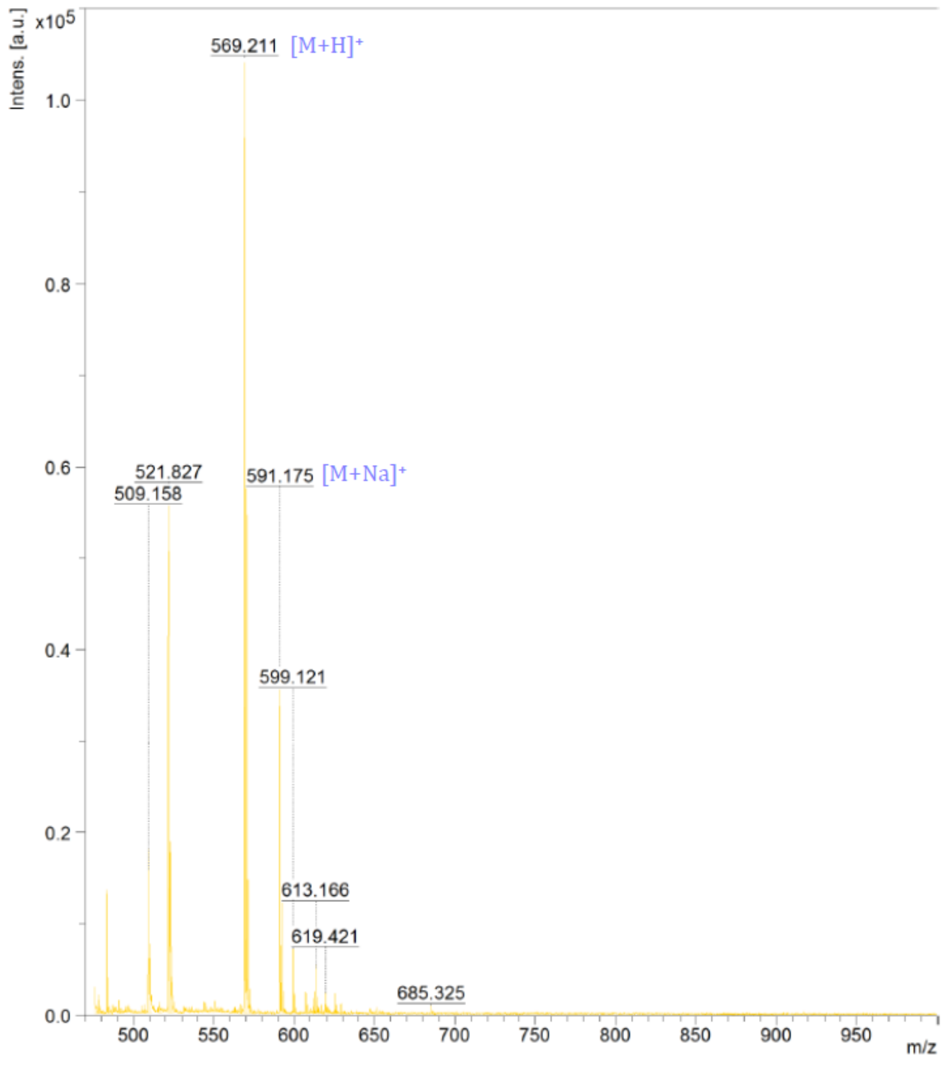

Figure S29. MALDI-MS spectrum of **2A**.

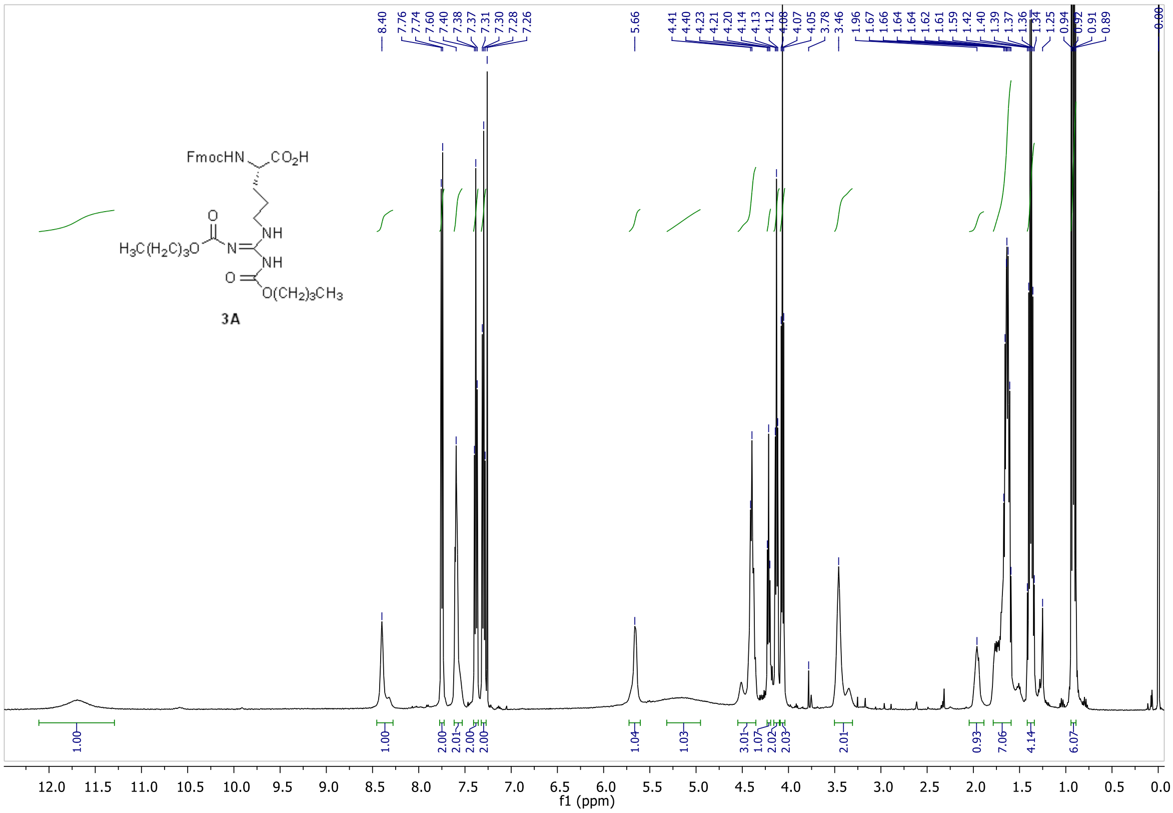

Figure S30. ^{1}H NMR spectrum of **3A**.

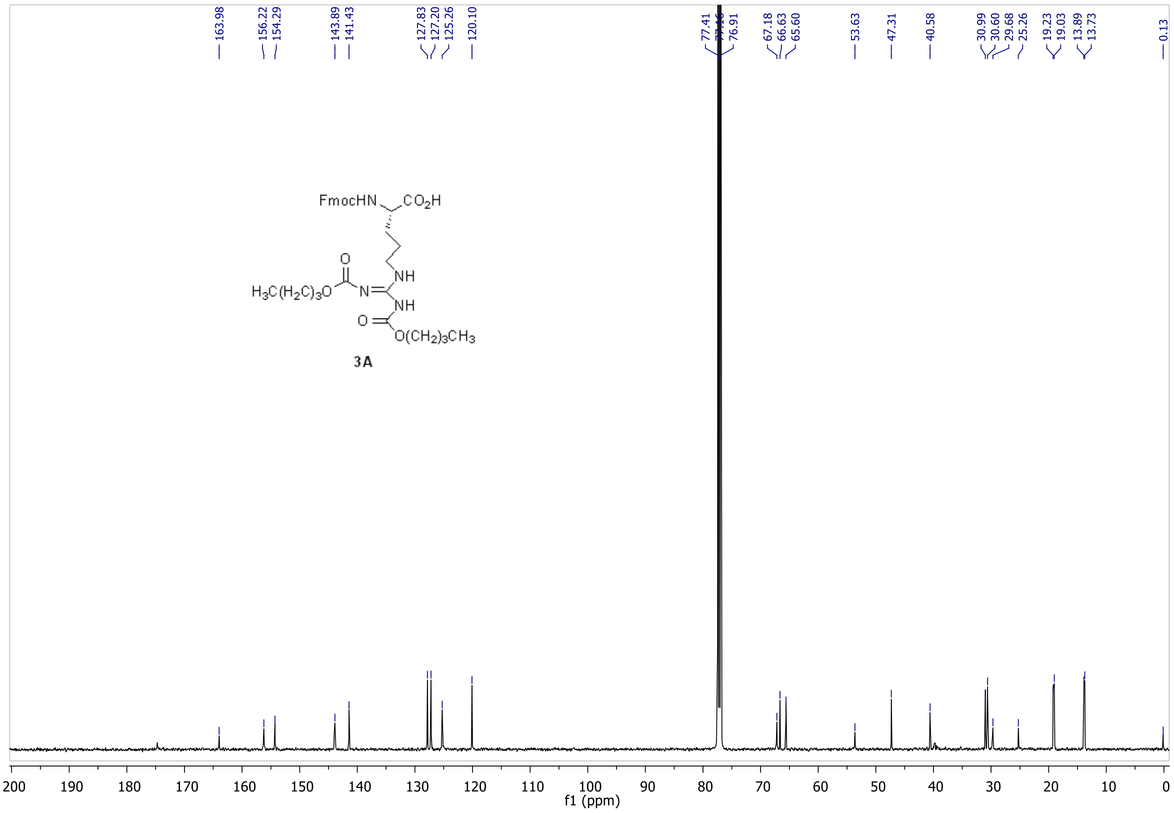

Figure S31. ^{13}C NMR spectrum of **3A**.

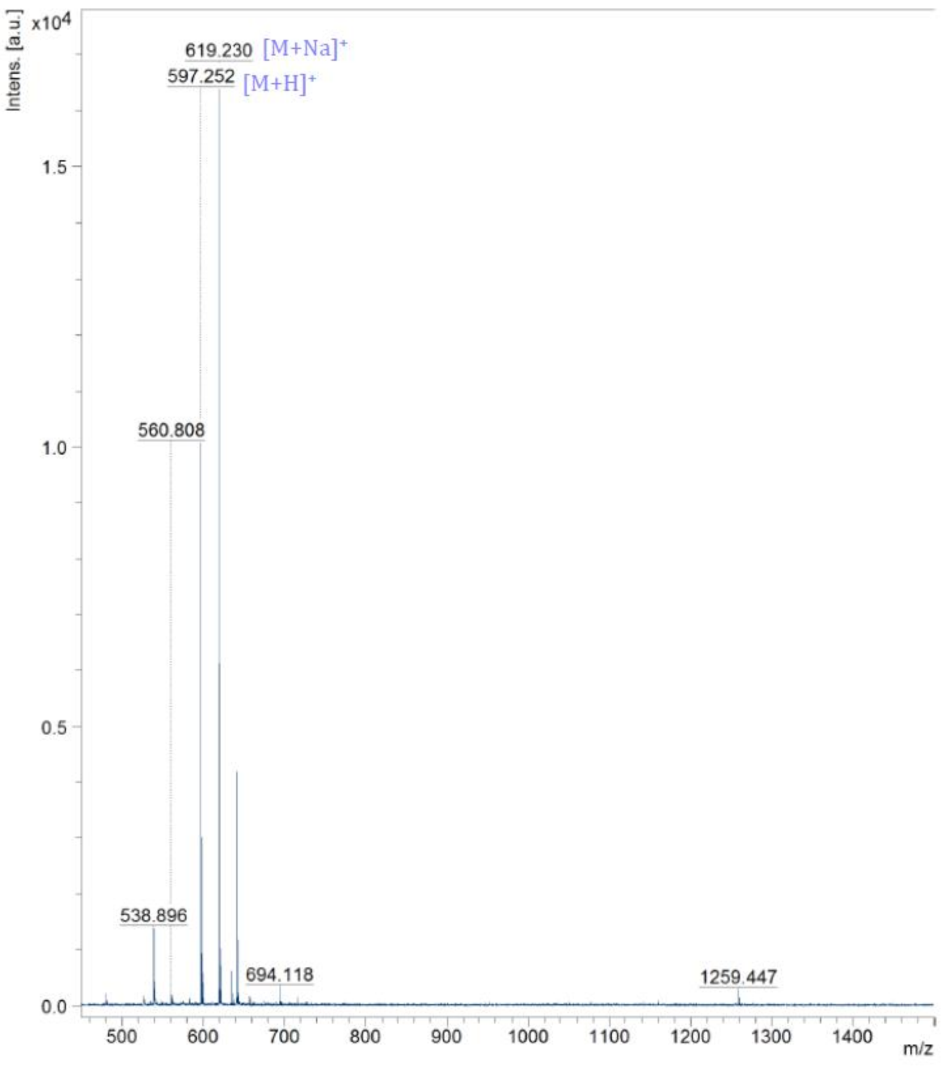

Figure S32. MALDI-MS spectrum of **3A**.

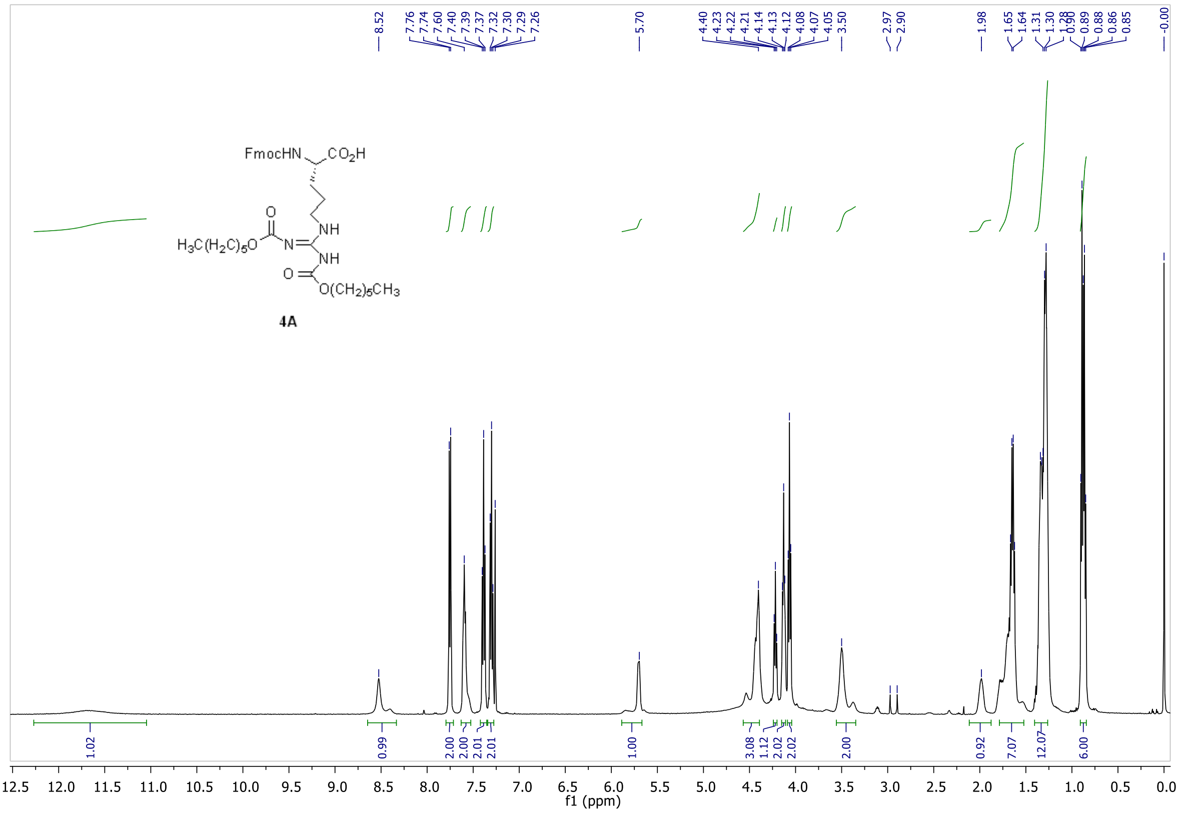

Figure S33. ^{1}H NMR spectrum of **4A**.

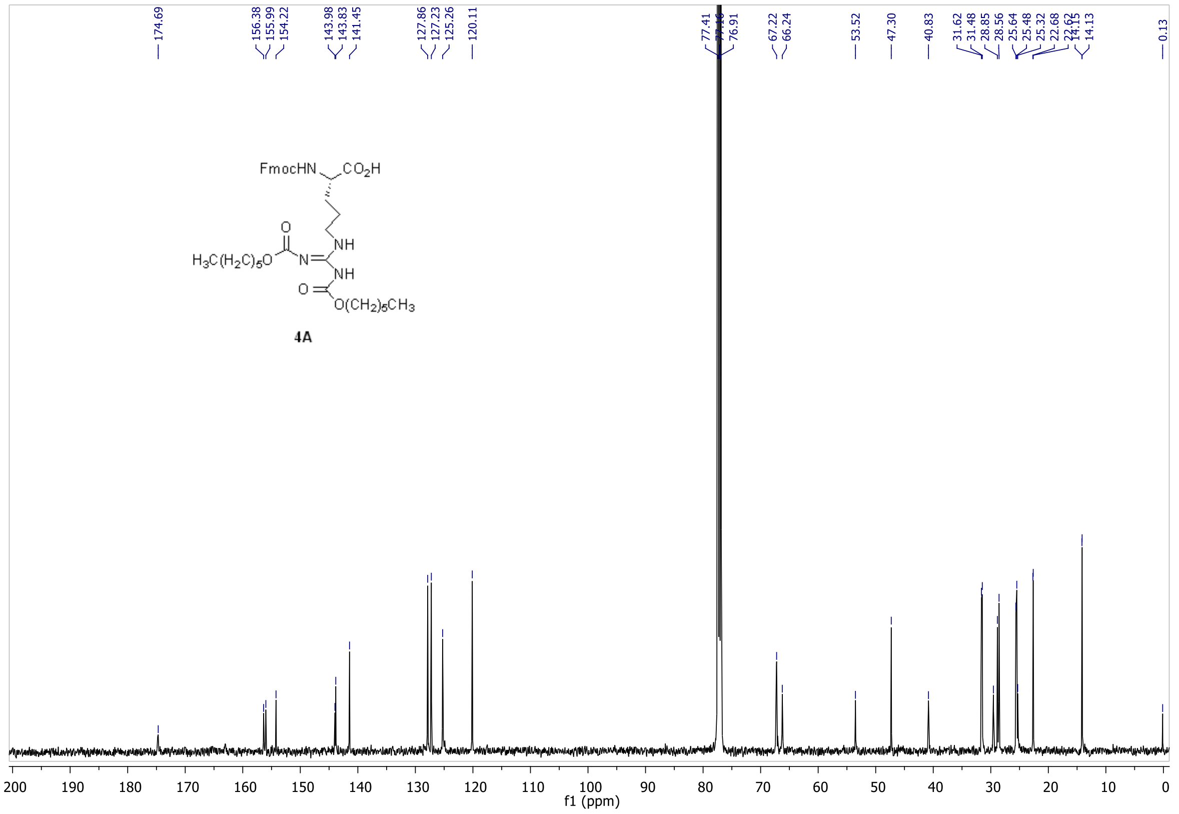

Figure S34. ^{13}C NMR spectrum of **4A**.

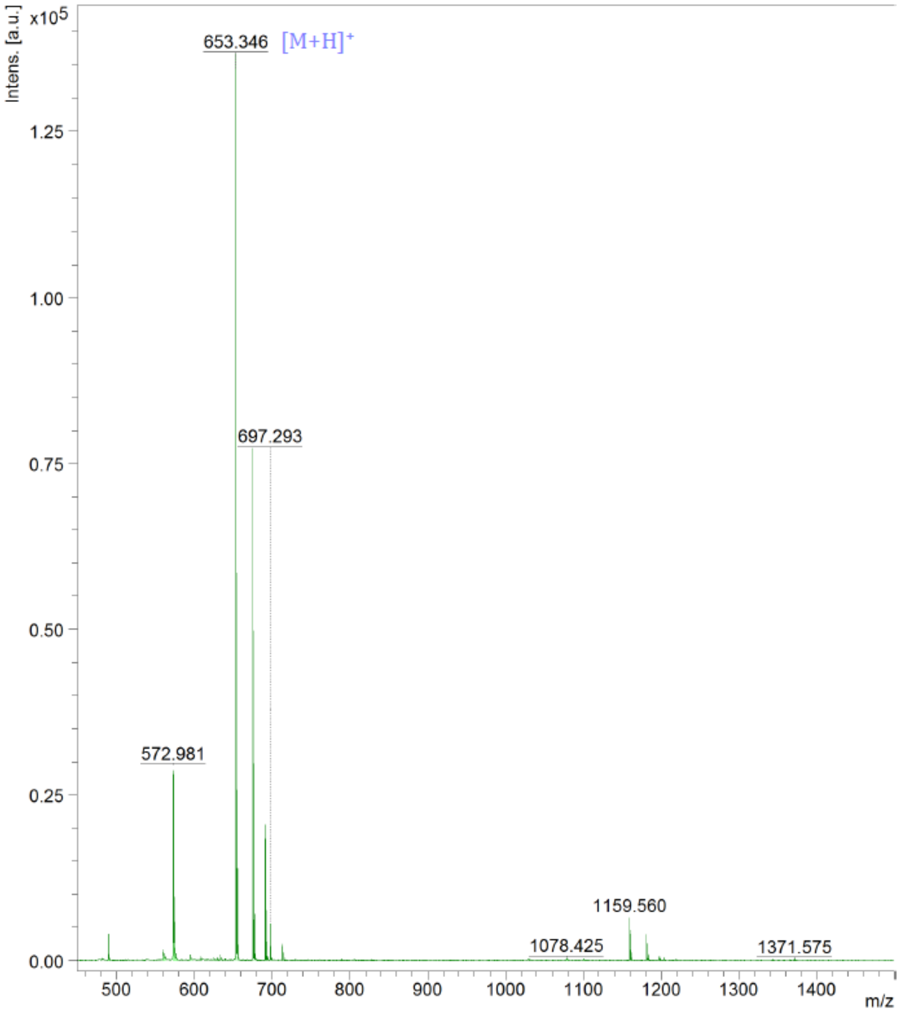

Figure S35. MALDI-MS spectrum of **4A.**

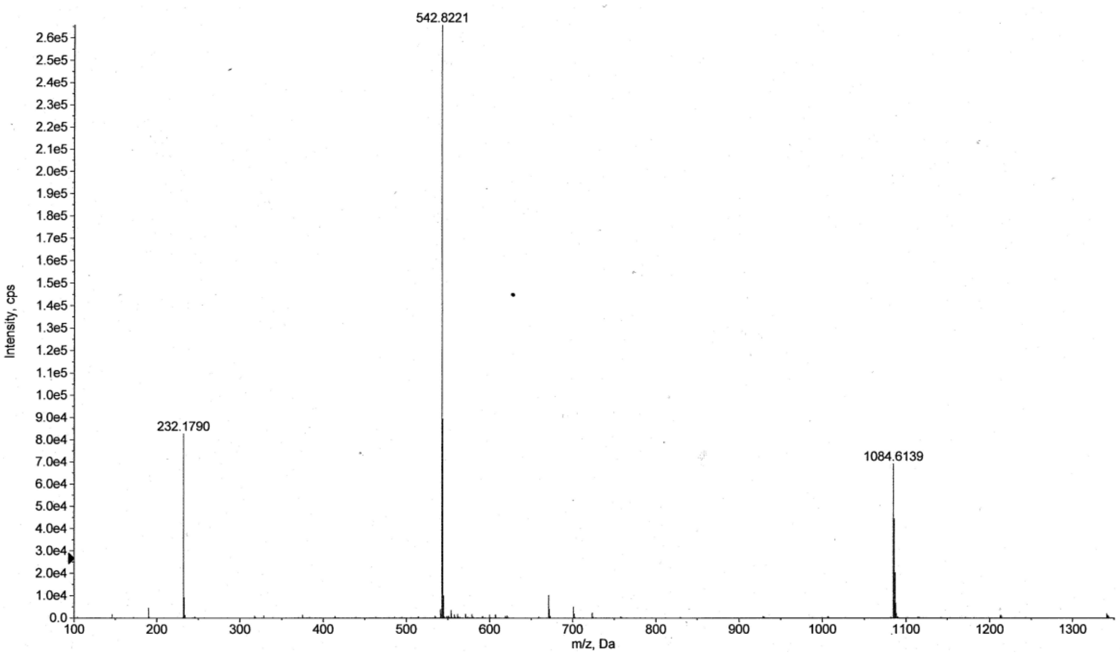

Figure S36. ESI-MS spectrum of **AVP** (232.1790 corresponds to H_2N-Arg-Gly-NH_2 fragmentation part).

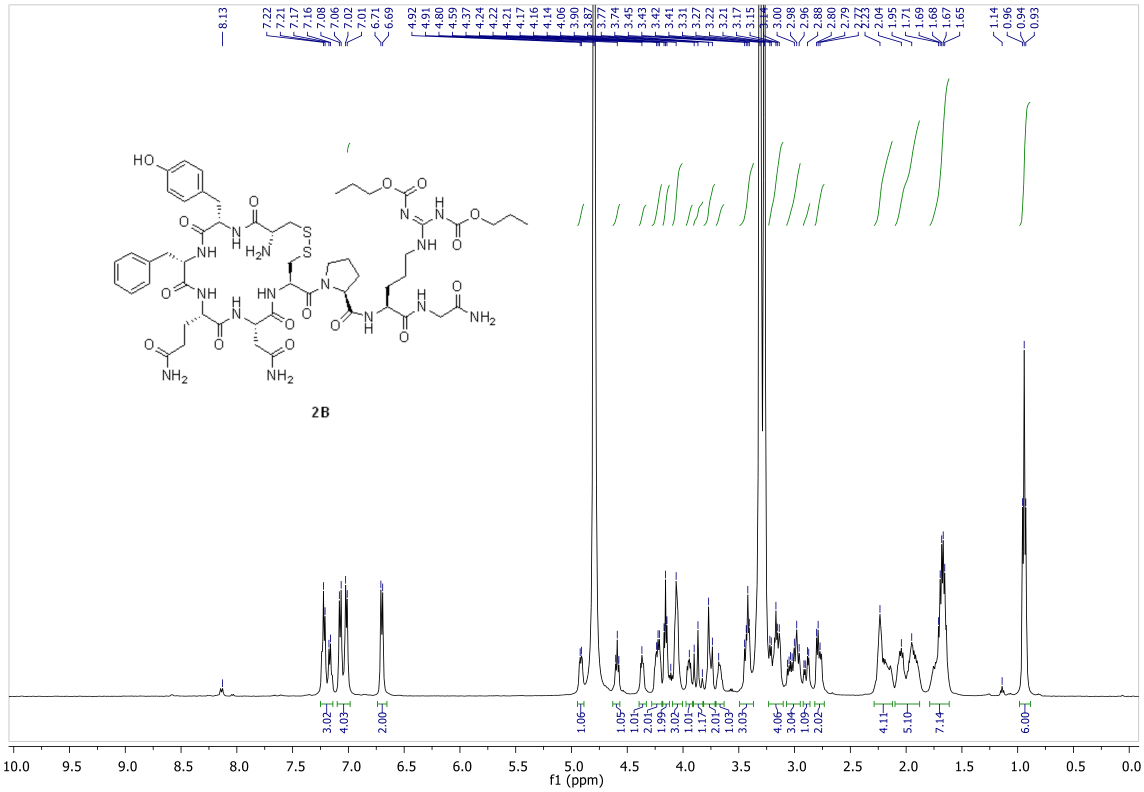

Figure S37. ^{1}H NMR spectrum of **2B**.

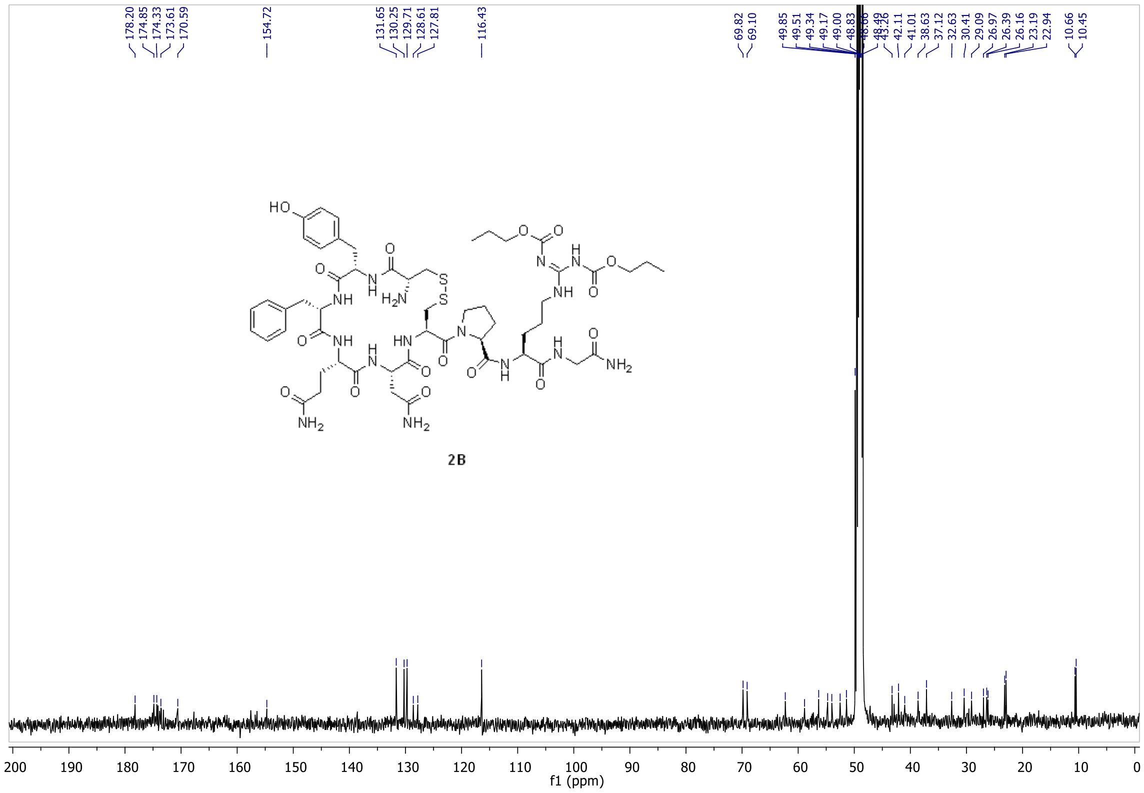

Figure S38. ^{13}C NMR spectrum of **2B**.

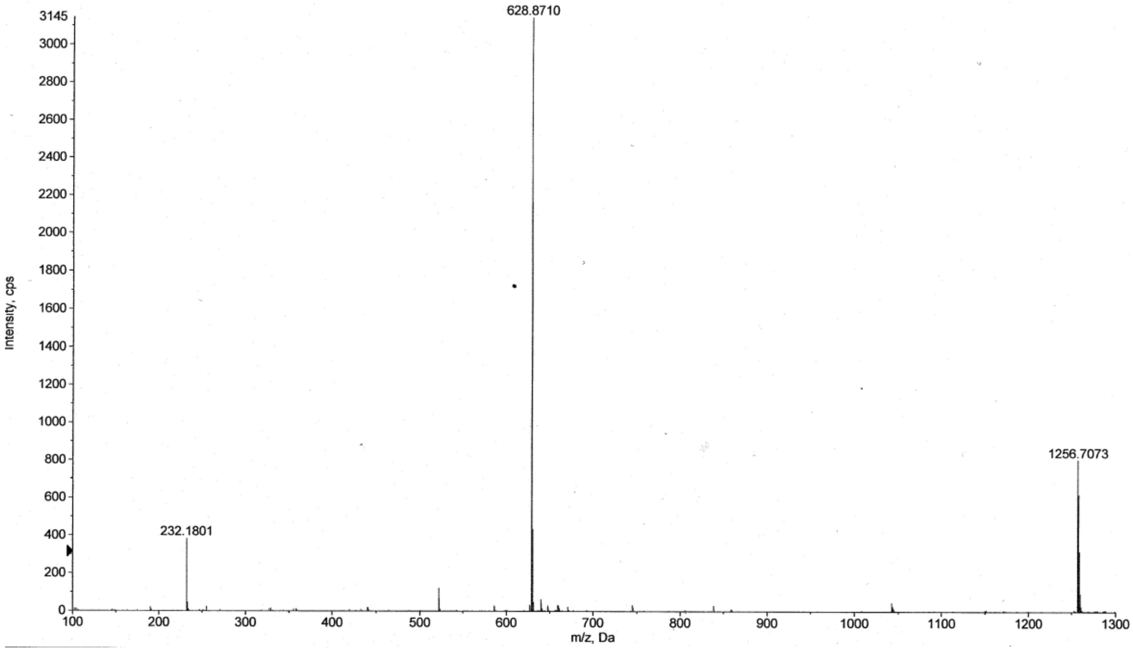

Figure S39. ESI-MS spectrum of **2B**.

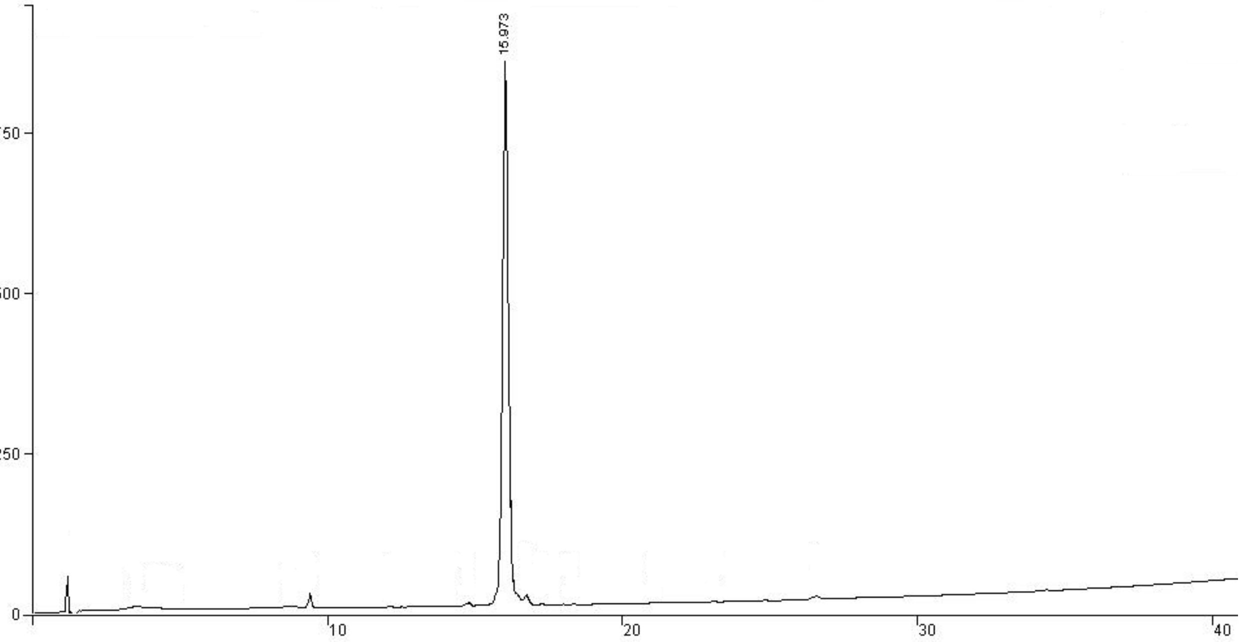

Figure S40. HPLC spectrum of **2B**.

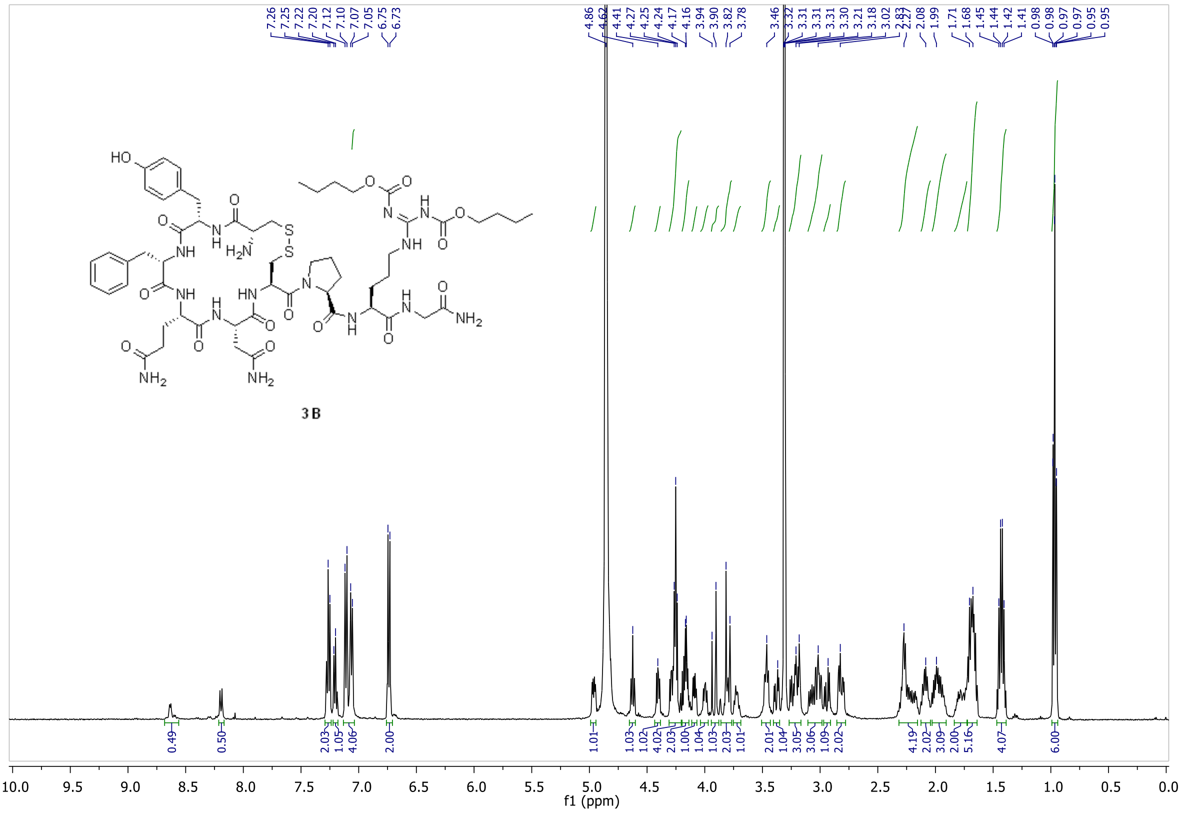

Figure S41. ^{1}H NMR spectrum of **3B**.

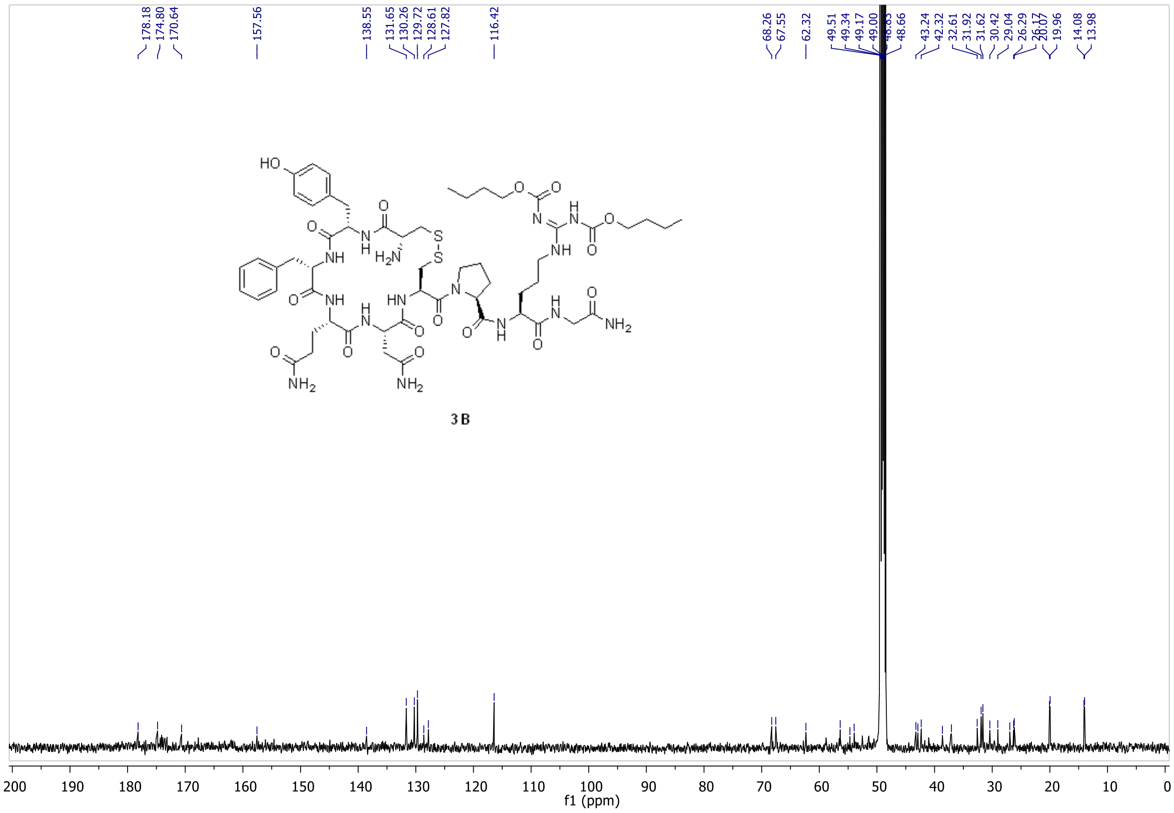

Figure S42. ^{13}C NMR spectrum of **3B**.

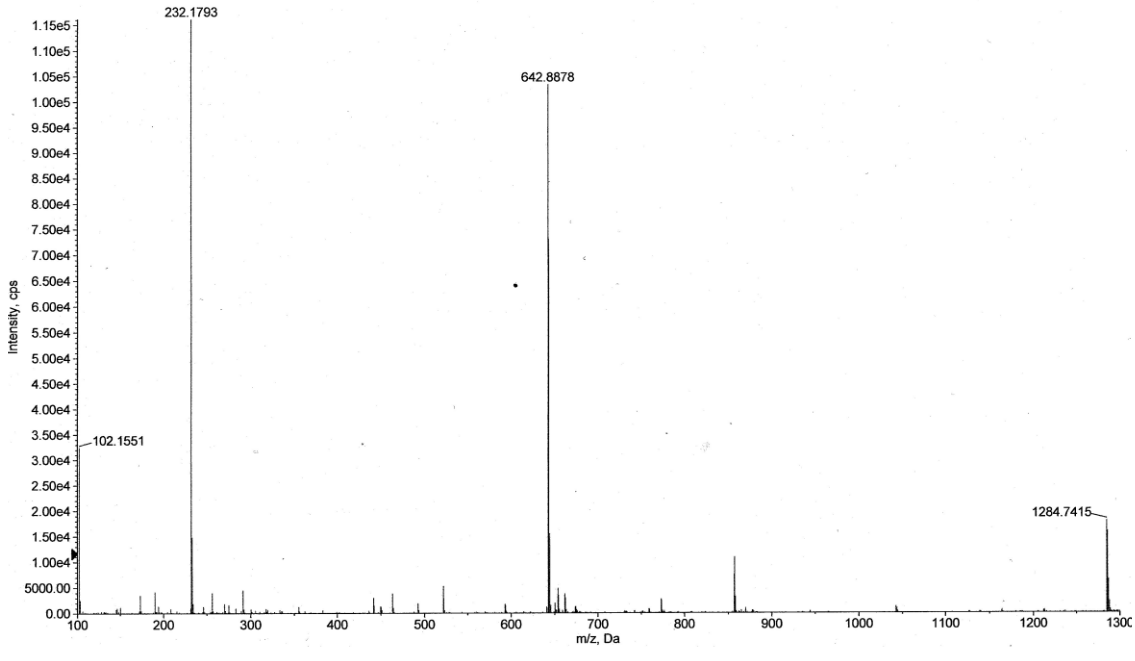

Figure S43. ESI-MS spectrum of **3B**.

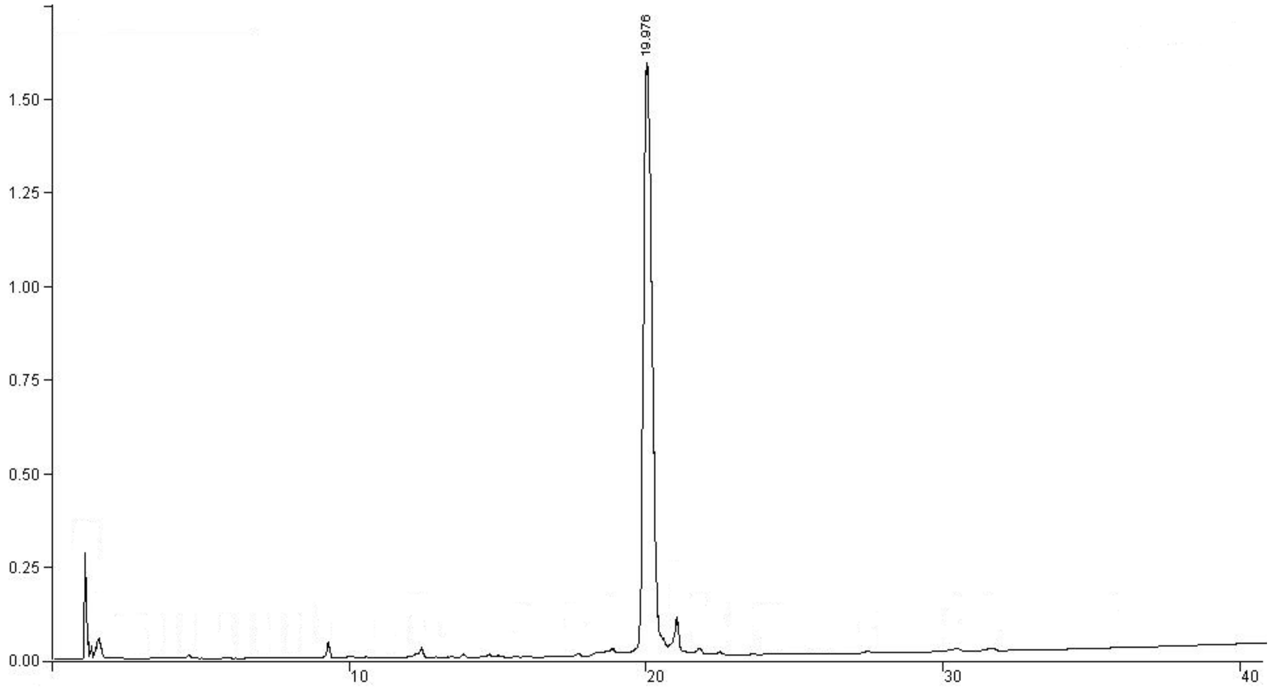

Figure S44. HPLC spectrum of **3B**.

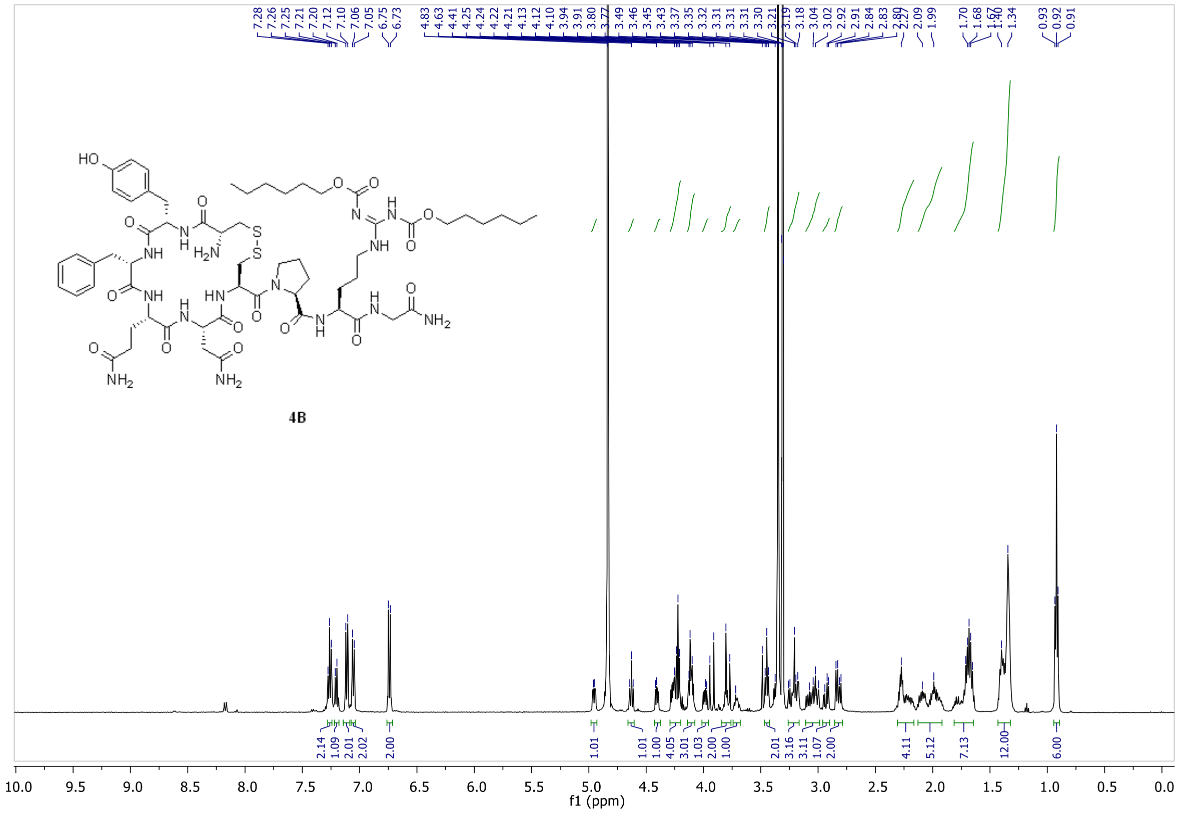

Figure S45. ^{1}H NMR spectrum of **4B**.

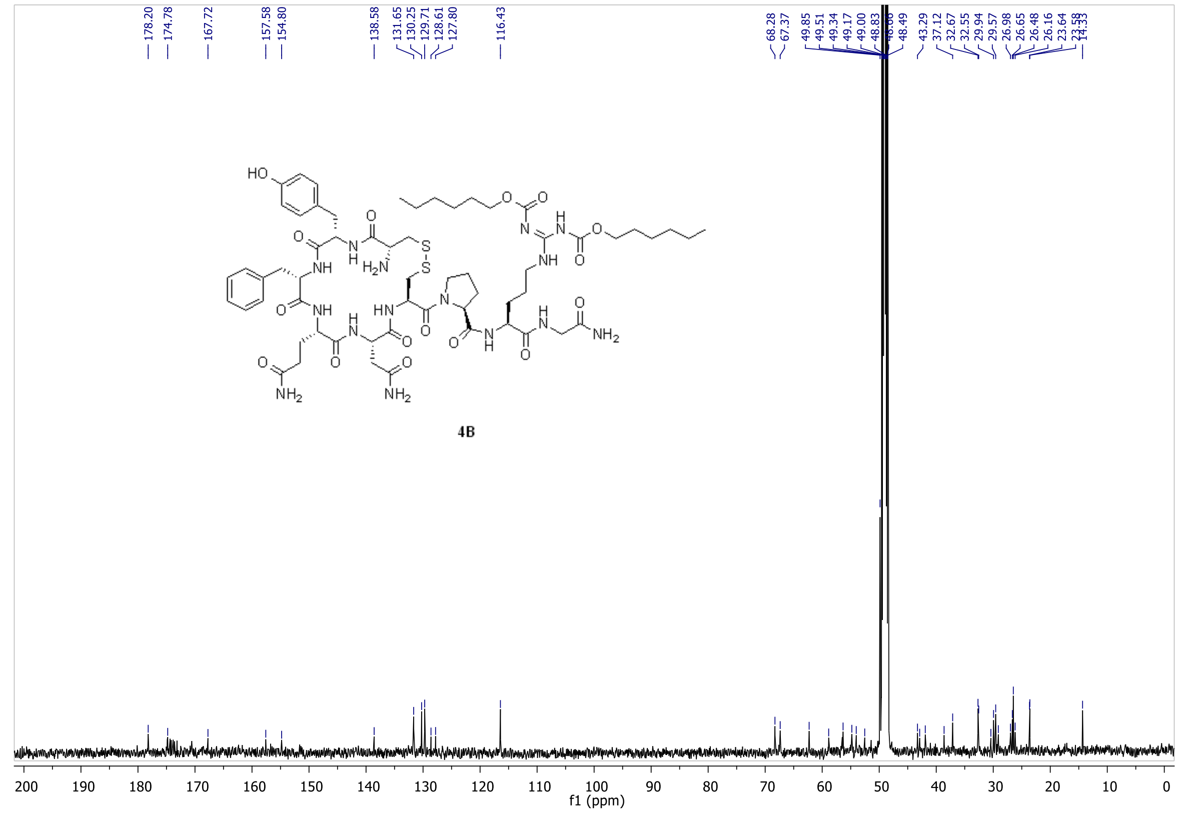

Figure S46. ^{13}C NMR spectrum of **4B**.

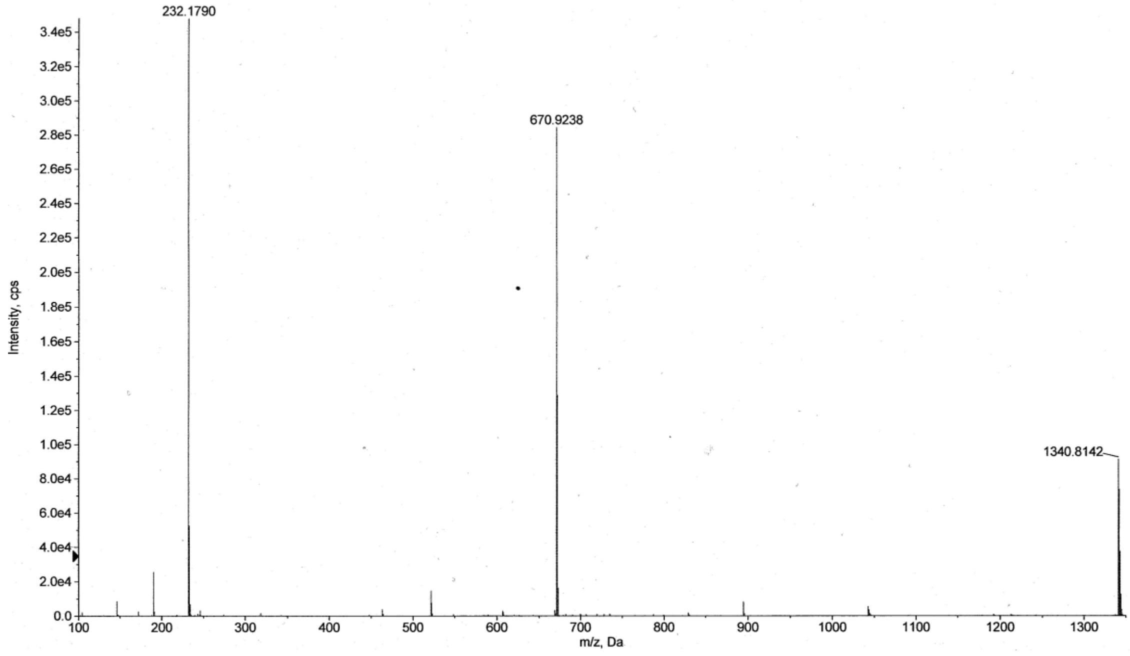

Figure S47. ESI-MS spectrum of **4B**.

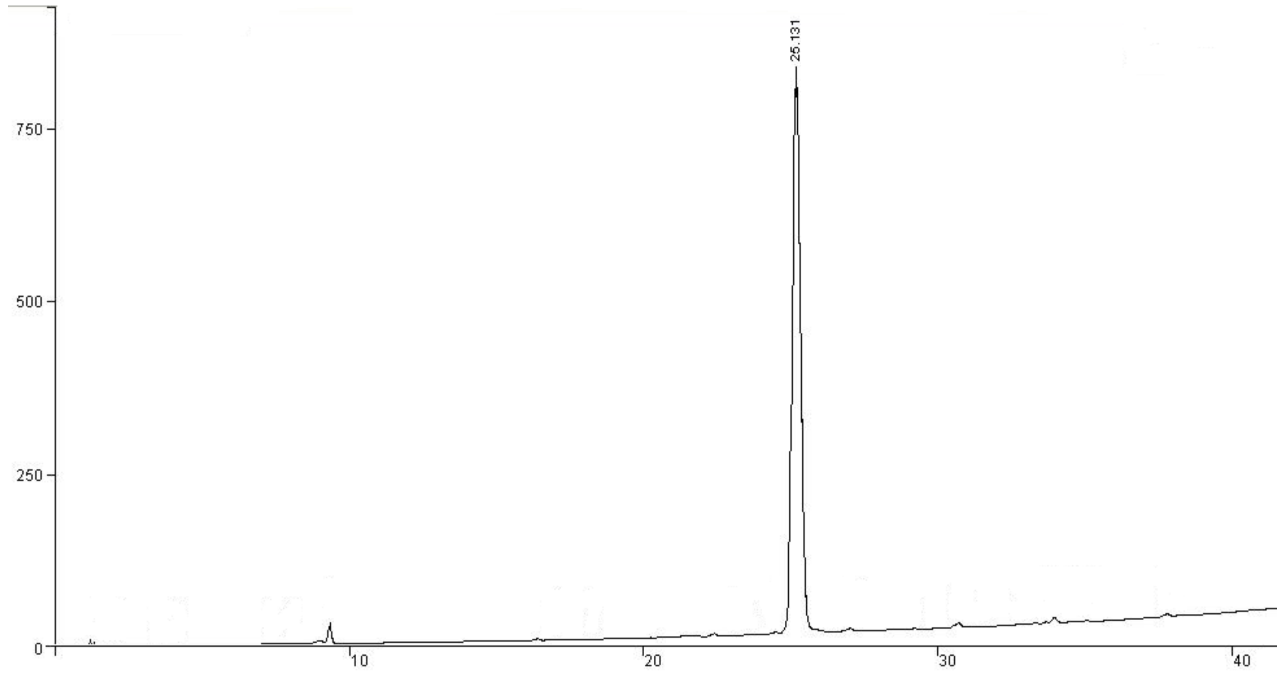

Figure S48. HPLC spectrum of **4B.**

*4.1. Reaction of **3** and **5** with iso-butylamine*

Product of **1** and *iso*-butylamine was not detected on MALDI.

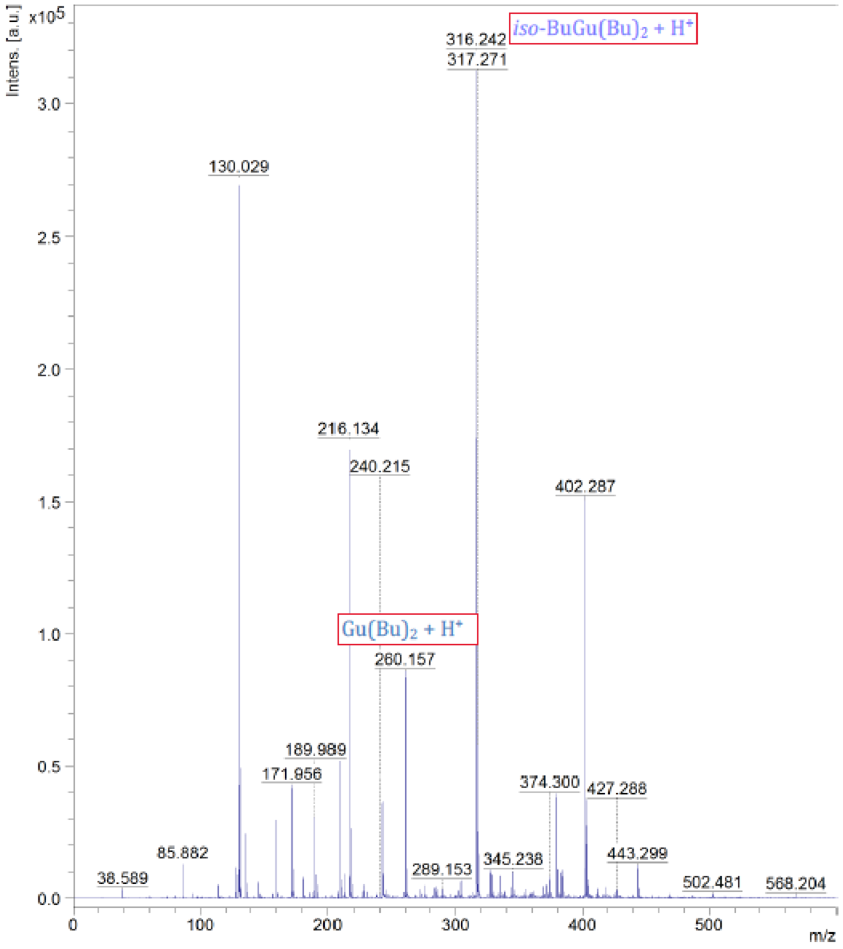

Figure S49. MALDI-MS spectrum of reaction mixture of **3** and *iso*-butylamine.

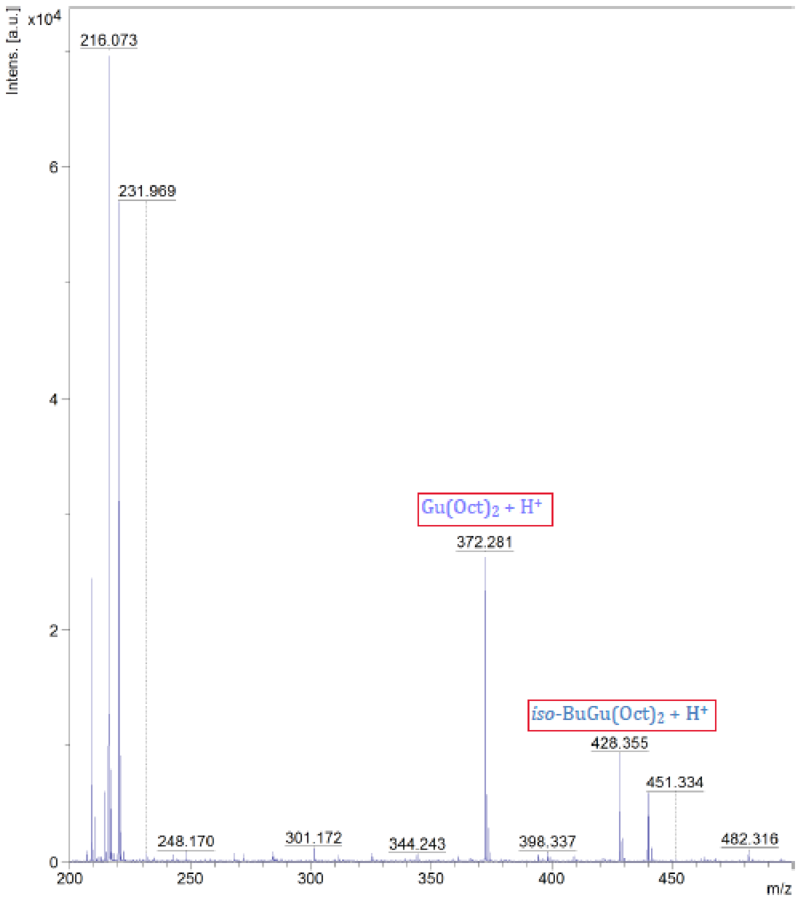

Figure S50. MALDI-MS spectrum of reaction mixture of **5** and *iso*-butylamine.

*4.2. NMR comparison of compounds **1** and **2***

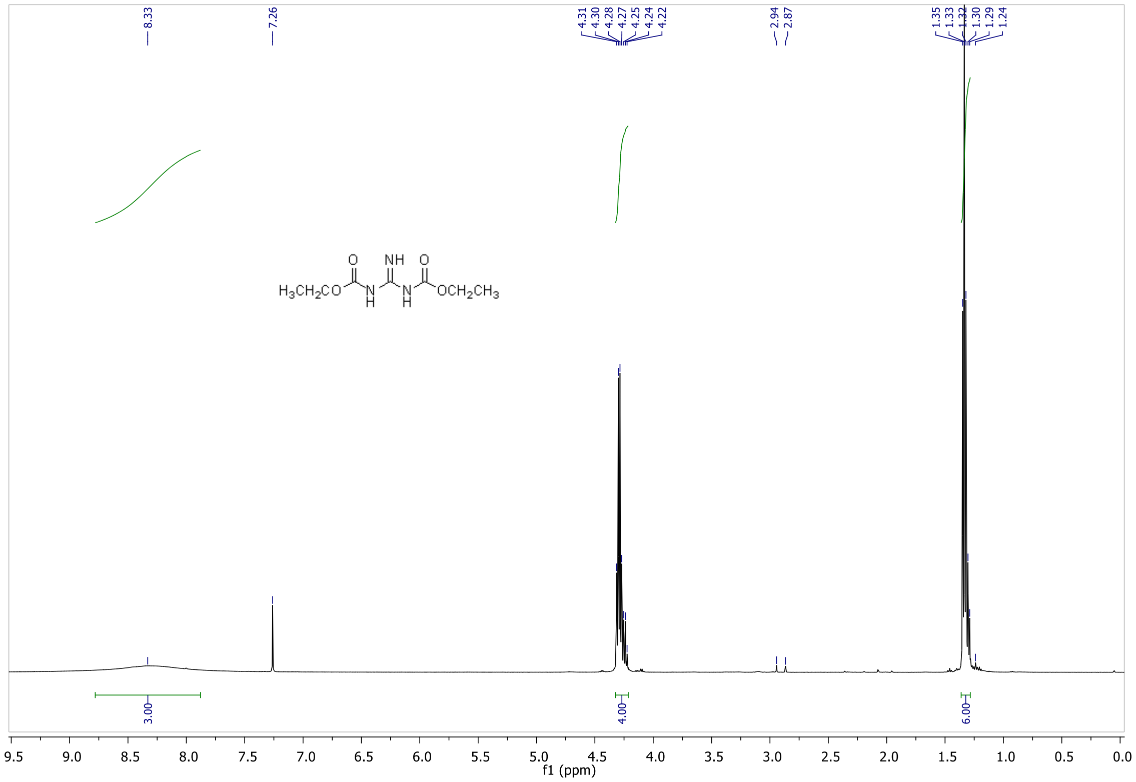

Figure S51. ^{1}H NMR ($CDCl_3$) spectrum of **1**.

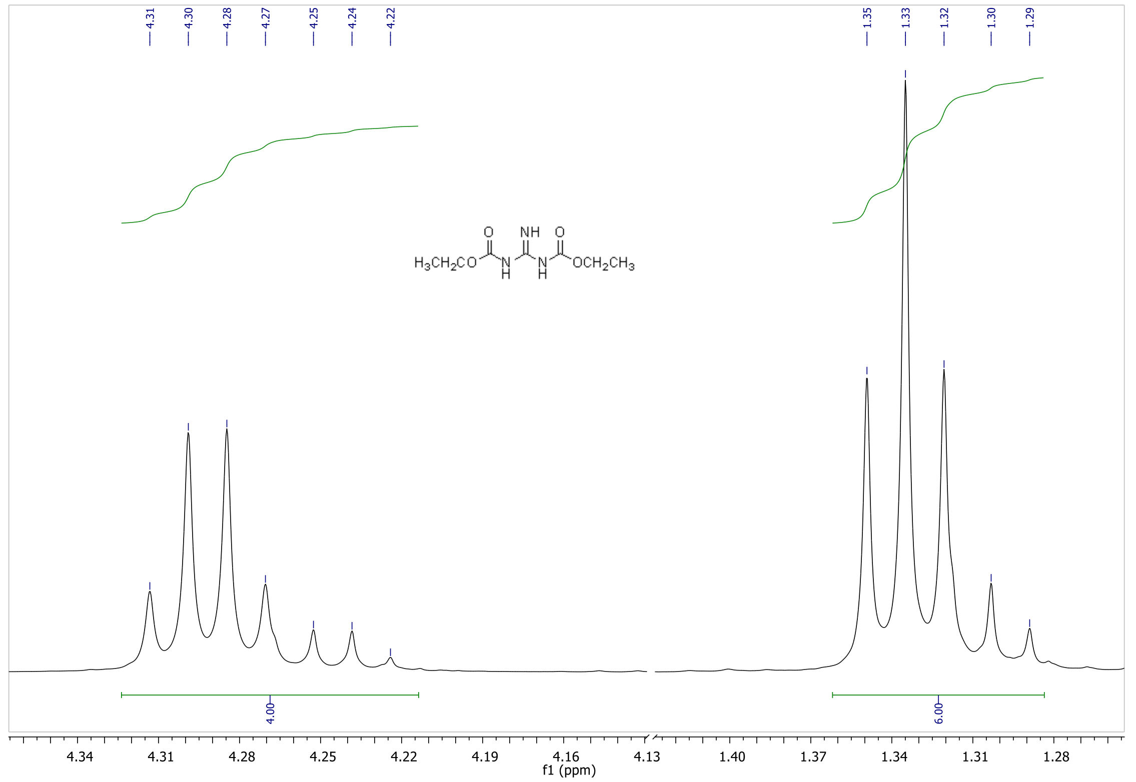

Figure S52. Enlarged view of signals in ^{1}H NMR ($CDCl_3$) for CH_3CH_2- atoms of **1**.

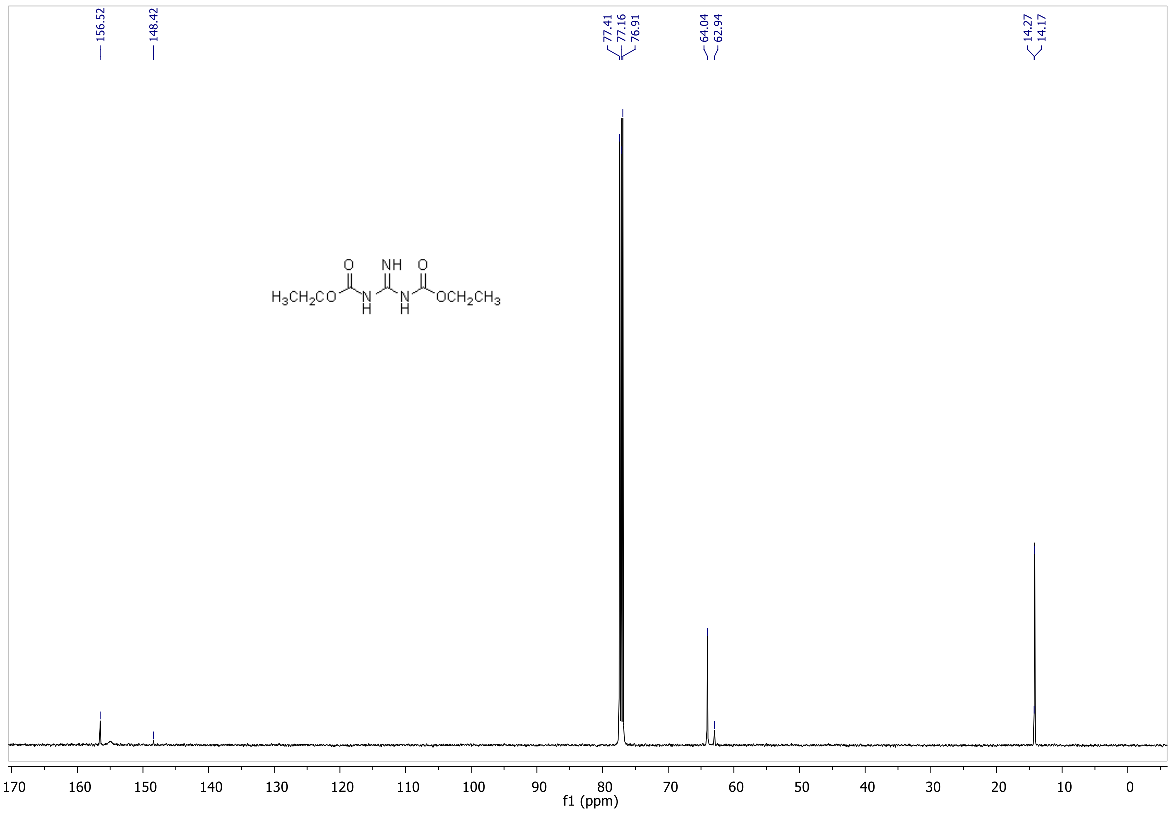

Figure S53. ^{13}C NMR ($CDCl_3$) spectrum of **1**.

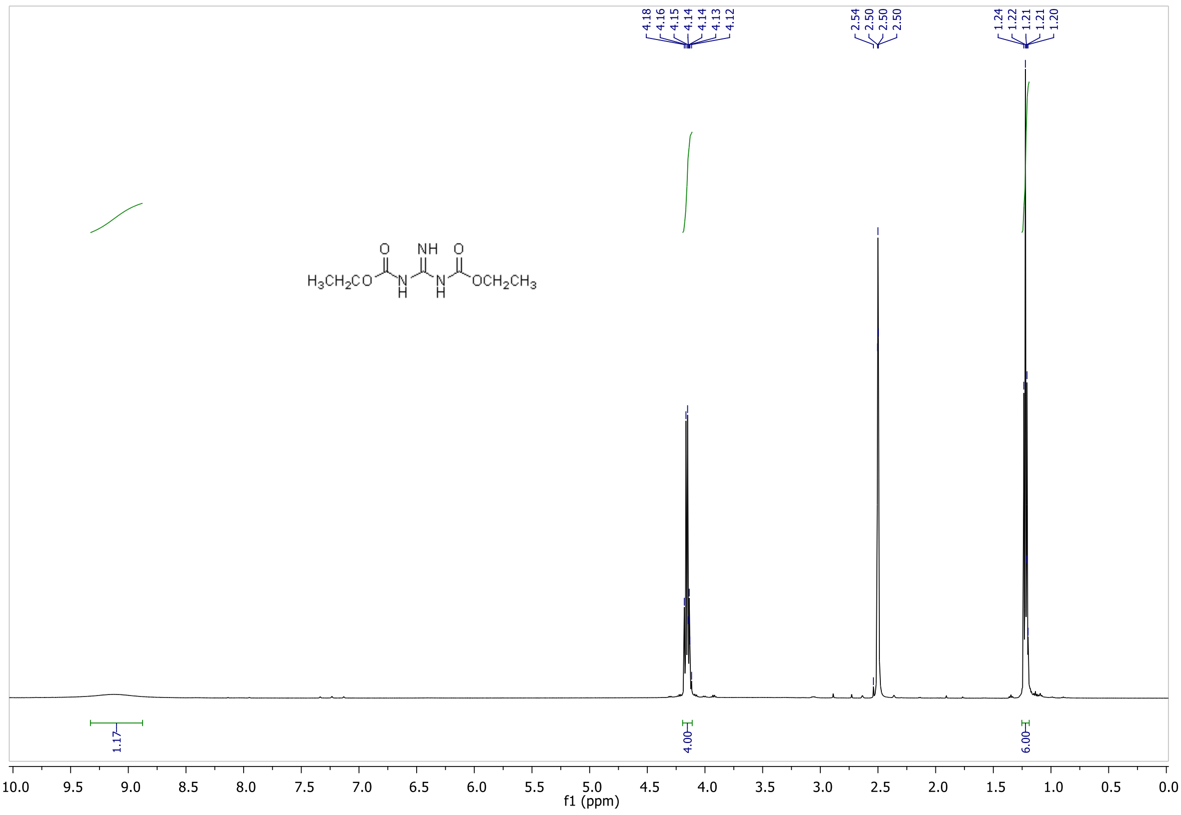

Figure S54. ^{1}H NMR (DMSO) spectrum of **1**.

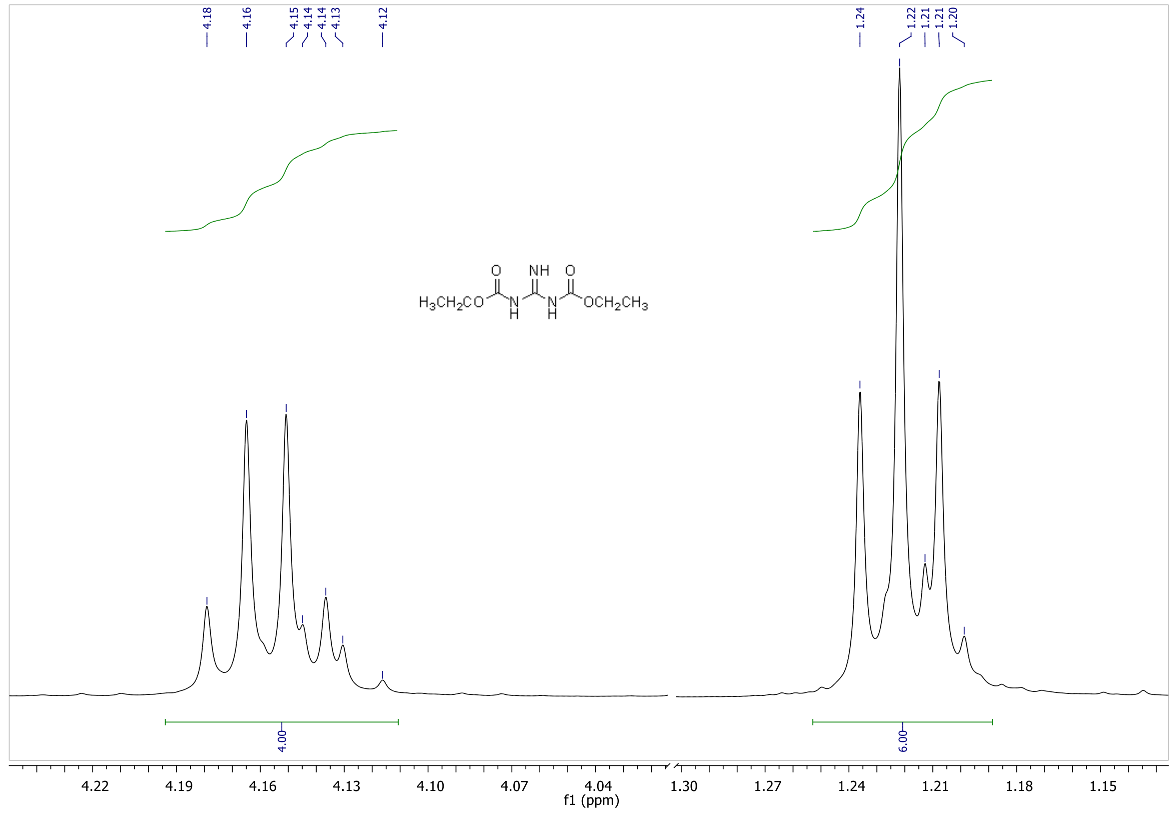

Figure S55. Enlarged view of signals in ^{1}H NMR (DMSO) for CH_3CH_2- atoms of **1**.

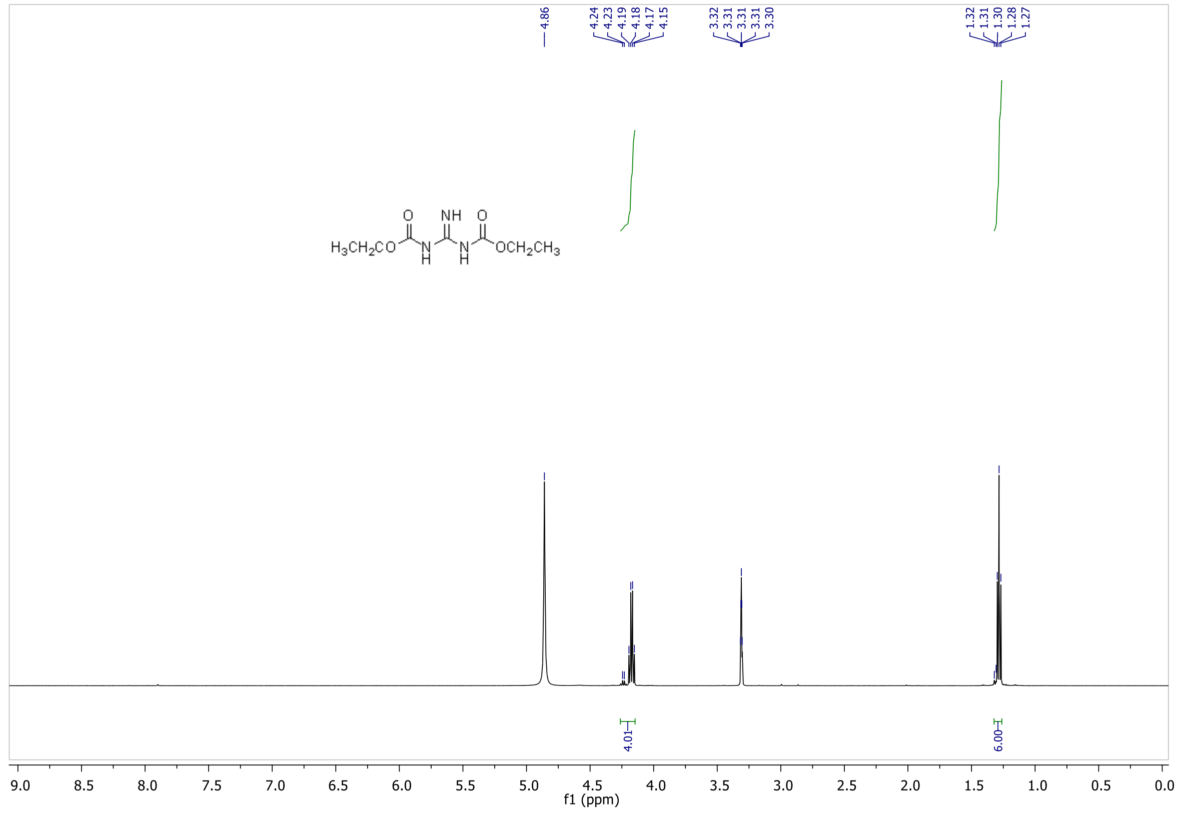

Figure S56. ^{1}H NMR (CD_3OD) spectrum of **1**.

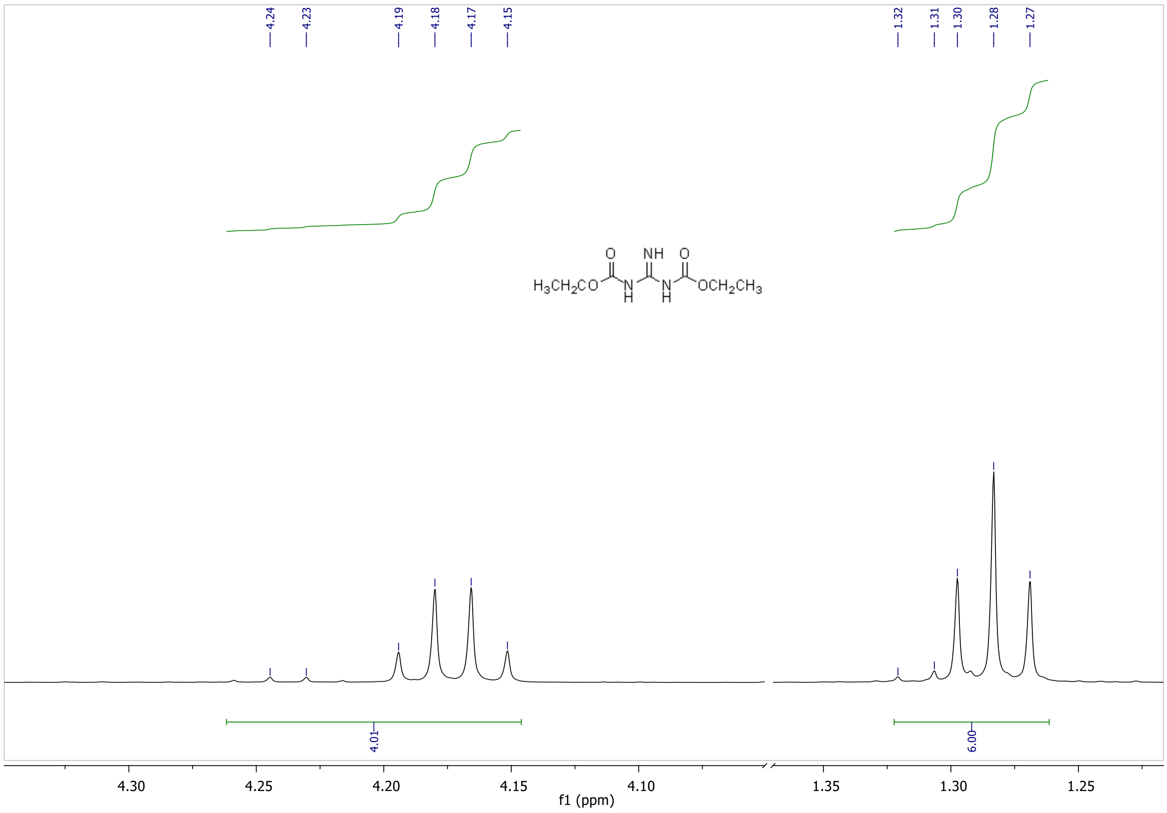

Figure S57. Enlarged view of signals in ^{1}H NMR (CD_3OD) for CH_3CH_2- atoms of **1**.

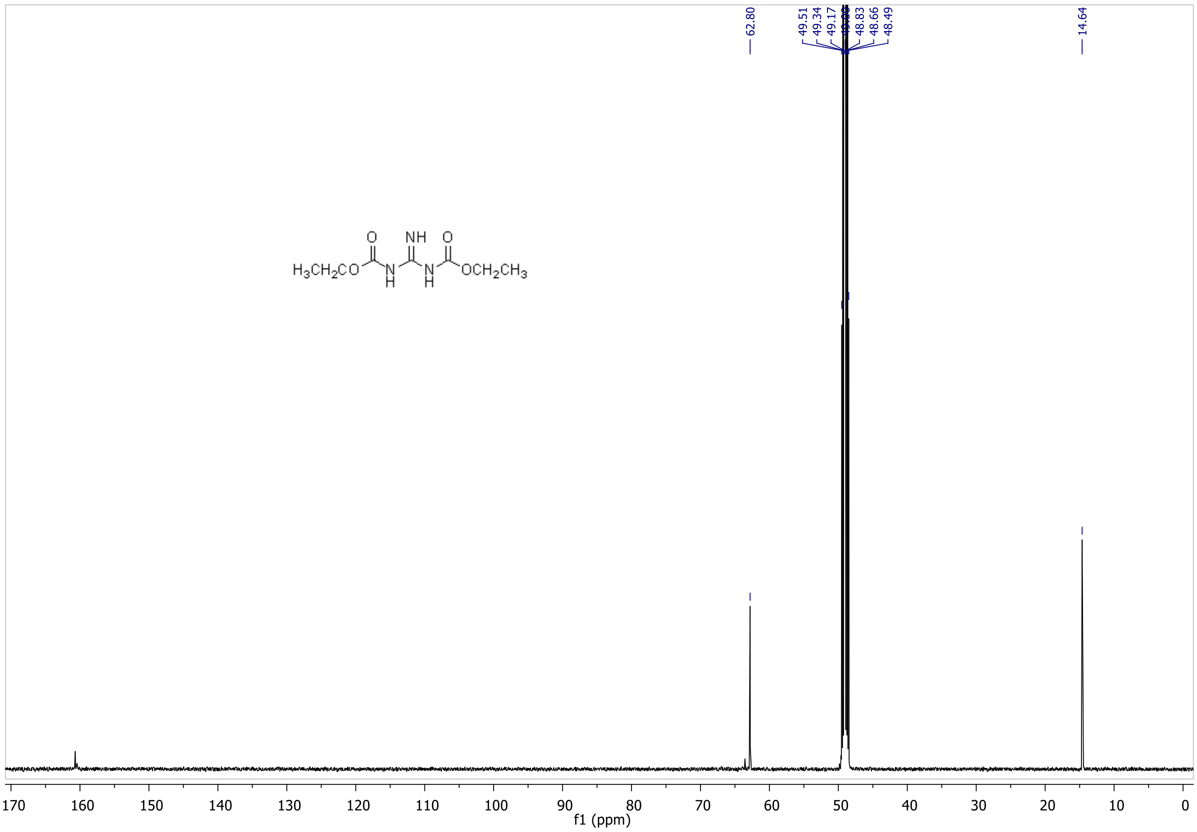

Figure S58. ^{13}C NMR (CD_3OD) spectrum of **1**.

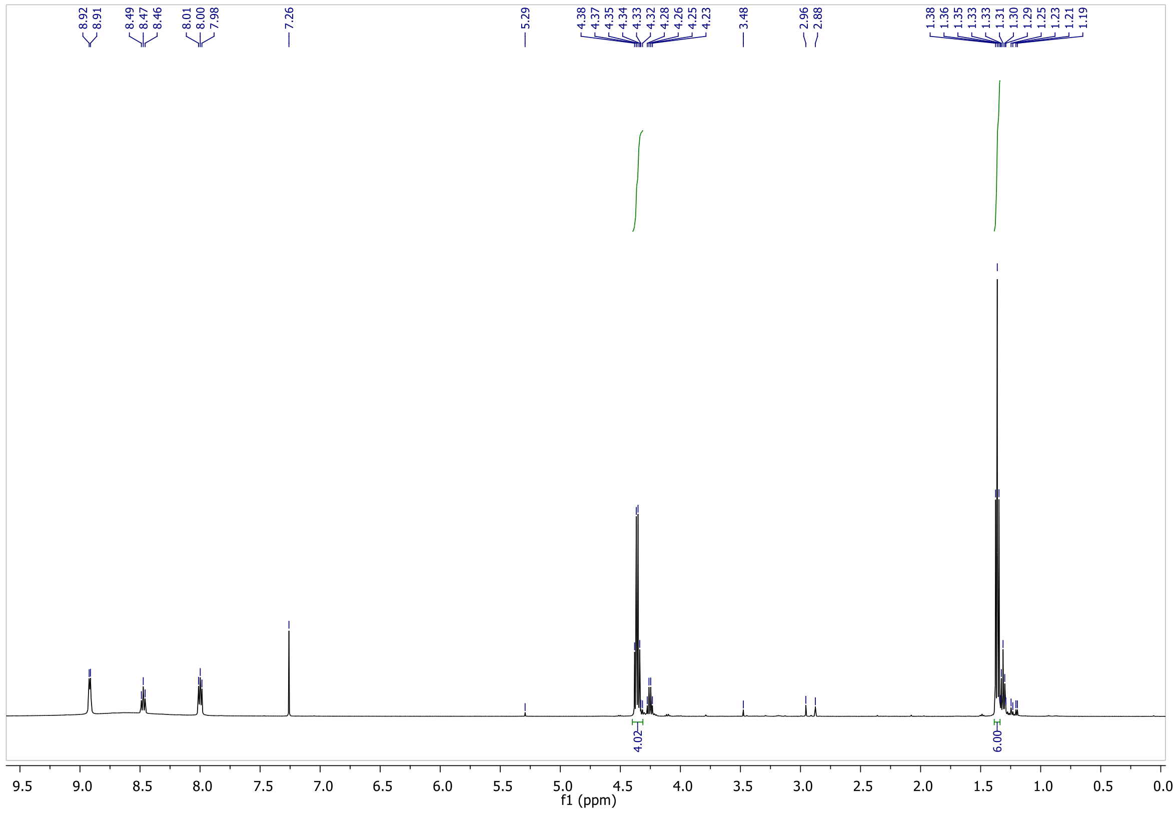

Figure S59. ^{1}H NMR ($CDCl_3$) spectrum of reaction mixture of **1**, Py and Tf_2O (step 2).

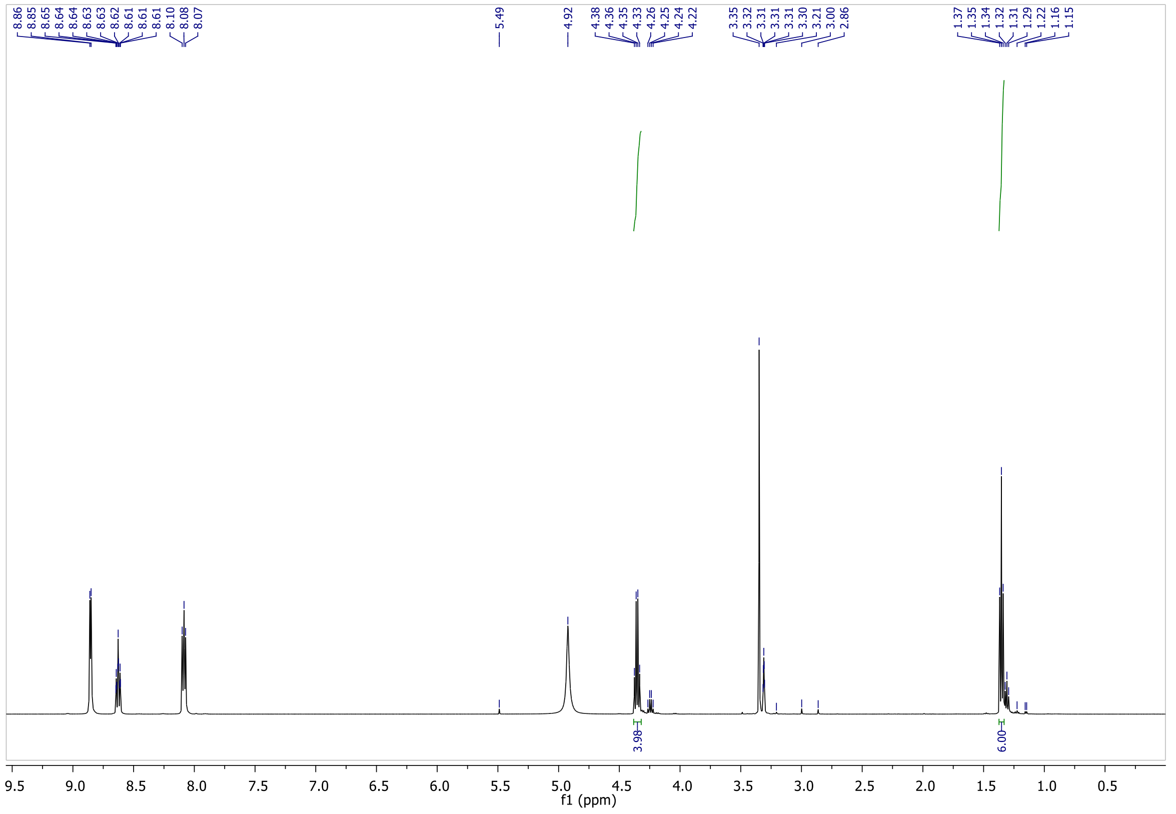

Figure S60. ^{1}H NMR (CD_3OD) spectrum of reaction mixture of **1**, Py and Tf_2O (step 2).

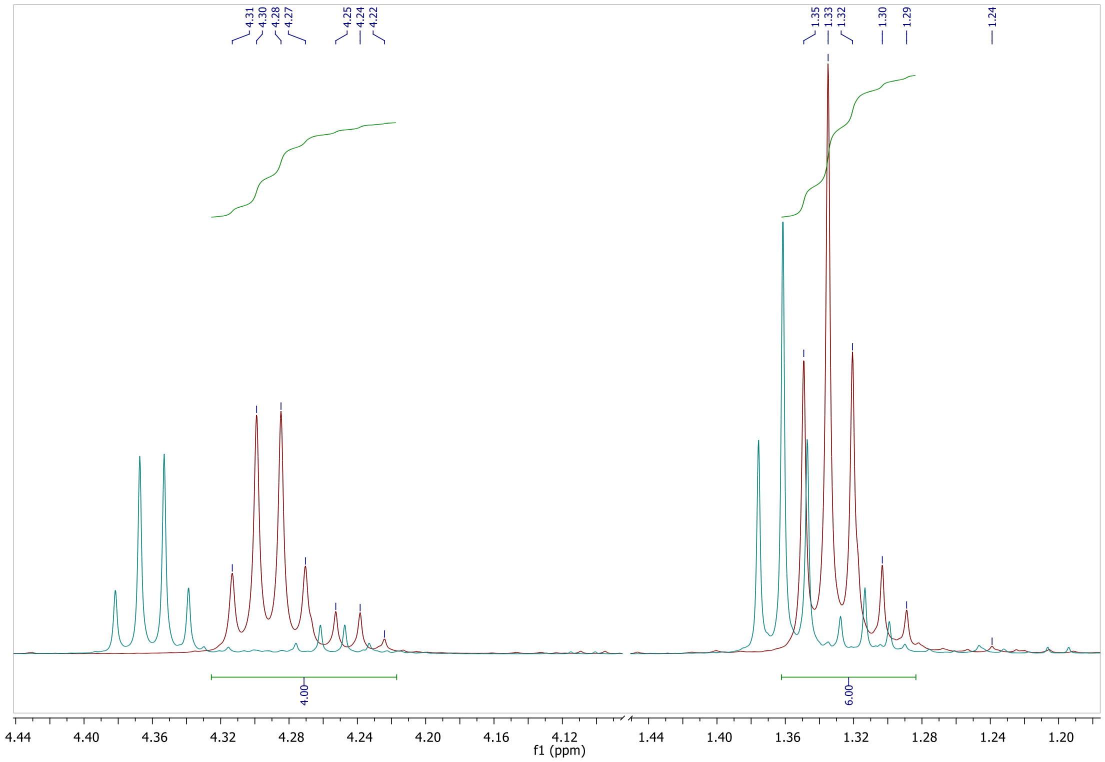

Figure S61. Enlarged view of signals comparison in ^{1}H NMR ($CDCl_3$) for CH_3CH_2- atoms of **1** (red) and reaction mixture of **1**, Py and Tf_2O (blue).

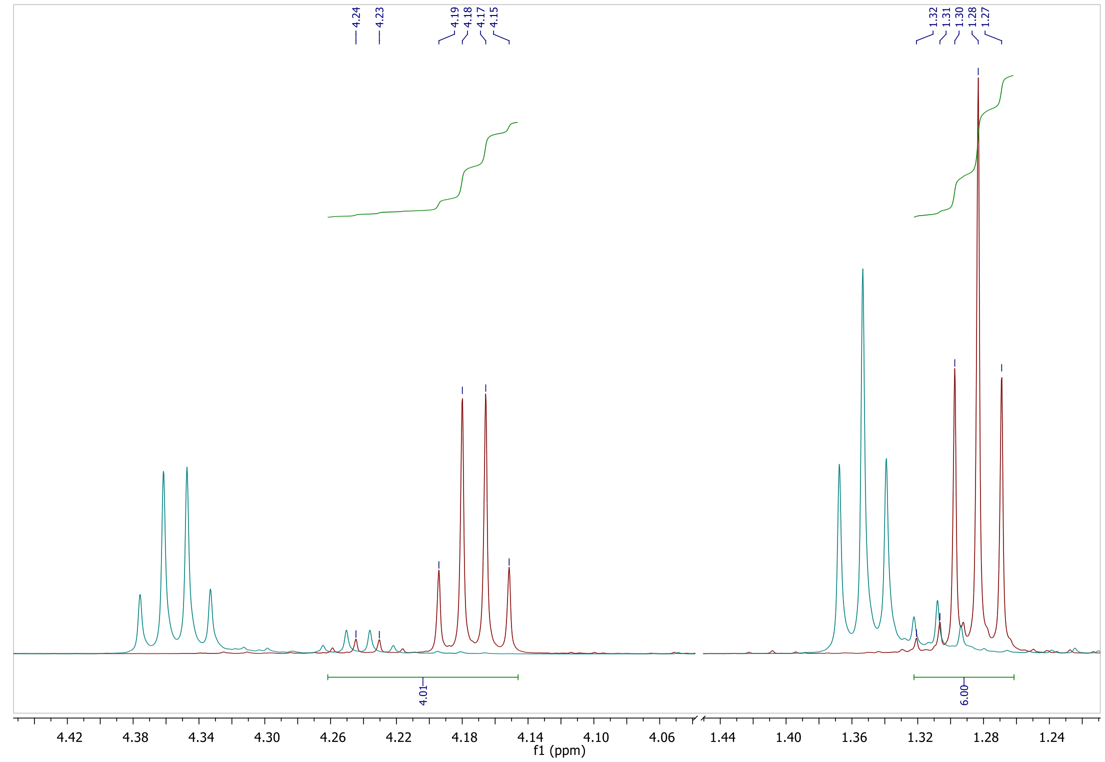

Figure S62. Enlarged view of signals comparison in ^{1}H NMR (CD_3OD) for CH_3CH_2- atoms of **1** (red) and reaction mixture of **1**, Py and Tf_2O (blue).

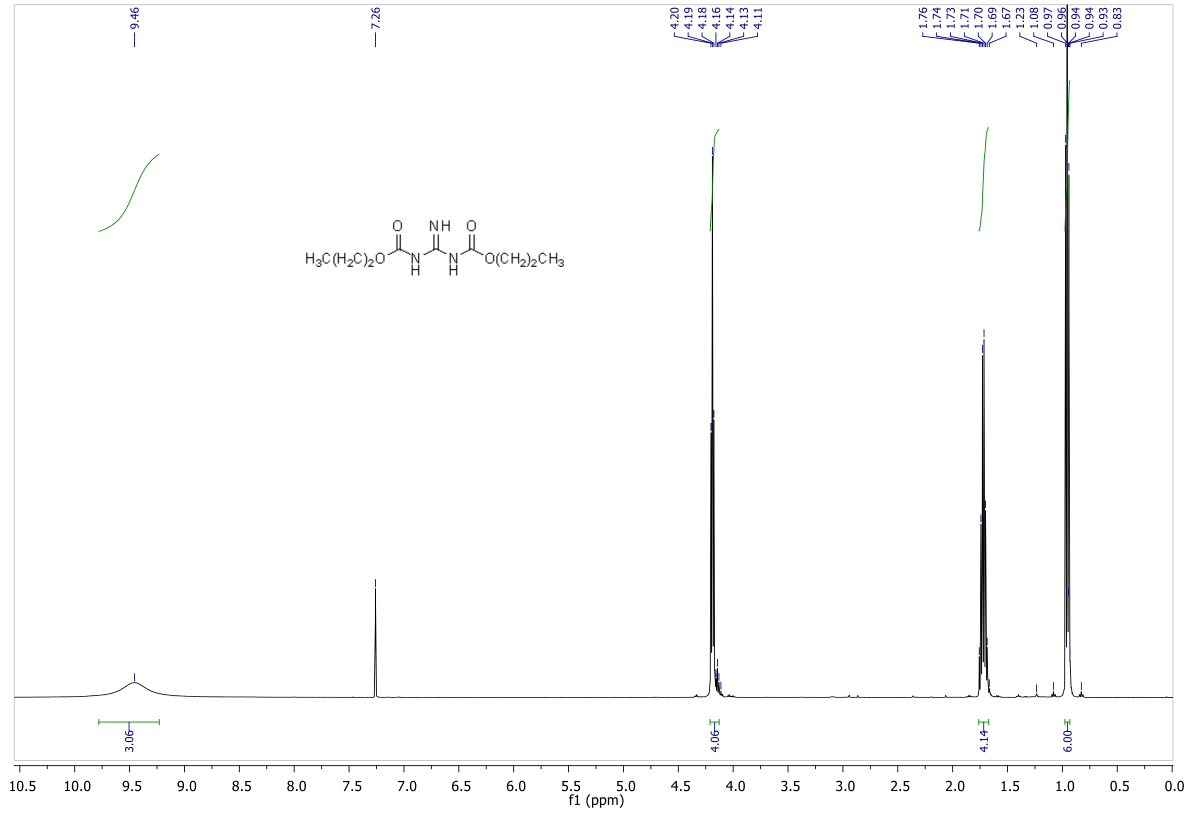

Figure S63. ^{1}H NMR ($CDCl_3$) spectrum of **2**.

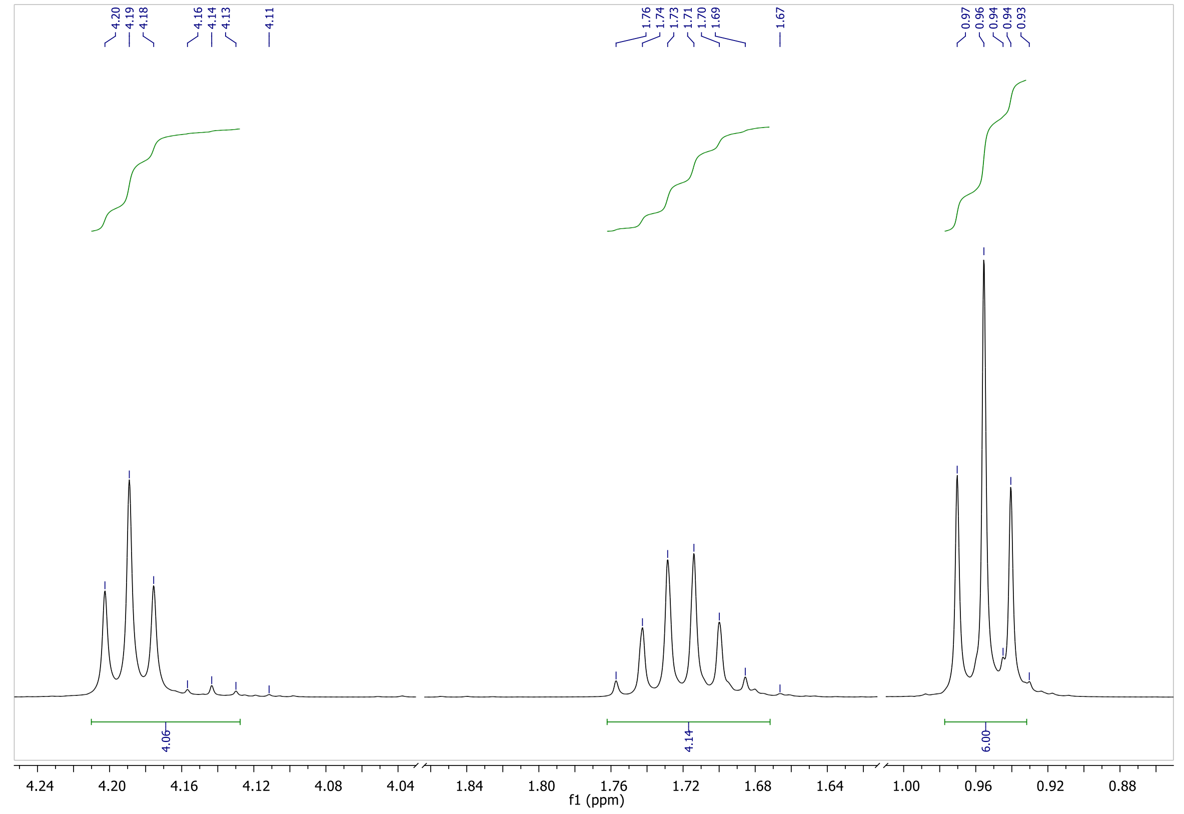

Figure S64. Enlarged view of signals in ^{1}H NMR ($CDCl_3$) for $CH_3CH_2CH_2$- atoms of **2**.

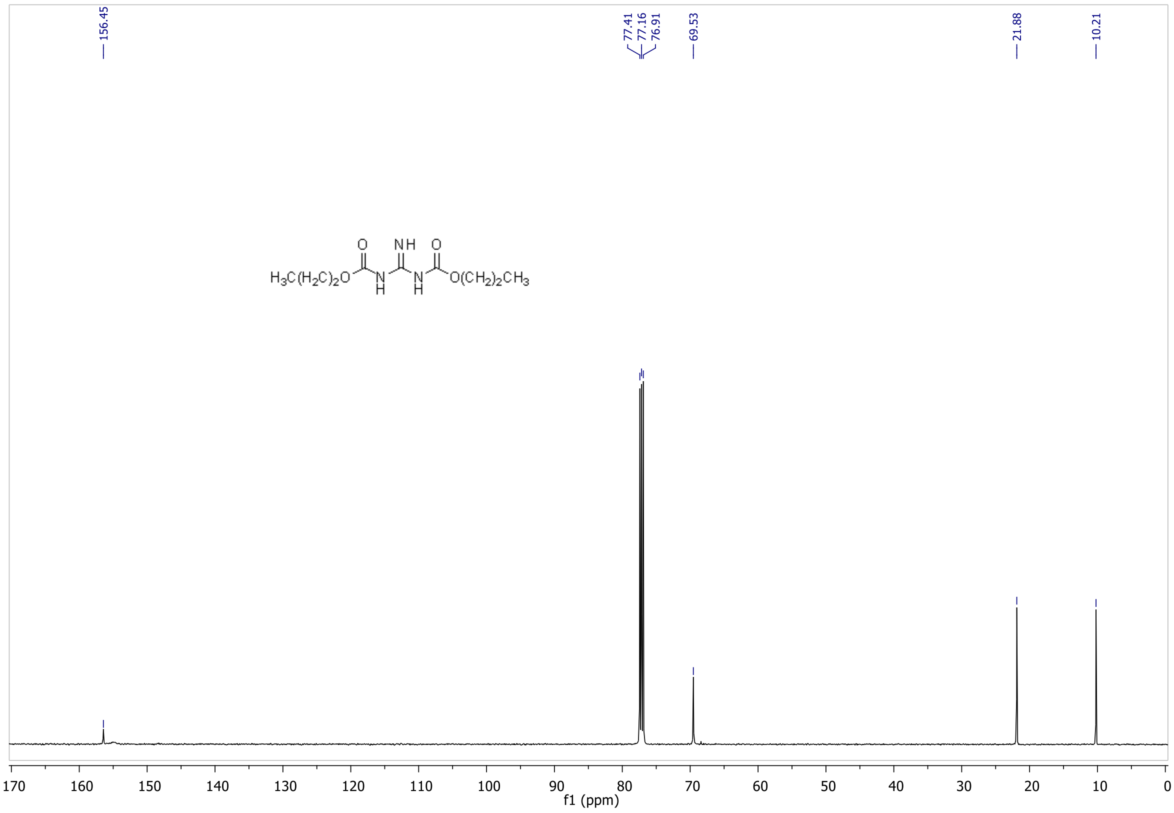

Figure S65. ^{13}C NMR ($CDCl_3$) spectrum of **2**.

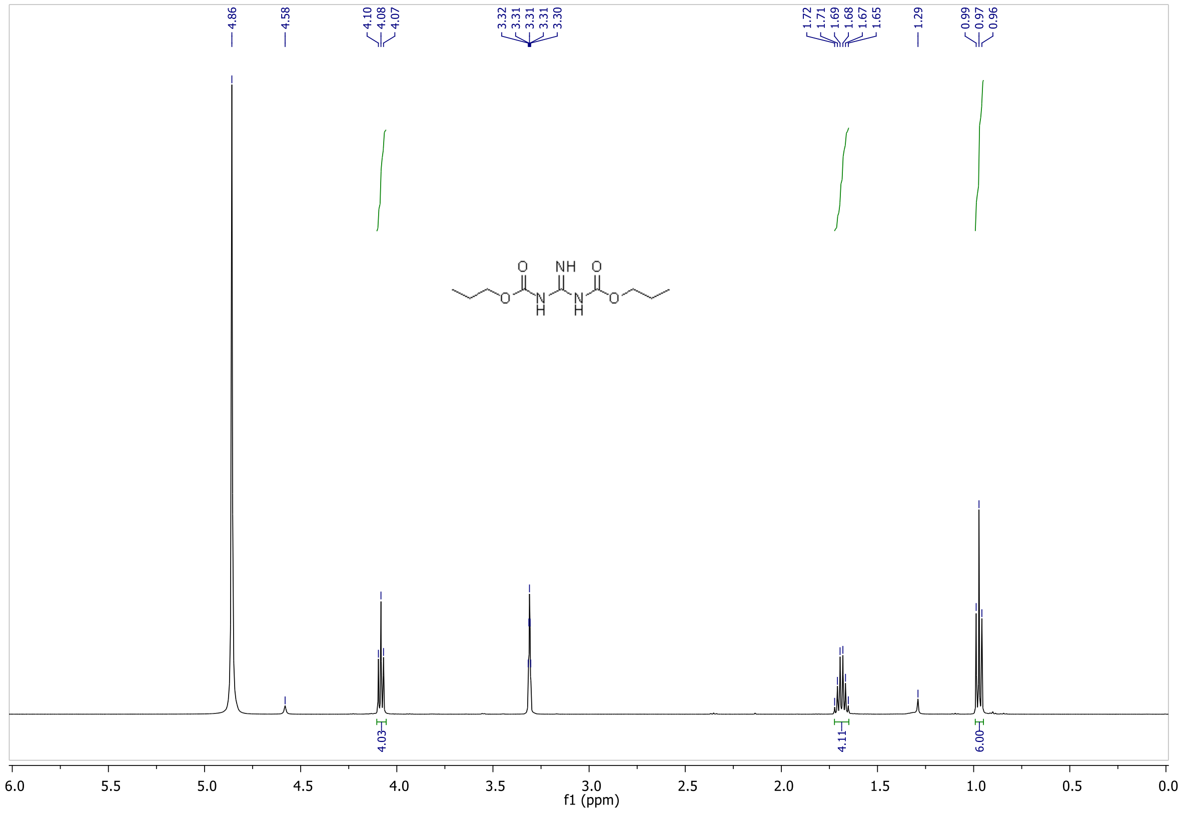

Figure S66. ^1H NMR (CD_3OD) spectrum of **2**.

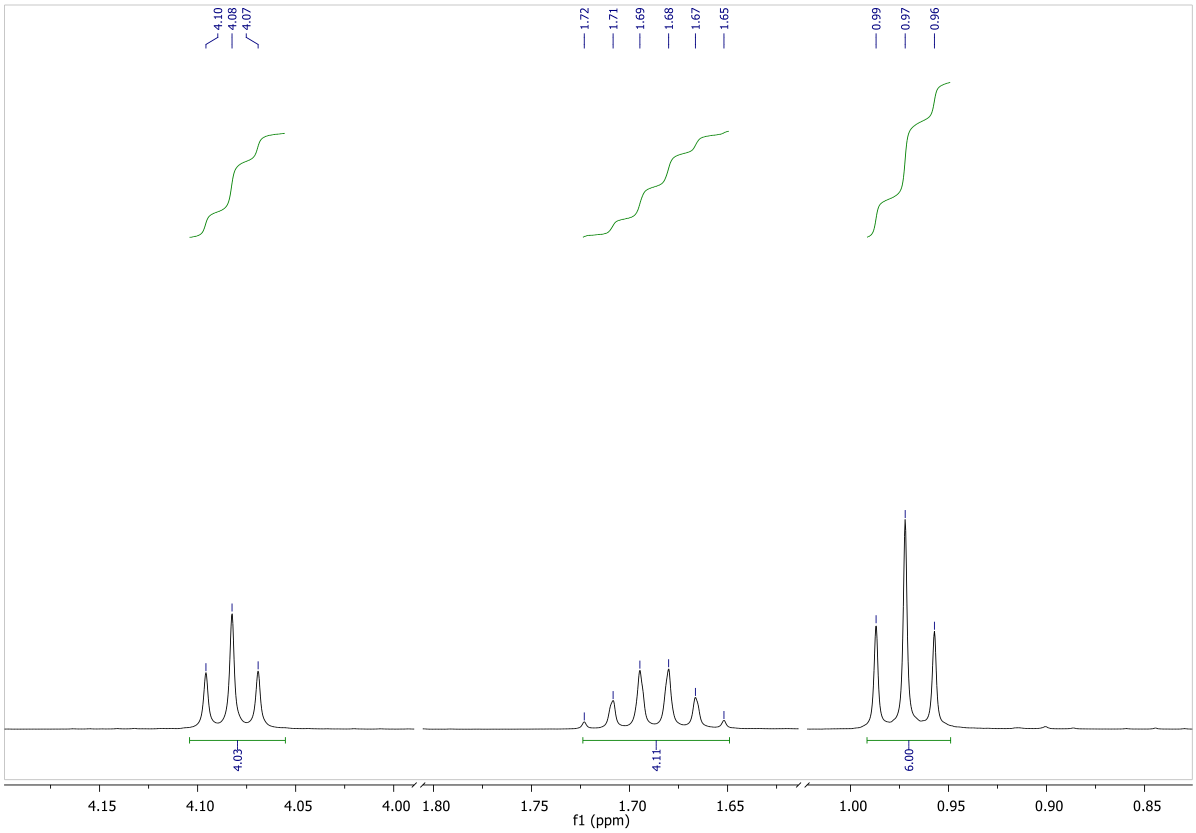

Figure S67. Enlarged view of signals in ^{1}H NMR (CD_3OD) for $CH_3CH_2CH_2$- atoms of **2**.

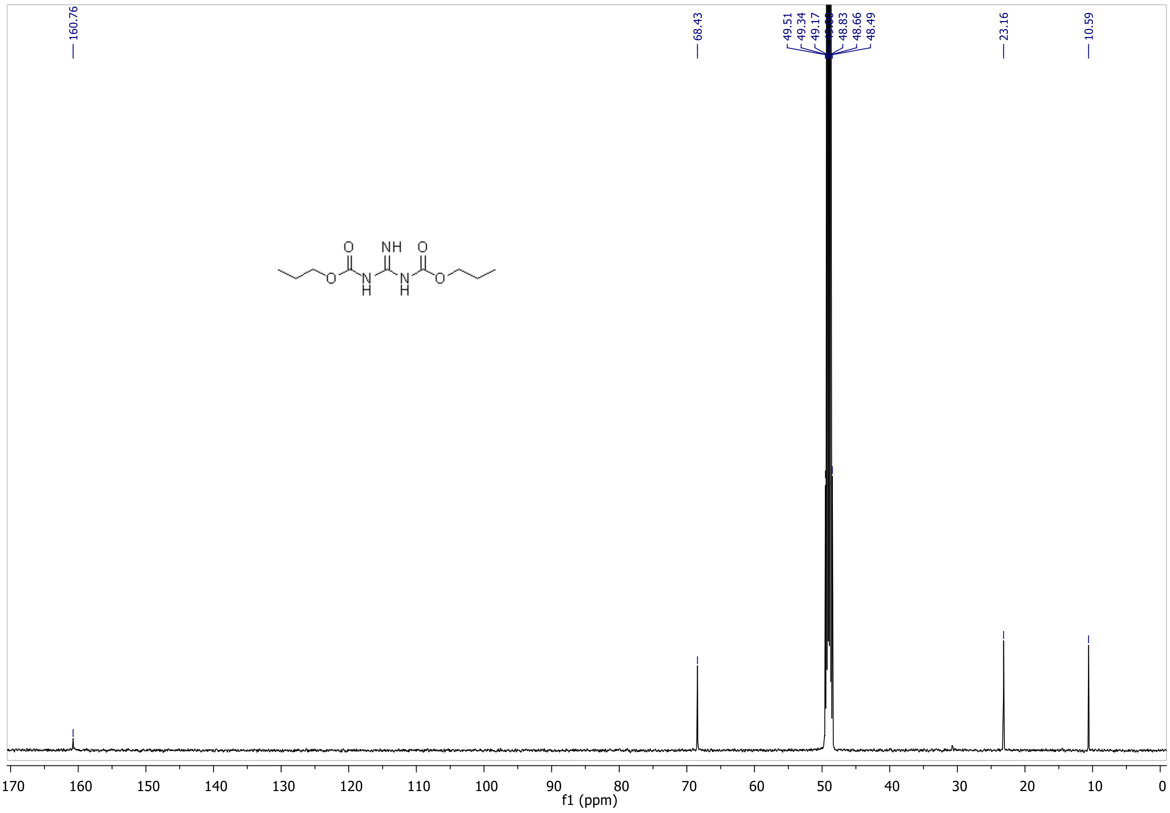

Figure S68. ^{13}C NMR (CD_3OD) spectrum of **2**.
